# Curvature induces active velocity waves in rotating spherical tissues

**DOI:** 10.1038/s41467-023-37054-2

**Published:** 2023-03-24

**Authors:** Tom Brandstätter, David B. Brückner, Yu Long Han, Ricard Alert, Ming Guo, Chase P. Broedersz

**Affiliations:** 1 Arnold-Sommerfeld-Center for Theoretical Physics, Ludwig-Maximilians-Universität München, Theresienstr. 37, 80333 Munich, Germany; 2 Department of Physics and Astronomy, Vrije Universiteit Amsterdam, 1081 HV Amsterdam, The Netherlands; 3 Institute of Science and Technology Austria, Am Campus 1, 3400 Klosterneuburg, Austria; 4 Department of Mechanical Engineering, Massachusetts Institute of Technology, Cambridge, MA USA; 5 Max Planck Institute for the Physics of Complex Systems, Nöthnitzerstr. 38, 01187 Dresden, Germany; 6 Center for Systems Biology Dresden, Pfotenhauerstr. 108, 01307 Dresden, Germany; 7 Lewis-Sigler Institute for Integrative Genomics, Princeton University, Princeton, NJ USA; 8 Princeton Center for Theoretical Science, Princeton University, Princeton, NJ USA

**Keywords:** Biological physics, Cellular motility

## Abstract

The multicellular organization of diverse systems, including embryos, intestines, and tumors relies on coordinated cell migration in curved environments. In these settings, cells establish supracellular patterns of motion, including collective rotation and invasion. While such collective modes have been studied extensively in flat systems, the consequences of geometrical and topological constraints on collective migration in curved systems are largely unknown. Here, we discover a collective mode of cell migration in rotating spherical tissues manifesting as a propagating single-wavelength velocity wave. This wave is accompanied by an apparently incompressible supracellular flow pattern featuring topological defects as dictated by the spherical topology. Using a minimal active particle model, we reveal that this collective mode arises from the effect of curvature on the active flocking behavior of a cell layer confined to a spherical surface. Our results thus identify curvature-induced velocity waves as a mode of collective cell migration, impacting the dynamical organization of 3D curved tissues.

## Abstract (web summary)

The existence of multicellular systems relies on coordinated cell motion in three dimensions. Here, cell migration in rotating spherical tissues is shown to exhibit a collective mode with a single-wavelength velocity wave, which arises from the effect of curvature on the flocking behavior of cells on a spherical surface.

## Introduction

Collective cell migration in physiological processes ranging from development^1^ to cancer^2^ take place in curved geometries^3^. A prominent manifestation of curvature in cellular tissues is the conceptually simple spherical geometry. This geometry arises naturally in a number of in vivo systems, including blastocysts^1^, egg chambers^4^, and tumors^2^. Furthermore, the consequences of a spherical geometry for collective migration can be studied in vitro in systems such as epithelial spheroids^5–8^, as well as intestinal^9^, pancreas^10^, and cerebral^11^ organoids. Like cells in 2D circular confinements^12–15^, migrating cells in 3D spherical confinements often exhibit collective behaviors such as rotational motion^4,8,16–21^. A variety of collective cell behaviors have been successfully described using active matter theories based on active self-propulsion and alignment interactions between cells^22–25^. Therefore, collectively migrating cells can be placed within a broader class of active matter systems^26^, ranging from motile cytoskeletal filaments^27,28^ to swarming midges^29^, and flocks of birds^30^. Importantly, unlike in 2D, collective cell migration in 3D confinements may be subject to physical constraints imposed by the geometrical curvature^31–37^ and the topology of the system. Indeed, recent theoretical studies on nematic^38,39^ and polar^40–43^ active matter on spherical surfaces illustrate how ordering, collective behaviors, and motion patterns are drastically affected by curvature due to fundamental symmetry and topology principles^44,45^. For example, non-interacting self-propelled particles move along geodesics that are shaped by the curvature of the system. In addition, the topology of spherical systems prevents states with uniform orientational order, resulting in the formation of topological defects. However, the effects of these geometrical and topological constraints on collective cell migration in inherently curved 3D systems are not understood. These physical constraints may have consequences for the constantly evolving multicellular architecture of the tissue, which controls biological processes ranging from development^1^ to cancer progression^2^. Identifying the basic principles of collective migration in 3D curved geometries is thus central for understanding how tissues form, develop shape, and maintain homeostasis.

## Results

### Spheroids perform stochastic global rotations

**Fig. 1:**
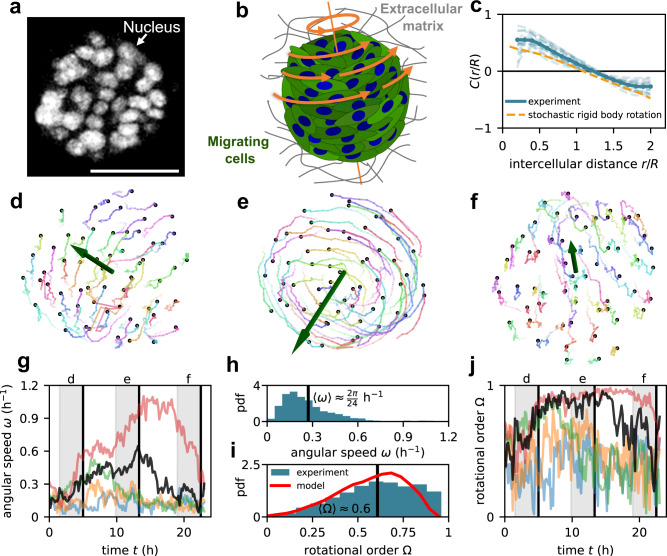
Stochastic global rotations of spheroids. **a** Fluorescence microscopy image of a spheroid with fluorescently labeled (GFP-NLS) nuclei (see the “Methods” section). Scale bar $50\,{{{{{\rm{\mu }}}}}}{{{{{\rm{m}}}}}}$. **b** Sketch of a rotating spheroid in its extracellular environment. **c** Spatial correlation function of velocity directions $C(r/R)$, where $r$ is the distance between two cells throughout the spheroids and $R$ is the spheroid radius (see the “Methods” section). Experimental curves for individual spheroids (light blue) and their average (blue) are compared with the correlations of a stochastic rigid-body rotation (orange) (Supplementary Section 2). **d–f** Time series of cell trajectories in a spheroid. Green vectors show the instantaneous angular velocity vector ${{{{{\boldsymbol{\omega }}}}}}$ of the collective rotation. **g** Time evolution of angular speed $\omega=|{{{{{\boldsymbol{\omega }}}}}}|$ in five different spheroids. The black curve shows the angular speed of the spheroid shown in (**d**–**f**). Gray regions indicate the time period of the trajectories shown in (**d**–**f**). **h**, **i** Distributions of angular speed $\omega$ (**h**) and rotational order $\Omega$ (**i**). Both distributions are across both time and different spheroids. Red line in (**i**) indicates the model result. **j** Time evolution of rotational order $\Omega$ in five different spheroids.

Here, we study spherical tissues (spheroids) consisting of human mammary cells (MCF10A) as a model system for collective cell migration in curved 3D geometries. The spheroids are embedded in an alginate and Matrigel-based extracellular matrix. Starting from a single cell, within 5 days of proliferation, a large spheroid is formed comprising of the order of 100 cells with a roughly spherical shape and radii in the range $R\,\approx \,15-40\,{{{{{\rm{\mu }}}}}}{{{{{\rm{m}}}}}}$ (Fig. 1a, b). These cells are highly motile and migrate in a coordinated fashion, making these spheroids ideal for studying collective modes of 3D cell migration in spherical geometries (Supplementary Movie 1).

To characterize the migratory dynamics, we perform 3D tracking of the trajectories of the cell nuclei throughout each spheroid. These trajectories reveal prominent global rotations of the spheroids, as observed previously^4,8,16–21^, albeit with significant fluctuations (Fig. 1d–f, Supplementary Movie 2). To characterize these stochastic collective rotations, we first calculate the correlation of velocity orientations as a function of intercellular distance $r$*:* $C\left(r\right)={\langle \hat{{{{{{\boldsymbol{v}}}}}}}({{{{{{\boldsymbol{r}}}}}}}_{i})\cdot \hat{{{{{{\boldsymbol{v}}}}}}}({{{{{{\boldsymbol{r}}}}}}}_{j})|{r}_{{ij}}=r\rangle }_{i\ne j}$, where $\hat{{{{{{\boldsymbol{v}}}}}}}\left({{{{{{\boldsymbol{r}}}}}}}_{i}\right)$ is the velocity orientation of the $i$th cell, and we condition on the intercellular distance ${r}_{{ij}}$ (see the “Methods” section). The correlation is positive at short distances, indicating that nearby cells move in a similar direction (Fig. 1c). At long distances, however, the correlation becomes negative, signifying that cells on different sides of the spheroid ($r\,\approx \,2R$) move in opposite directions. The distance dependence of these correlations agrees with that expected for a global rigid-body rotation (Supplementary Section 2). Such global rotations can be characterized by the spheroid’s instantaneous angular speed $\omega$ and rotational order parameter $\Omega$ quantifying alignment of rotational motion (see the “Methods” section). Both these quantities show that, despite their stochastic cell motion, the spheroids exhibit coherent and persistent global rotations (Fig. 1g–j).

### Rotating spheroids exhibit velocity waves

**Fig. 2:**
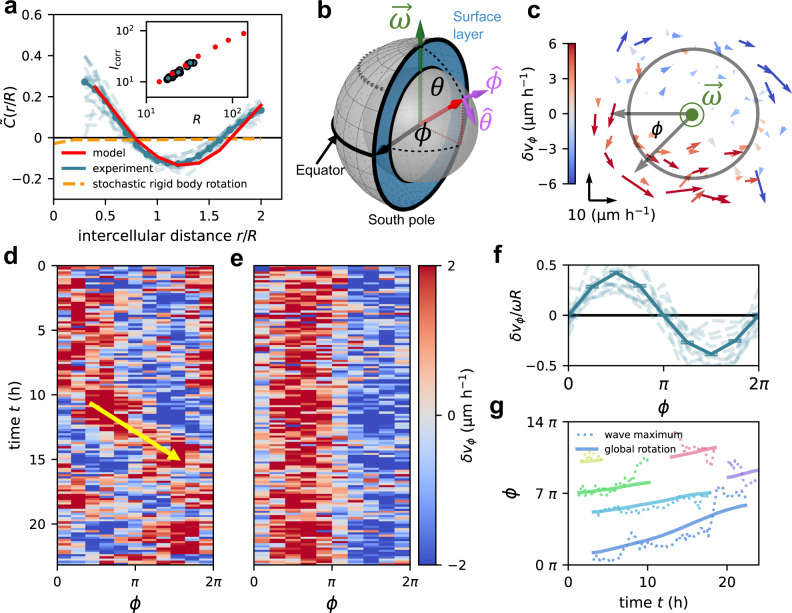
Velocity waves in rotating spheroids. **a** Spatial correlation function of velocity fluctuation directions $\widetilde{C}(r/R)$. The intercellular distance $r$ is rescaled by the spheroid radius $R$. Dashed orange line shows the correlation function for a rigid-body rotation with additional uncorrelated Gaussian noise (Supplementary Section 2). Solid red line shows the model result. Inset shows the correlation length ${l}_{{{{{{\rm{corr}}}}}}}$ as a function of spheroid radius for both the experiment (blue) and the model (red). **b** Schematic of the spherical coordinate system. The blue region indicates the cross section of the surface layer of the spheroids. The core region is indicated by the gray inner sphere. We choose the threshold between core region and surface layer at $r=0.6R$. Schemati**c** created with Geogebra. **c** Planar projection of the velocity fluctuation field $\delta {{{{{\boldsymbol{v}}}}}}$ in a slab centered around the equatorial plane. The color code indicates $\delta {v}_{\phi }$, the $\phi$-component of $\delta {{{{{\boldsymbol{v}}}}}}$, and the arrow scale is shown in the lower left corner. The gray circle indicates the threshold between the core and the surface layer. **d**, **e** Kymograph of the azimuthal component of velocity fluctuations, $\delta {v}_{\phi }\left(\phi,t\right)$, along the equator of one spheroid, both in the COM frame (**d**) and in the frame co-moving with the wave (**e**) (see the “Methods” section). **f** Velocity fluctuation profile in the equator rescaled by the average angular speed $\omega$ and the spheroid radius $R$ and averaged over all spheroids and time points. Error bars represent the standard error of the mean (s.e.m.) and dashed lines show individual profiles averaged over 20-time points. **g** Wave propagation in different spheroids. The solid line shows the integrated angular speed giving the total path length of the global rotation. Dashed lines indicate the trajectory of the propagating velocity wave maximum. We consider periods in time during which the axis of rotation of the spheroids is approximately fixed over a minimum of 15-time points (Supplementary Section 1.5).

We next ask whether these spheroids exhibit additional collective migration modes beyond the global rotation. To this end, we study velocity fluctuations $\delta {{{{{\boldsymbol{v}}}}}}$ of cells around the global rotation (see the “Methods” section). These velocity fluctuations are characterized by a non-monotonic correlation function $\widetilde{C}\left(r\right)=\,{\langle \delta \hat{{{{{{\boldsymbol{v}}}}}}}({{{{{{\boldsymbol{r}}}}}}}_{i})\cdot \delta \hat{{{{{{\boldsymbol{v}}}}}}}({{{{{{\boldsymbol{r}}}}}}}_{j})|{r}_{{ij}}=r\rangle }_{i\ne j}$ (see the “Methods” section). At short distances, we find positive correlations, indicating that nearby cells move in a similar direction, even beyond the global rotation (Fig. 2a). Pronounced positive correlations also appear at large distances up to the spheroid diameter, indicating that cells on different sides of the spheroids tend to fluctuate in the same 3D directions. We find that a correlation length ${l}_{{{{{{\rm{corr}}}}}}}$ defined by $\widetilde{C}\left({l}_{{{{{{\rm{corr}}}}}}}\right)=0,$ scales with the system size (inset of Fig. 2a) and observe an approximate collapse of $\widetilde{C}(r)$ upon rescaling the intercellular distance $r$ by the spheroid radius $R$ (Fig. 2a, Supplementary Fig. 12a, b). In summary, the correlations of velocity fluctuations reveal a collective migration mode in these spheroids with a dominant length scale set by their size.

To investigate the nature of this collective mode, we analyze the spatiotemporal structure of the velocity fluctuation field of cells in the equatorial plane (Fig. 2b). Two distinct regions emerge in almost every snapshot: one with cell velocities fluctuating in the direction of the global rotation, and another, on the opposite side of the spheroids, where cell velocities fluctuate against the rotation direction (Fig. 2c). The azimuthal component of velocity fluctuations measured along the equator exhibits an approximately sinusoidal profile, with a wavelength equal to the spheroid perimeter (Fig. 2f, Supplementary Section 1.4). Thus, instantaneously, cells on one side of the spheroid perform faster rotational motion than cells on the opposite side. This fluctuation pattern propagates along the equator (Fig. 2d, e), and hence we identify it as a velocity wave. The speed of wave propagation is approximately equal to that of the global rotation (Fig. 2g, Supplementary Section 1.5). These results thus demonstrate that spheroids exhibit a velocity wave, which propagates along the equator of the global rotation with a wavelength equal to the spheroid perimeter.

### Supracellular flow patterns on the spheroid surface

**Fig. 3:**
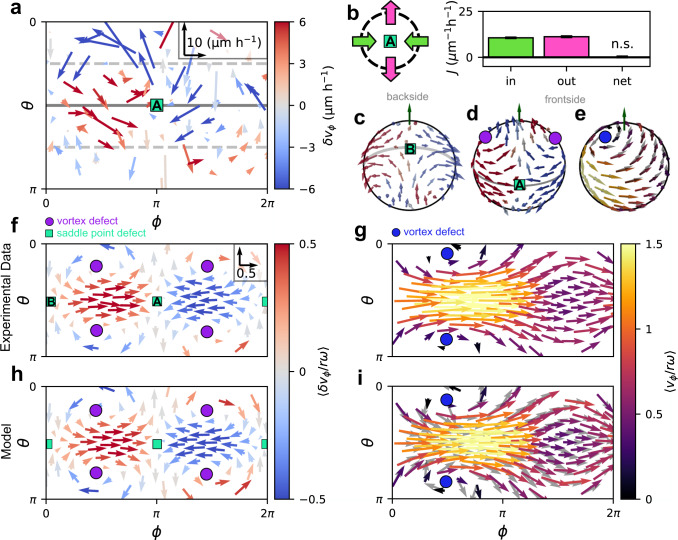
Supracellular flow patterns on the spheroid surface. **a** Snapshot of the tangential velocity fluctuation field, $\delta {{{{{{\boldsymbol{v}}}}}}}^{{{{{{\rm{t}}}}}}},$ in the surface layer of an individual spheroid represented in spherical coordinates $\phi$ and $\theta$ (see the “Methods” section). Inset: vector scale. **b** Flux analysis around the saddle point ahead of the velocity wave maximum (see the “Methods” section). Bar plot shows the absolute values of the average influx (mostly along the equator) and outflux (mostly towards the poles). Black bar indicates the difference between influx and outflux, which is not significantly different from 0 according to a two-sided *t*-test ($p=0.9$). **c**, **d** Backside (**c**) and frontside (**d**) of the average velocity fluctuation field of the experimental spheroids. Both are shown from the same perspective, with the backside field shown by looking through the sphere. **e** Frontside of the average total velocity field in the simulation shown from the same perspective as (**d**). Gray arrows indicate the average polarity field in the simulation. **f**, **h** Tangential components of the average velocity fluctuation field $\left\langle \delta {{{{{\boldsymbol{v}}}}}}/r\omega \right\rangle$ in spherical coordinates for the experiment (**f**) and our model (**h**). Inset of (**f**) shows the vector scale for all panels (**f**–**i**). **g**, **i** Tangential components of the total velocity field $\left\langle {{{{{\boldsymbol{v}}}}}}/r\omega \right\rangle$ in spherical coordinates for the experiment (**g**) and our model (**i**). As in **c**–**h**, averages are performed over both time and different spheroid realizations (Supplementary Section 1.7). Gray vectors in (**i**) indicate the average polarity field in the simulation.

To further investigate the properties of the velocity wave, we characterize cellular flow throughout the spheroid. Importantly, we observe no significant cellular exchange between the surface and the core, as shown by the absence of significant radial cellular flows (Supplementary Section 1.6). Therefore, we analyze the motion of cells in the surface layer (Fig. 2b), which exhibits the largest velocity fluctuations that are dominated by components tangential to the spheroid surface (Fig. 2c, Supplementary Fig. 4a, c–i). Snapshots of these tangential velocity fluctuations $\delta {{{{{{\boldsymbol{v}}}}}}}^{{{{{{\rm{t}}}}}}}$ in spherical coordinates (see the “Methods” section) reveal that cell motion features significant polar components near points on the equator where the azimuthal flow either converges or diverges with correspondingly high-velocity gradients of the equatorial velocity wave (Fig. 3a). This supracellular pattern is organized by apparently incompressible tangential flow across the whole spheroid surface. We show this by measuring the tangential cell flux ${J=\rho }_{{\rm {s}}}\delta {{{{{{\boldsymbol{v}}}}}}}^{{{{{{\rm{t}}}}}}}$ in the surface layer, where ${\rho }_{{\rm {s}}}$ is the surface density of cells (see the “Methods” section). While there are significant cellular fluxes with large spatial variations, their divergences are zero within our detection limit (Supplementary Section 1.9), even around the extrema of the velocity gradients (Fig. 3b).

The supracellular pattern associated with the velocity wave is further illustrated by the average velocity fluctuation field in the frame co-moving with the wave (Fig. 3c, d, see the “Methods” section). The large-scale pattern in this field features a total of six topological defects, at which the velocity direction is undefined (Fig. 3f). This pattern is robust: it is not only visible in the population average, but also appears instantaneously in individual spheroids (Supplementary Section 1.7.2). The four vortex defects carry a topological charge of ${q}_{{{\mbox{v}}}}=+ 1$, while the charge of the two saddle point defects is ${q}_{{{\mbox{s}}}}=-1$. The topological charges add up to ${q}_{{{\mbox{total}}}}=4{q}_{{{\mbox{v}}}}+2{q}_{{{\mbox{s}}}}=2$, as dictated by the Poincaré–Hopf theorem for continuous vector fields on a sphere. In the average total velocity field of the spheroids, the defect structure manifests as a diverging and converging pattern of motion around the azimuthal position ($\phi \,\approx \,3\pi /2$) of the velocity wave minimum (Fig. 3g). This pattern describes cell motion over the poles and two vortex defects in the velocity field that are displaced away from the poles of the average rotation. Thus, our results show that, in addition to the azimuthal cell velocity at the equator, also the non-azimuthal component of the velocity field away from the equator is spatially modulated (Supplementary Fig. 5e). Altogether, the patterns in the velocity and the velocity fluctuation fields show how cells ahead of the velocity wave maximum divert towards the poles. Cells at the poles flow into the equatorial region behind the wave maximum where cells accelerate again, thereby generating a global supracellular pattern of cell motion shaped by the spherical geometry.

### Active particle model on a sphere captures velocity waves

**Fig. 4:**
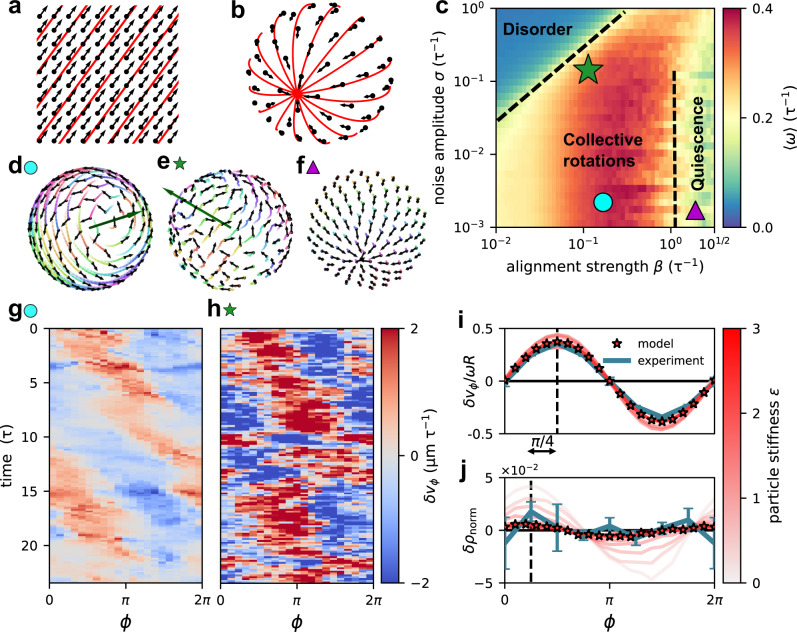
Velocity waves in a model for active particles on a sphere. **a**, **b** Ground states of an aligning vector field on a flat surface (**a**) and on a sphere (**b**). Both are found by implementing alignment interactions between vectors at fixed positions. Red lines show a chosen set of geodesics. On a spherical surface, no global order in a vector field is possible and different geodesics intersect. **c** Phase diagram of weakly compressible ($\epsilon=2$) active aligning particles constrained to a sphere constructed from the average angular speed ${{\langle }}\omega {{\rangle }}$. The simulation timescale $\tau$ is defined through the speed of self-propulsion: $\tau=\frac{1{{{{{\rm{\mu }}}}}}{{{{{\rm{m}}}}}}}{{v}_{0}}.$ The green star indicates the parameter value ($\beta=0.1$, $\sigma=0.4$), where the model quantitatively reproduces the rotational dynamics measured experimentally. Black dashed lines suggest apparent phase boundaries. **d–f** Snapshots of predicted states marked in the phase diagram. Black vectors are particle polarities, trajectories are plotted as colored lines fading over time (Supplementary Movies 3, 4). The green vector shows the axis of rotation of the spherical layer of active particles. **g**, **h** Representative kymograph $\delta {v}_{\phi }\left(\phi,\,t\right)$ of a velocity wave predicted by our model in the regime of low noise collective rotation ($\beta=0.32$, $\sigma {{{{{\boldsymbol{=}}}}}}0.006$) (**g**) and for the experimental parameter values (**h**). **i**, **j** Average rescaled velocity wave profile $\delta {v}_{\phi }/\omega R$ (**i**), and average normalized density fluctuations $\delta {\rho }_{{{{{{\rm{norm}}}}}}}=\frac{{\rho }_{s}-\left\langle {\rho }_{s}\right\rangle }{\left\langle {\rho }_{s}\right\rangle }$ (**j**). We show both quantities for different values of the particle stiffness $\epsilon$. Stars indicate the repulsion strength ($\epsilon=2$) used throughout this study to reproduce the experimental data. The blue lines indicate the experimental result.

To theoretically elucidate the physical implications of the spherical geometry (Fig. 4a, b) for the collective dynamics in the surface layer of rotating spheroids, we employ a minimal biophysical model for a layer of active particles constrained to a sphere^40^. This model features common aspects of collective cell migration^23^, including self-propulsion along a polarity vector, which aligns with the polarity of neighboring cells with strength $\beta$ and is subject to dynamical noise with amplitude $\sigma$ (see the “Methods” section). Here, motivated by our experimental observation of apparently incompressible flow (Fig. 3b, Supplementary Section 1.9), we focus on the less explored^46^ limit of a dense and weakly compressible spherical layer of active particles by imposing strong repulsion interactions between particles.

We vary two key parameters, $\beta$ and $\sigma$, in this model to construct a phase diagram for active multicellular motion on the sphere (Fig. 4c). For weak alignment, we find a disordered phase, exhibiting neither polarity alignment nor global rotations. When alignment interactions are strong, the system organizes into an ordered but quiescent regime with a polarity field containing two aster defects (Fig. 4f), similar to the ground state of a vector field with local alignment interactions on a sphere (Fig. 4b). In contrast, at intermediate alignment strengths, we find a regime with persistent global rotations (Fig. 4d). The collective dynamics in this phase are remarkably similar to our experimental observations (Fig. 4e). In fact, we identify parameter values for which the model quantitatively reproduces the experimental distribution of rotational order (Fig. 1i) and the correlation function of velocity fluctuation directions (Fig. 2a), including the scaling of the correlation length with spheroid size (inset of Fig. 2a, Supplementary Fig. 12).

Within the collective rotation regime, the model robustly predicts the emergence of velocity waves (Fig. 4g, h, Supplementary Section 4.1). As in our experiments, the wave predicted by the model consists of a single-wavelength velocity modulation along the equator, accompanied by four vortices (Fig. 3h, i). The two vortex defects in the polarity field are displaced away from the poles, and therefore drive a state of global rotation with polarized flows over the poles. This indicates that particles in the model actively move towards and over the poles (Fig. 3e, Supplementary Section 4.5.2). Furthermore, the model predicts that the wave propagates approximately with the same angular speed as the global rotation: ${\omega }_{{{{{{\rm{wave}}}}}}}\,\approx \,\omega$ (Supplementary Fig. 17e, f), consistent with our experiments. Finally, we find that the emergence of the velocity wave does not depend sensitively on the specific form of the alignment interactions between the self-propelled particles, or the dimensionality of the model (Supplementary Section 4.4). Specifically, we consider a 3D extension of our model with particles also in the bulk (Supplementary Movie 7) and still find velocity waves in the surface layer consistent with the experimental observation. Our results thus suggest that the velocity wave and the accompanying vortices in the rotating spheroids can be understood as a collective mode emerging from interacting active particles confined to a spherical surface.

While we do not detect signatures of compressible flow in our experiments (Fig. 3b), the spherical layer of active particles in our model can exhibit finite compressibility. To study the impact of compressibility, we vary the particle stiffness $\epsilon$, which sets the amplitude of the repulsive interaction between particles in the model. Independently of compressibility in this range, our model robustly exhibits the same velocity wave. However, in the compressible regime, the wave is accompanied along the equator by a density wave (Fig. 4i, j, Supplementary Fig. 17a, b, e)$,$ lagging the velocity wave by a phase-shift of approximately $\pi /4$ (45°). As we reduce compressiblity in the model by increasing $\epsilon$, the amplitude of the velocity wave relative to the global rotation is largely unaffected (Fig. 4i), whereas the amplitude of the density wave diminishes (Fig. 4j). At relatively high particle stiffnesses ($\epsilon \, > \,2$), density modulations predicted by the model fall below our experimental detection limit (Supplementary Fig. 13h–j) and the flow pattern becomes apparently incompressible (Supplementary Fig. 9f), as in our spheroid experiments. Thus, while the velocity wave in the experimental spheroids is embedded in an apparently incompressible cellular flow pattern, our model indicates that the velocity wave may be a more general collective mode of migration that remains robust even with significant compressibility.

### Curvature induces active velocity waves

**Fig. 5:**
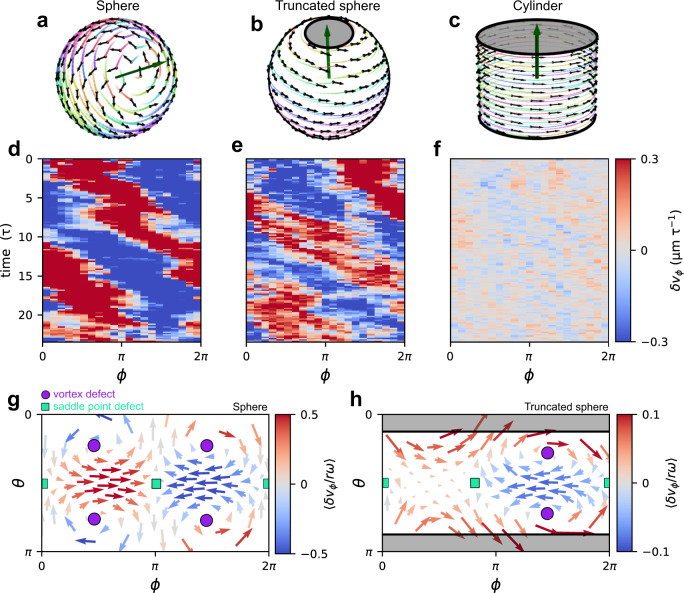
Velocity waves on different geometries. **a–c** Snapshots of the dynamics predicted by our model of active particles on a sphere in a low-noise rotation regime (**a**), on a truncated sphere with low-noise rotations (**b**), and on a cylinder (**c**). **d–f** Representative kymographs $\delta {v}_{\phi }\left(\phi,t\right)$ of the equatorial azimuthal velocity fluctuations predicted by our model for the three different geometries considered in (**a**–**c**). **g**, **h** Tangential components of the average velocity fluctuation field $\left\langle \delta {{{{{\boldsymbol{v}}}}}}/r\omega \right\rangle$ in spherical coordinates for the closed sphere in the experimental parameter regime (**g**) and of the truncated sphere in an experimentally relevant parameter regime (**h**).

Having demonstrated with a biophysical model that active velocity waves occur in a layer of active particles constrained to a sphere, we next investigate which properties of the sphere are required for their formation. To this end, we use our simulations to disentangle the role of the two key properties of the spherical geometry: positive Gaussian curvature and the topology of a spherical surface. By removing two opposing spherical caps from the sphere, we consider a *truncated sphere*, whereby we retain the positive Gaussian curvature of the sphere but change the closed spherical surface to a surface with boundaries (Fig. 5b, Supplementary Movie 5). Remarkably, however, the velocity wave still emerges, indicating its robustness to this change of the topology of the confinement (Fig. 5e, Supplementary Section 4.6.1). Importantly, for this case, the total topological defect charge ${q}_{{{\mbox{total}}}}$ must be equal to zero, as we indeed observe in our simulations (Fig. 5h). In contrast, on cylinders, which have the same topology as truncated spheres but zero Gaussian curvature (Fig. 5c, Supplementary Movie 6), we do not observe a single-wavelength velocity wave propagating in the direction of the global rotation (Fig. 5f, Supplementary Section 4.6.2). Taken together, these results demonstrate that the curvature and topology of spheroids play distinct roles in determining the collective dynamics: While the topological defect structure of the global flow pattern is constrained by the spherical topology, the active velocity waves themselves are induced by the Gaussian curvature.

## Discussion

In conclusion, we discover a collective mode of cell migration manifesting as an active velocity wave modulating the global rotations of mammary cell spheroids. This velocity wave propagates along the equator with a wavelength equal to the spheroid perimeter and is embedded in a supracellular pattern of motion that spans the entire spheroid surface. We demonstrate using an active particle model that this supracellular pattern emerges from the constraints imposed by the curved spherical geometry on an interacting active matter system. Our model indicates that the active velocity wave emerges on surfaces with Gaussian curvature and that its presence is robust against the changes in topology and compressibility that we considered. Thus, the emergence of this velocity wave in cell spheroids illustrates how curvature and topology can shape the collective behavior of curved multicellular tissues.

Wave-like instabilities are a key feature of collective behavior^47^ and give insight into the mechanical and dynamical properties of active matter systems. For example, wave-like phenomena in multicellular systems include mechanical^48^ and mechanochemical^49^ waves in spreading epithelial monolayers arising from the activity and mechanical interactions between cells. More broadly, sound waves have been theoretically predicted^50^ and experimentally^51^ observed in active matter flocks on flat surfaces. Hydrodynamic theories predict such sound waves to also manifest on spherical surfaces^41^, where hydrodynamic instability causes velocity waves to propagate in phase with density waves along the equator of the rotating flock. The modulations in the velocity field associated with these sound waves are purely azimuthal and propagate at multiple wavelengths. The active velocity wave that we report here appears to be different from the previously-reported sound waves: Our model predicts that the velocity wave is accompanied by an out-of-phase density wave, and both our model and experiments indicate that the wave is embedded in a supracellular pattern of motion characterized by non-azimuthal cell flows towards the poles. Furthermore, we observe a single wavelength equal to the spheroid perimeter. Finally, as an alternative to sound waves, the active velocity wave may be related to kinematic waves, produced by the advection of a fluctuation pattern by the background flow^52^. However, further theoretical work is needed to identify the hydrodynamic instability that gives rise to active curvature-induced velocity waves in spheroids.

The curvature-induced velocity waves may be a general collective mode of motile cellular layers in curved tissue. Thus, other spherical cellular assemblies, such as egg chambers^4^ and intestinal^9^, pancreatic^10^, and brain organoids^11^, but also a broader range of curved epithelial sheets, including in vitro assays^31^, or in vivo in intestinal villi^53^ could exhibit similar wave phenomena. The topology of spheroids dictates the existence of topological defects, manifesting as four vortex defects and two saddle point defects in the flow field, where equatorial cell fluxes are balanced by fluxes towards the poles of the spheroid. Such a defect structure is similar to recent observations of incompressible flow in fly embryos^54^. Moreover, cell fluxes near topological defects were observed to drive layer formation in bacterial colonies^55^, and they are related to mechanical stresses and sites of cell death in epithelial monolayers^56^. While it is currently unclear whether the velocity waves in spheroids serve a biological function, their presence in curved cell migration may have been identified by evolution as a strategy to generate stresses onto the surrounding extracellular matrix^20^. For example, these stresses could enable cells to invade the extracellular environment, for instance during cancer metastasis^5^. Finally, the generality of our model suggests that the patterns we discover in multicellular spheroids could be relevant for a broader range of curved active systems like spherical actin layers^57^ and schools of fish^58^.

## Methods

### Mammary cell spheroid culture and immunofluorescence staining

MCF10A cells were purchased from ATCC and cultured in a DMEM/F12 medium (Invitrogen, 11965-118) supplemented with 20 ng/ml epidermal growth factor (Peprotech, AF-100-15), 5% horse serum (Invitrogen, 16050-122), 100 ng/ml cholera toxin (Sigma, C-8052), 0.5 μg/ml hydrocortisone (Sigma, H-0888), 10 μg/ml insulin (Sigma, I-1882) and 1% penicillin and streptomycin (Thermo Fisher, 15140122). To prepare the mammary spheroid, MCF10A cells were harvested using a 0.05% trypsin solution (Thermo Fisher, 25300054) and seeded in a collagen network (3.6 mg/ml, FibriCol®, Catalog #5133) supplied with Matrigel (2.0 mg/ml, Corning, 354234). The initial cell density is low (10^4^/ml) to avoid interaction between spheroids. To visualize the cell nuclei, the MCF10A cells were transfected with GFP-NLS using lentivirus (Essen Bioscience, 4475). The three-dimensional motion of the cells within the mammary spheroid was recorded using confocal microscopy (Leica, TCL SP8). The temperature, CO_2_ level, and humidity were well-controlled using a culture box during the imaging process. The cell nuclei were further tracked from the video with Trackmate (v5.2.0, Fiji)^59^.

### Analysis of trajectories

All 3D nucleus positions are transformed into the center of mass (COM) frame of the spheroids. Translational motion of the spheroids is uncorrelated and small compared to the spheroid radii (Supplementary Section 1.1). We write ${{{{{{\boldsymbol{r}}}}}}}_{i}$ for the positions of the cell nuclei in the COM frame. Cell velocities are estimated by numerical derivates of the cell nucleus trajectories, ${{{{{{\boldsymbol{v}}}}}}}_{i}(t)=\left({{{{{{\boldsymbol{r}}}}}}}_{i}\left(t+\varDelta t\right)-{{{{{{\boldsymbol{r}}}}}}}_{i}\left(t\right)\right)/\varDelta t$, where $\varDelta t=10\,{{\min }}$ is the observation interval. To characterize rotations, we infer the rotation matrix ${{{{{\mathcal{R}}}}}}\left(t\right)$ of each spheroid at each point in time, which describes the average rotation of all cells in the spheroid between subsequent frames. We infer this matrix by simultaneously minimizing the cost functions ${{E}_{k}}^{2}={\sum }_{i=0}^{N}{\left({{{{{{{\boldsymbol{r}}}}}}}_{i}^{{{{{{\boldsymbol{k}}}}}}}}\left(t+\Delta t\right)-{{\widetilde{{{{{{\boldsymbol{r}}}}}}}}_{i}^{{{{{{\boldsymbol{k}}}}}}}}\left(t\right)\right)}^{2}$, where ${\widetilde{{{{{{\boldsymbol{r}}}}}}}}_{i}\left(t\right)\,={{{{{\mathcal{R}}}}}}\left(t\right){{{{{{\boldsymbol{r}}}}}}}_{i}\left(t\right)$ and $k={{{{\mathrm{1,2,3}}}}}$ indicates the three spatial dimensions^60^. The angular velocity ${{{{{\boldsymbol{\omega }}}}}}\left(t\right)$ is then found from ${{{{{\mathcal{R}}}}}}\left(t\right)$ via a python implementation (OpenCv) of the Rodrigues’ rotation formular^61^ that solves ${{{{{\mathcal{R}}}}}}\left(t\right){{{{{\boldsymbol{\omega }}}}}}\left(t\right)$ = ${{{{{\boldsymbol{\omega }}}}}}\left(t\right)$. The angular speed is $\omega (t)={|}{{{{{\boldsymbol{\omega }}}}}}\left(t\right)|$, and the axis of rotation is $\hat{{{{{{\boldsymbol{\omega }}}}}}}\left(t\right)\,=\,{{{{{\boldsymbol{\omega }}}}}}\left(t\right)/\omega \left(t\right)$. This axis of rotation is commonly stable, but undergoes random reorientations (Supplementary Section 1.3.2). Furthermore, we compute the rotational order parameter by^29^:

1 $$\varOmega \left(t\right)=\frac{1}{N}\left | {\sum }_{i=0}^{N}\frac{{{{{{{{\boldsymbol{r}}}}}}}_{i}^{{{\perp }}}}\left(t\right)\times {{{{{{\boldsymbol{v}}}}}}}_{i}(t)}{\left|{{{{{{{\boldsymbol{r}}}}}}}_{i}^{{{\perp }}}}\left(t\right)\times {{{{{{\boldsymbol{v}}}}}}}_{i}(t)\right|}\cdot \hat{{{{{{\boldsymbol{\omega }}}}}}}\left(t\right)\right | $$

where ${{{{{{{\boldsymbol{r}}}}}}}_{i}^{{{\perp }}}}={{{{{{\boldsymbol{r}}}}}}}_{i}-\left({{{{{{\boldsymbol{r}}}}}}}_{i}\cdot \hat{{{{{{\boldsymbol{\omega }}}}}}}\right){{{{{{\boldsymbol{r}}}}}}}_{i}$ is the orthogonal component of the positions of the cells with respect to the axis of rotation $\hat{{{{{{\boldsymbol{\omega }}}}}}}\left(t\right)$ and $N$ is the number of cells. We determine the velocity fluctuations of cells around the global rotation using $\delta {{{{{{\boldsymbol{v}}}}}}}_{i}=\,\left({{{{{{\boldsymbol{r}}}}}}}_{i}\left(t+\Delta t\right)-{{{{{\mathcal{R}}}}}}\left(t\right){{{{{{\boldsymbol{r}}}}}}}_{i}\left(t\right)\right)/\Delta t$. The cross-correlation function of the velocity- and velocity fluctuation directions are given by^29^

2 $$C\left(r\right)=\,\frac{{\sum }_{i\ne j}^{N}\delta ({{{{{\rm{|}}}}}}{{{{{{\boldsymbol{r}}}}}}}_{{ij}}{{{{{\rm{|}}}}}}-r){\hat{{{{{{\boldsymbol{v}}}}}}}}_{i}{\hat{{{{{{\boldsymbol{v}}}}}}}}_{j}}{{\sum }_{i\ne j}^{N}\delta ({{{{{\rm{|}}}}}}{{{{{{\boldsymbol{r}}}}}}}_{{ij}}{{{{{\rm{|}}}}}}-r)},\,\widetilde{C}\left(r\right)=\,\frac{{\sum }_{i\ne j}^{N}\delta ({{{{{\rm{|}}}}}}{{{{{{\boldsymbol{r}}}}}}}_{{ij}}{{{{{\rm{|}}}}}}-r){\delta \hat{{{{{{\boldsymbol{v}}}}}}}}_{i}\delta {\hat{{{{{{\boldsymbol{v}}}}}}}}_{j}}{{\sum }_{i\ne j}^{N}\delta ({{{{{\rm{|}}}}}}{{{{{{\boldsymbol{r}}}}}}}_{{ij}}{{{{{\rm{|}}}}}}-r)}$$

where ${\hat{{{{{{\boldsymbol{v}}}}}}}}_{i}={{{{{{\boldsymbol{v}}}}}}}_{i}/\left|{{{{{{\boldsymbol{v}}}}}}}_{i}\right|$ and ${\delta \hat{{{{{{\boldsymbol{v}}}}}}}}_{i}=\delta {{{{{{\boldsymbol{v}}}}}}}_{i}/\left|\delta {{{{{{\boldsymbol{v}}}}}}}_{i}\right|$ and ${{{{{{\boldsymbol{r}}}}}}}_{{ij}}={{{{{{\boldsymbol{r}}}}}}}_{i}-{{{{{{\boldsymbol{r}}}}}}}_{j}$ is the cell distance in 3D space. We approximate the Dirac-delta function $\delta (|{{{{{{\boldsymbol{r}}}}}}}_{{ij}}|-r)$ by discrete binning of the intercellular distances. Note that in the main text we rescale the intercellular distance by the spheroid radius. For more details see Supplementary Section 1.3.1.

### Analysis of vector fields

For the sake of simplicity, we call the set of all velocities $\{{{{{{{\boldsymbol{v}}}}}}}_{i}\left(t\right)\}$ at their respective positions ${{{{{{\boldsymbol{r}}}}}}}_{{{{{{\boldsymbol{i}}}}}}}$ the velocity field ${{{{{\boldsymbol{v}}}}}}\left({{{{{\boldsymbol{r}}}}}},t\right)$ of the spheroids at time $t$. Similarly, we call $\{\delta {{{{{{\boldsymbol{v}}}}}}}_{i}\left(t\right)\}$ the velocity fluctuation field $\delta {{{{{\boldsymbol{v}}}}}}\left({{{{{\boldsymbol{r}}}}}},t\right)$. Later on, we explicitly coarse-grain to find ${{{{{\boldsymbol{v}}}}}}\left({{{{{\boldsymbol{r}}}}}},t\right)$ and $\delta {{{{{\boldsymbol{v}}}}}}\left({{{{{\boldsymbol{r}}}}}},t\right).$ We rotate the velocity- and the velocity fluctuation field such that the instantaneous axis of rotation describing the rotational motion patterns is aligned to the *z*-axis of a new coordinate frame ${O}^{{\prime} }$. We can formulate this as a linear transformation ${{{{{\boldsymbol{\tau}}}}}}\left(t\right)$ equivalent to a rotation of the vector fields: ${{{{{{\boldsymbol{r}}}}}}}^{{\prime} }={{{{{\mathcal{T}}}}}}\left(t\right){{{{{\boldsymbol{r}}}}}},\,{{{{{{\boldsymbol{v}}}}}}}^{{\prime} }={{{{{\mathcal{T}}}}}}\left(t\right){{{{{\boldsymbol{v}}}}}}$, $\delta {{{{{{\boldsymbol{v}}}}}}}^{{\prime} }={{{{{\mathcal{T}}}}}}\left(t\right)\delta {{{{{\boldsymbol{v}}}}}}$*,* where ${{{{{\mathcal{T}}}}}}\left(t\right)$ is constructed from the axis of rotation (Supplementary Section 1.3.3). Henceforth, for simplicity we omit the primes in the coordinates of the rotated frame. We represent the velocity- and the velocity fluctuation field by the usual spherical coordinates $\left({\phi }_{i},\,{\theta }_{i},\,{r}_{i}\right)$ for the positions of the *i*th cell. The angle $\phi \in \left[{{{{\mathrm{0,2}}}}}\pi \right]$ parameterizes the azimuthal angle and the angle $\theta \in \left[0,\pi \right]$ parameterizes the polar angle. We define tangential velocity fluctuations of the *i*th cell as $\delta {{{{{{\boldsymbol{v}}}}}}}^{t}({{{{{{\boldsymbol{r}}}}}}}_{i},t)=\delta {{{{{\boldsymbol{v}}}}}}({{{{{{\boldsymbol{r}}}}}}}_{i},t)-[{\hat{{{\boldsymbol{e}}}}}_{{{{{{{\boldsymbol{r}}}}}}}_{i}}\cdot \delta {{{{{\boldsymbol{v}}}}}}({{{{{{\boldsymbol{r}}}}}}}_{i},t)]{\hat{{{\boldsymbol{e}}}}}_{{{{{{{\boldsymbol{r}}}}}}}_{i}}=[{\hat{{{\boldsymbol{e}}}}}_{{\phi }_{i}}\cdot \delta {{{{{\boldsymbol{v}}}}}}({{{{{{\boldsymbol{r}}}}}}}_{i},t)]{\hat{{{\boldsymbol{e}}}}}_{{\phi }_{i}}+[{\hat{{{\boldsymbol{e}}}}}_{{\theta }_{i}}\cdot \delta {{{{{\boldsymbol{v}}}}}}({{{{{{\boldsymbol{r}}}}}}}_{i},t)]{\hat{{{\boldsymbol{e}}}}}_{{\theta }_{i}}$and similarly the tangential velocity field ${{{{{{\boldsymbol{v}}}}}}}^{{{{{{\rm{t}}}}}}}({{\boldsymbol{r}}}_{i},t)=[{\hat{{{\boldsymbol{e}}}}}_{{\phi }_{i}}\cdot {{{{{\boldsymbol{v}}}}}}({{\boldsymbol{r}}}_{i},t)]{\hat{{{\boldsymbol{e}}}}}_{{\phi }_{i}}+ [{\hat{{{\boldsymbol{e}}}}}_{{\theta }_{i}}\cdot {{{{{\boldsymbol{v}}}}}}{{{(}}}{{{{{{\boldsymbol{r}}}}}}}_{i},t{{{)}}}]{\hat{{{\boldsymbol{e}}}}}_{{\theta }_{i}}\,.$ We show the $\phi$- and the $\theta$- components of the velocity fluctuations as a vector field in Fig. 3a. When we employ this representation, we restrict the velocity fields to the surface layer defined by $\left|{{{{{\boldsymbol{r}}}}}}\right|\, > \,{r}_{{{{{{\rm{thresh}}}}}}}=0.8R$ where *R* is the radius of the spheroids. The spheroid radius *R* is determined by creating a convex hull around the positions of the cells and averaging the distance of the outermost cells to the COM (Supplementary Section 1.2). The pattern we observe in this representation is not sensitive to the choice of ${r}_{{{{{{\rm{thresh}}}}}}}$.

### Kymographs of velocity waves and density fluctuations

We find the kymograph of the equatorial azimuthal fluctuation profile in the surface layer of the spheroids by defining ${N}_{{\rm {b}}}$ bins along the azimuthal $\phi$-direction with width ${\rm {d}}\phi$ . We furthermore focus on the equatorial region defined by $\theta \in \left[\pi /2-{\rm {d}}\theta,\pi /2+{\rm {d}}\theta \right]$ . We choose ${\rm {d}}\theta=\pi /4,\,{N}_{{{{{{\rm{b}}}}}}}=10$ and ${\rm {d}}\phi={{{{{\rm{\pi }}}}}}/5,$ and focus on the surface layer defined by $\left|{{{{{\boldsymbol{r}}}}}}\right|\, > \,{r}_{{{{{{\rm{thresh}}}}}}}=0.6R$. The $\phi$-component of the velocity fluctuations at time $t:$ ${\hat{{{\boldsymbol{e}}}}}_{{\phi }_{{{{{{\rm{i}}}}}}}}\delta {{{{{\boldsymbol{v}}}}}}({{{{{{\boldsymbol{r}}}}}}}_{i},t)$ is averaged inside these bins to obtain the kymograph $\delta {v}_{\phi }\left(\phi,t\right)$. The resulting kymograph is insensitive to the choice of ${{{{{{\rm{r}}}}}}}_{{{{{{\rm{thresh}}}}}}}$ (Supplementary Fig. 2g). Furthermore, we consider the normalized equatorial density fluctuations defined by $\delta {\rho }_{{{{{{\rm{norm}}}}}}}=\,\frac{{\rho }_{s}-\left\langle {\rho }_{s}\right\rangle \,}{\left\langle {\rho }_{s}\right\rangle }$, where ${\rho }_{s}$ is the surface density found from counting the number of cells inside the ${N}_{b}$ bins along the azimuthal $\phi$-direction with width ${\rm {d}}\phi$ as for the velocity wave. $\left\langle {\rho }_{s}\right\rangle$ is the average surface density in the equator found by averaging over $\phi$. For more details refer to Supplementary Sections 1.4 and 1.8.

### Averaging of vector fields

The average velocity- and velocity fluctuation field in the frame of reference of the propagating velocity wave are found by first rotating ${{{{{\boldsymbol{v}}}}}}\left({{{{{\boldsymbol{r}}}}}},t\right)$ and $\delta {{{{{\boldsymbol{v}}}}}}\left({{{{{\boldsymbol{r}}}}}},t\right)$ around the axis of rotation $\hat{{{{{{\boldsymbol{\omega }}}}}}}\left(t\right)$ so that all wave maxima are aligned on the azimuthal angle $\phi \,=\,\pi /2$. The angle by which we rotate is found through fitting a single-wavelength sinusoidal profile to the individual equatorial fluctuation profiles shown in the kymograph (Fig. 2d,e). Then the velocity and velocity fluctuation field in the surface layer are scaled down for each individual cell by $\omega \left(t\right)\left|{{{{{{\boldsymbol{r}}}}}}}_{i}\right|$ to make them dimensionless. This yields ${{{{{\boldsymbol{v}}}}}}\left({{{{{{\boldsymbol{r}}}}}}}_{i},t\right)/\omega \left(t\right)\left|{{{{{{\boldsymbol{r}}}}}}}_{i}\right|$ and $\delta {{{{{\boldsymbol{v}}}}}}\left({{{{{{\boldsymbol{r}}}}}}}_{i},t\right)/\omega \left(t\right)\left|{{{{{{\boldsymbol{r}}}}}}}_{i}\right|$. Finally, the surface layer of the spheroids is covered in ${N}_{{{{{{\rm{bins}}}}}}}\,=\,120$ uniformly distributed spherical bins. The size of each bin is defined by an angle ${\rm {d}}\varOmega \,=\,0.2$ between the position vector of each bin and the position vector of a cell. This results in sufficient covering of the surface layer with bins. All rescaled velocities and velocity fluctuations inside one bin are averaged over time and/or different experimental realizations. Finally, we find the tangential components of the average vector fields by employing the same procedure as for the individual snapshots. For more details see Supplementary Section 1.7.

### Flux analysis

We find the tangential cell flux ${\rho }_{s}\delta {{{{{{\boldsymbol{v}}}}}}}^{{{{{{\rm{t}}}}}}}$ where ${\rho }_{s}$ is the approximated surface density of cells in the surface layer. We then construct a circle around the position ${{{{{{\boldsymbol{r}}}}}}}_{0}$ to probe the incompressibility of the cell flow at this position. The size of the circle is characterized by $\Omega$ which is the angle between the positions of the cells ${{{{{{\boldsymbol{r}}}}}}}_{i}$ and ${{{{{{\boldsymbol{r}}}}}}}_{0}$. We find the cell flux through this circle by considering all cells whose positions is within $[\varTheta \,-\,{\rm {d}}\varTheta,\,\varTheta+{\rm {d}}\varTheta ]$. We project the cell flux on the normal vectors to this circle and sum all positive values and all negative values to find the out- and the influx. The sum of all projections is equal to the net cell flux. For more details see Supplementary Section 1.9.

### Active particles on curved surfaces

Based on previous work^62–65^, we model cells as active particles. We describe the motility of cells by self-propulsion with speed ${v}_{0}$ in the direction of an internal polarization defined by the polarity vector ${{{{{\boldsymbol{p}}}}}}$. We impose an alignment interaction with strength $\beta$ between polarities of neighboring cells and polarities are subject to Gaussian white noise with amplitude $\sigma$. In addition, cells repel each other by a soft repulsion potential with stiffness $\varepsilon$. On a flat surface the model is written as:

3 $${{{{{{\boldsymbol{v}}}}}}}_{i}={v}_{0}{{{{{{\boldsymbol{p}}}}}}}_{i}+{{{{{{\boldsymbol{F}}}}}}}_{{{{{{\rm{rep}}}}}}},$$

4 $${{{{{{\boldsymbol{F}}}}}}}_{{{{{{\rm{rep}}}}}}}=-\varepsilon \left(2\lambda -\,{r}_{{ij}}\right)\frac{{{{{{{\boldsymbol{r}}}}}}}_{{ij}}}{{r}_{{ij}}},$$

5 $${{{{{{\boldsymbol{p}}}}}}}_{i}={\left({{\cos }}\left({\phi }_{i}\right),{{\sin }}\left({\phi }_{i}\right)\right)}^{{\rm {T}}},$$

6 $$\frac{{\rm {d}}{\phi }_{i}}{{{\rm {d}}t}}=-\beta \mathop{\sum}\limits_{j}{{\sin }}\left({\phi }_{i}-{\phi }_{j}\right)+{\eta }_{i}\left(t\right),$$

7 $$\left\langle {\eta }_{i}\left(t\right){\eta }_{j}\left({t}^{{\prime} }\right)\right\rangle={\sigma }^{2}{\delta }_{{ij}}\delta \left(t-{t}^{{\prime} }\right)$$

where $\lambda$ is the radius of the particles and ${\sum }_{j}$ is the sum over all neighbors of the *i*th particle within a radius of interaction ${r}_{{{{{{\rm{inter}}}}}}}=2.5\lambda$. The exact choice of ${r}_{{{{{{\rm{inter}}}}}}}$ does not matter as long as the number of nearest neighbors remains close to 6 corresponding to a close packing of particles in 2D. To model the dynamics of the surface layer of the spheroids, we study this model on a surface of a sphere^40^. Importantly, we consider the less explored weakly compressible parameter regime of this model by setting the repulsion stiffness $\varepsilon$ to a sufficiently high value such that the layer of cells covers the sphere and density fluctuations are very small. We set the time scale of our simulation to $\tau=l/{v}_{0}$, where $l=1{{{{{\rm{\mu }}}}}}{{{{{\rm{m}}}}}}$ is the length scale of our simulation. We set the average surface density of cells on a sphere in our simulation to the average surface density of the cells in the surface layer of the spheroids. Together with the number of cells, this also sets the size of the sphere and the radius of cells. We numerically solve this model on a sphere by an algorithm based on the Euler-Maruyama method^40^ (Supplementary Section 3). Furthermore, we vary the underlying geometry from a sphere to a truncated sphere and a cylinder, which are both unexplored for this model. For the truncated sphere, we introduce soft boundaries at two specific latitudes. These boundaries keep the cells from moving over the poles. We make sure that the effectively excluded regions are large enough so that there are no nearest-neighbor interactions between cells on opposite sides of the excluded regions (Supplementary Section 4.6.1). For the cylinder, we use cylindrical coordinates to constrain the dynamics. This asymmetric geometry requires adjustment of how alignment interactions are implemented. For more details refer to Supplementary Section 4.6.2.

### Statistical analysis

Throughout, error bars represent the error of the mean found through bootstrapping of the underlying data set. For the average normalized density fluctuations in the frame of reference of the velocity wave, we propagate errors according to

8 $${\sigma }_{\delta {\rho }_{{{\rm {norm}}}}}={\delta \rho }_{{{\rm {norm}}}}\sqrt{{\left(\frac{{\sigma }_{\delta \rho }}{\delta \rho }\right)}^{2}+{\left(\frac{{\sigma }_{{{\langle }}\delta \rho {{\rangle }}}}{{{\langle }}\delta \rho {{\rangle }}}\right)}^{2}}$$

where ${\sigma }_{\delta \rho }=\sqrt{{\sigma }_{{\rho }_{{\rm {S}}}}^{2}+{\sigma }_{\left\langle \delta \rho \right\rangle }^{2}}$ is the error of the density fluctuations. Here ${\sigma }_{{\rho }_{{\rm {s}}}}$ is the error of the surface density and ${\sigma }_{\langle \delta \rho \rangle }$ is the error of the average density. We perform an F-test to assess the statistical significance of a sinusoidal profile in the density and velocity fluctuation field. We consider two models $\delta {\rho }_{2}\left(\phi \right)=A\,{{{{{\rm{si}}}}}}{{{{{\rm{n}}}}}}(\phi -\,{\phi }_{0})$ (“model 2”) and a uniform profile $\delta {\rho }_{1}\left(\phi \right)=0$ (“model 1”). We compute the F-value according to

9 $$F=\,\frac{({{\rm {RS}{S}}}_{1}-{{\rm {RS}{S}}}_{2})/({f}_{1}-{f}_{2})}{{{\rm {RS}{S}}}_{2}/{f}_{2}}$$

where ${{\rm {RS}{S}}}_{1/2}$ refers to the residual sum of squares of the fit with the two models $\delta {\rho }_{1/2}\left(\phi \right)$: ${{\rm {RS}{S}}}_{1/2}={{\sum }_{i}\left(\delta {\rho }_{1/2}\left({\phi }_{i}\right)-\delta {\rho }_{{{\rm {nor}m}}}\left({\phi }_{i}\right)\right)}^{2}$, and ${\phi }_{i}$ refers to the *i*th bin in our discrete approximation of $\delta {\rho }_{{{\rm {norm}}}}\left(\phi \right).$ Furthermore, ${f}_{1/2}$ is the degree of freedom of the fit. From these F-values, we find *p*-values assuming that *F* is distributed according to an F-distribution under the null hypothesis that a sinusoidal wave profile does not significantly better fit the data than a uniform profile. Finally, we perform a two-sided *t*-test to determine the statistical significance of density fluctuations and divergences in the cell flux. For more details refer to Supplementary Sections 1.4 and 1.8.

### Reporting summary

Further information on research design is available in the Nature Portfolio Reporting Summary linked to this article.

## Supplementary information

Supplementary Information

Description of Additional Supplementary Files

Supplementary Movie 1

Supplementary Movie 2

Supplementary Movie 3

Supplementary Movie 4

Supplementary Movie 5

Supplementary Movie 6

Supplementary Movie 7

Reporting Summary

## Data Availability

Experimental and simulation data is available from the corresponding author upon request.

## Code availability

Python code to analyze data and perform numerical simulations is available from the corresponding author upon request.
