# Supplementary material for: Curvature induces active velocity waves in rotating spherical tissues
Source: Nat Commun. 2023 Mar 24;14:1643. doi: 10.1038/s41467-023-37054-2 (PMC10039078; doi:10.1038/s41467-023-37054-2)
Supplement: Supplementary file 1 — Supplementary Information [file 41467_2023_37054_MOESM1_ESM.pdf]

Supplementary Information:

Curvature induces active velocity waves in rotating spherical tissues

Tom Brandstätter*, David B. Brückner*, Yu Long Han, Ricard Alert, Ming Guo, Chase P. Broedersz†

* These authors contributed equally to this work: Tom Brandstätter, David B. Brückner
† Corresponding author: c.p.broedersz@vu.nl, Phone: +31 20 59 82953

Contents

1 Supplementary experimental analysis **9**

1.1 Center of mass motion of cell spheroids . . . 9
1.2 Size, shape and number of cells of spheroids . . . 9
1.3 Supplementary analysis of velocity fields . . . 11
1.3.1 Correlation function scales with system size . . . 12
1.3.2 Dynamics of the axis of rotation . . . 12
1.3.3 Changing perspective onto velocity fields . . . 13
1.4 Assessing the significance of the velocity wave . . . 13
1.5 Wave propagation . . . 15
1.6 Dynamics in the surface layer of spheroids . . . 16
1.6.1 Importance of the surface layer for the collective dynamics . . . 17
1.6.2 Radial components of the dynamics . . . 17
1.7 Averaging velocity fields in the frame of reference of the velocity wave . . . 17
1.7.1 General procedure . . . 18
1.7.2 Robustness of the average velocity fluctuation field . . . 19
1.8 Analysis of density modulations . . . 21
1.8.1 Density fluctuations at instantaneous time points . . . 21
1.8.2 Average density modulations in the co-moving frame . . . 22
1.9 Analysis of cell fluxes . . . 23

2 Stochastic rigid-body rotation **27**

3 Model Implementation **29**

4 Supplementary model results **30**

4.1 Robustness of velocity waves and parameter overview . . . 30
4.2 Size dependence of collective dynamics . . . 32
4.3 Analysis of density modulations . . . 33
4.3.1 Density fluctuations at instantaneous time points . . . 33
4.3.2 Average density modulations in the co-moving frame . . . 33
4.4 Testing alternative models . . . 34
4.4.1 Different alignment interactions . . . 34
4.4.2 Varying the dimensionality of our model . . . 36
4.5 Analysis of rotational dynamics of the model and wave propagation . . . 39
4.5.1 Wave propagation in the experimental regime . . . 39
4.5.2 Co-movement of wave, global rotation and polarity defects . . . 39
4.6 Disentangling the role of topology and curvature . . . 40
4.6.1 Active particles on a truncated sphere . . . 41
4.6.2 Active particles on cylinders . . . 42

1 Supplementary experimental analysis

1.1 Center of mass motion of cell spheroids

In the main text, we analyze the rotational mode of the system in the center of mass (COM) frame. Here we characterize the motion of the COM. To this end, we find the trajectory of the COM in the lab frame by:

$$\vec{r}_{\mathrm{COM}}(t) = \frac{1}{N_{\mathrm{cells}}} \sum_{i=1}^{N_{\mathrm{cells}}} \vec{r}_i(t) \tag{S1}$$

Here, $\{\vec{r}_i(t)\}$ is the set of all (lab frame) positions of the cells in one spheroid and N_{cells} denotes the number of cells. Furthermore, we quantify the velocity of the COM by

$$\vec{v}_{\mathrm{COM}}(t) = (\vec{r}_{\mathrm{COM}}(t+\Delta t) - \vec{r}_{\mathrm{COM}}(t))/\Delta t \tag{S2}$$

where $\Delta t = 10$min is the observation interval. We note that the instantaneous displacement of the COM $|\vec{v}_{\mathrm{COM}}(t)|\Delta t$ is small with respect to the spheroid radius R (Fig. S1a). We explain how we compute R in section 1.2. To further characterize the COM motion of spheroids, we compute the Mean squared displacement (MSD) of the COM position of the spheroids, which we here define as $MSD(\tau) = \langle |\vec{r}_{\mathrm{COM}}(t) - \vec{r}_{\mathrm{COM}}(t+\tau)|^2 \rangle_t$. Here $\langle ... \rangle_t$ denotes an average over all time points. We find that COM motion is subdiffusive. Importantly, at the end of the observation period, the average root mean squared displacement of the COMs is around $\sqrt{10}\ \mu m$ (Fig. S1b), which is small compared to the spheroid radii (Fig. S1d). Furthermore, we consider the auto-correlation function of the velocity of the COM:

$$\chi(\tau) = \langle \vec{v}_{\mathrm{COM}}(t) \vec{v}_{\mathrm{COM}}(t-\tau) \rangle_t \tag{S3}$$

This quantity does not exhibit a characteristic time scale, which would be related to directed COM motion of collectively translating spheroids (Fig. S1c). Altogether, these results indicate the absence of persistent COM motion, meaning that cell spheroids remain approximately fixed at their lab positions. Note that throughout the main text and the supplementary information, all data analysis is conducted in the COM frame. Therefore, henceforth, we write the positions of the cells in the COM frame as $\vec{r}_i(t)$.

1.2 Size, shape and number of cells of spheroids

In this subsection, we give an overview of the $N_s = 16$ spheroids and characterize their shape as well as the number of cells in these spheroids.

Size and shape of the spheroids

We compute the radius of the spheroids by the average distance of the outermost cells of a spheroid to the COM of the spheroids. These cells are determined by creating a convex hull around the position data $\{\vec{r}_i\}$ at each point in time. Spheroid radii range from around 16 μm to around 37 μm (Fig. S1d). To quantify the change in radius over time, we compute

$$\frac{R(t_{\mathrm{final}}) - R(t_0)}{R(t_0)} \tag{S4}$$

where t_0 and t_{final} are the beginning and final time points of the observation time period. This ratio shows that spheroid radii at the end of the observation do not vary by more than 9% from their initial values at

the beginning of observation. Shapes can vary over time but remain approximately spherical. We show this by considering the sphericity of the spheroids:

$$\Psi = \frac{\pi^{\frac{1}{3}}(6V)^{\frac{2}{3}}}{A} \tag{S5}$$

The sphericity is defined by the surface area of a perfect sphere with the spheroid volume V divided by the actual spheroid surface area A.

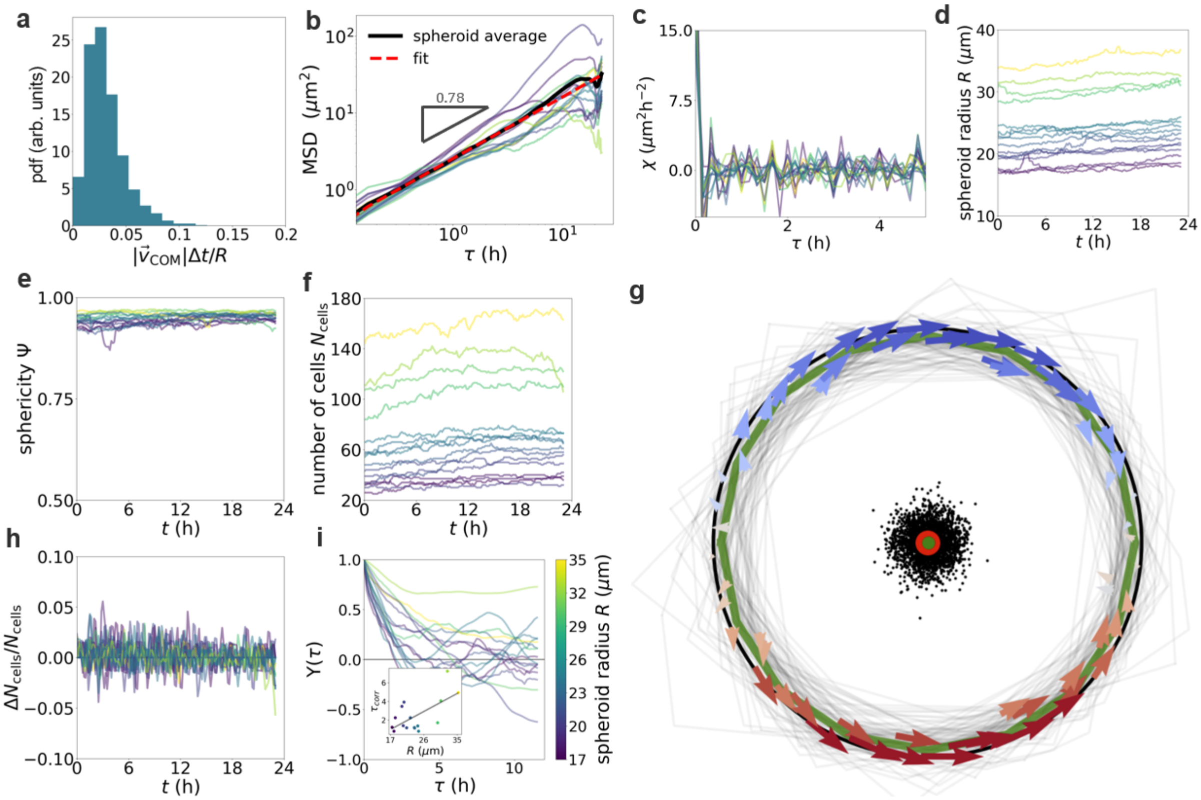

Figure S1: **COM motion, cell shape, number of particles and rotational dynamics of spheroids. a)** Distribution of the instantaneous COM displacement relative to the spheroid radius. **b)** Mean squared displacement of COM motion. Different colored lines are for individual spheroids, black line shows the average. Red dashed lines indicates the fit to the first 20 time points of the average. We fit the MSD by $C\tau^{\alpha}$ and find $C = 2.58$ and $\alpha = 0.78$. **c)** Auto correlation function of COM motion. Individual colored lines show the result of individual cell spheroids. **d)-f),h),i)** Quantities characterizing the appearance of the spheroids as functions of time for different cell spheroids. d) spheroid radius R, e) sphericity Ψ, f) number of cells N_{cells}, h) relative number fluctuations of the number of cells $\frac{\Delta N_{\text{cells}}}{N_{\text{cells}}}$, and i) auto correlation of the axis of rotation. Colorbar in (i) applies to all (b-f,h,i). **g)** Planar projection of the average velocity fluctuation field showing the velocity wave and the spheroid shape. Grey lines show a random selection of instantaneous spheroid shapes at different time points and spheroids rescaled by the spheroid radius. Shapes are found from a convex hull around the positions of cells in the equatorial region of the spheroids. Green line shows the average shape of the spheroids. Black line indicates a circle with a radius of $R = 1$, centered around zero (red dot). Black dots show the distribution of the centroids of the instantaneous shapes and green dot shows the average centroid position.

Both these quantities are found from the convex hull that we construct around the spheroid. For a perfect sphere, this quantity is equal to 1, and all other shapes have a sphericity of less than 1. We find that the sphericity fluctuates over time but remains close to 1 for all spheroids. This result indicates a robust spherical shape of the spheroids (Fig. S1e), making it possible to use spherical coordinates to represent the dynamics in the surface layer of these spheroids.

Velocity wave is not accompanied by shape fluctuations

Next, we investigate if there are potential fluctuations of the spherical shape of the spheroids that are co-moving with the velocity wave. To this end, we focus on the velocity wave in the equatorial region and determine the shape of the spheroids in this same region within the frame of reference co-moving with the velocity wave. Thus, we make a planar projection of the cell positions onto the equatorial plane and find the shape of the spheroids as a complex hull around this position data (Fig. S1g). Note that we rescale the position data by the spheroid radius to make all spheroids comparable. The shape that we find does not show systematic fluctuations away from a circle that is centered around the COM of the spheroids as shown by the average shape of the spheroids (green line in Fig. S1g). To quantify potential asymetric shape fluctuations that would move the centroid of the shape away from the COM, we find the centroid of the spheroid shape. We analyze the distribution of the centroid positions over time and different spheroids. We do not find a significant deviation of the mean of the centroid positions from the COM. Together these results show that the velocity wave in the equatorial region is not accompanied by shape fluctuations of the spheroid.

Number of cells

The number of cells N_{cells} in the spheroids ranges from around 25 to over 160 for the largest spheroid (Fig. S1f). N_{cells} is not constant in time, but exhibits a small increasing upwards trend. To further investigate this trend, we compute the rate at which the number of cells changes:

$$\nu(t) = \frac{dN_{\text{cells}}}{dt} \tag{S6}$$

We find that the average growth rate over all spheroids $\langle \nu(t) \rangle_{t,s} \approx 0.5\ \text{h}^{-1}$, where $\langle \rangle_{t,s}$ indicates an average over time t and different spheroid indices s. This positive growth rate yields a relative change of the number of cells during the measurement time $T = 23.33$ h, quantified by

$$\left\langle \frac{\langle \nu(t) \rangle_t T}{N_{\text{cells}}(t=0)} \right\rangle_s \approx 0.2 \tag{S7}$$

This indicates that the doubling time ($T_{\text{d}} \approx 5$ days) of the cell spheroids is larger than the measurement time scale ($T \approx 1$ day). Furthermore, this doubling time is much longer than the duration of a typical full rotation of a spheroid (≈ 1 day). To assess the instantaneous fluctuation of the number of cells defined by $\Delta N_{\text{cells}} = \nu(t)\Delta t$, we compute:

$$\frac{\Delta N_{\text{cells}}}{N_{\text{cells}}} = \frac{\nu(t)\Delta t}{N_{\text{cells}}(t)} \tag{S8}$$

We find that $\frac{\Delta N_{\text{cells}}}{N_{\text{cells}}}$ exhibits a narrow distribution around 0 with maximum variations of ± 0.04 (Fig. S1g). Furthermore, $\sqrt{\left\langle \left(\frac{\Delta N_{\text{cells}}}{N_{\text{cells}}}\right)^2 \right\rangle} \approx 0.01$, which indicates that fluctuations of the number of cells are very small on the time scale of the measurement interval. Altogether, these results show that cell proliferation does not make an important contribution to the dynamics of the system.

1.3 Supplementary analysis of velocity fields

In this subsection, we elaborate on how we analyze the velocity and velocity fluctuation fields of a rotating cell spheroid whose axis of rotation is not fixed in space.

1.3.1 Correlation function scales with system size

To identify patterns in the velocity field and in the velocity fluctuation field, we compute the spatial correlation function of velocity directions $\hat{v}(\vec{r}_i) = \vec{v}(\vec{r}_i)/|\vec{v}(\vec{r}_i)|$ and velocity fluctuation directions $\delta\hat{v}(\vec{r}_i) = \delta\vec{v}(\vec{r}_i)/|\delta\vec{v}(\vec{r}_i)|$. Note that $\vec{r}_i$ is the COM position of the i-th cell. Specifically, we compute for the velocity field [1] and for the velocity fluctuation field:

$$C(r) = \frac{\sum_{i\neq j}^{N} \hat{v}(\vec{r}_i)\hat{v}(\vec{r}_j)\delta(\vec{r}_{ij} = r)}{\sum_{i\neq j}^{N} \delta(\vec{r}_{ij} = r)} \tag{S9}$$

$$\tilde{C}(r) = \frac{\sum_{i\neq j}^{N} \delta\hat{v}(\vec{r}_i)\delta\hat{v}(\vec{r}_j)\delta(\vec{r}_{ij} = r)}{\sum_{i\neq j}^{N} \delta(\vec{r}_{ij} = r)} \tag{S10}$$

where we approximate the Dirac-delta function $\delta(\vec{r}_{ij} = r)$ by sharp binning in the intercellular distance $\vec{r}_{ij}$. In the main text, we claim that the correlation function of the velocity fluctuation directions approximately collapse when we rescale the intercellular distance by the spheroid radius (Main text Fig. 2a). Here we show this explicitly. The correlation functions of the velocity fluctuation directions without rescaling are shown in Fig. S12a. For these curves, we use a bin size of $dr = 5$ μm. Rescaling the intercellular distance r by the spheroid radius R results in an approximate collapse of the correlation functions (Fig. S12b). For these curves, we use a bin size of $dr = 0.1$ R where R is the radius of the spheroids. This result suggests that the collective pattern scales with the system size on the length scales considered here (The radius of the spheroids varies between 16 μm to around 37 μm). Furthermore, we find the correlation length of the velocity fluctuation field as the first zero of a polynomial fit to the not rescaled correlation functions. The correlation length as a function of spheroid size is shown in main text Fig. 2a.

1.3.2 Dynamics of the axis of rotation

In the main text methods, we describe how we infer the axis of rotation $\hat{\omega}(t)$ that describes the global rotation of the spheroids. In this subsection, we analyze the dynamics of the axis of rotation. Specifically, we compute an auto-correlation function of the axis of rotation defined by

$$\Upsilon(\tau) = \langle \hat{\omega}(t) \cdot \hat{\omega}(t-\tau) \rangle_t \tag{S11}$$

This correlation function exponentially decays with rate τ_{norm} (Fig. S1h). The average over spheroids of this decay rate is $\langle \tau_{\text{norm}} \rangle = 1.8$ h. The exponential decay indicates that the axis of rotation shows random but persistent reorientations. The decay rate τ_{norm} depends on the spheroid size: larger spheroids exhibit a more persistent axis of rotation than smaller spheroids. These results further indicate the robust presence of persistent global rotations, especially in larger spheroids.

1.3.3 Changing perspective onto velocity fields

To analyze the dynamics of cells relative to the axis of rotation of the spheroids, we construct a transformation of the velocity and velocity fluctuation field into a frame of reference whose z-axis remains aligned with the spheroid's axis of rotation at all time points. This is done by constructing a right handed coordinate frame out of $\hat{\omega}$ as $\hat{z}'$-axis and two other orthonormal vectors where we define the $\hat{y}'$-axis to be in the plane spanned by the COM frame $\hat{z}$-axis and the axis of rotation $\hat{\omega}$. Thus, positions as well as velocity and velocity fluctuation fields are projected into this new coordinate frame. We write this transformation as a linear transformation $\mathscr{T}(t)$, equivalent to a rotation: $\vec{r}'(t) = \mathscr{T}(t)\vec{r}(t)$, $\vec{v}'(t) = \mathscr{T}(t)\vec{v}(t)$, $\delta\vec{v}'(t) = \mathscr{T}(t)\delta\vec{v}(t)$. Note that in the following we drop the primes again to refer to the velocity and velocity fluctuation field in the frame of reference where the axis of rotation is aligned to a new z-axis.

1.4 Assessing the significance of the velocity wave

We observe sinusoidal velocity fluctuation profiles in the majority of the data (Fig. S2a,d). To quantify the robustness of the sinusoidal velocity fluctuation profile in the experimental data, we consider the auto-correlation function of the kymograph of equatorial velocity fluctuations:

$$\Gamma(\psi) = \langle \delta v_\phi(\phi,t)\delta v_\phi(\phi-\psi,t)\rangle_{\phi,t}$$

This auto–correlation function is sinusoidal for sinusoidal velocity fluctuation profiles and can be thus used to assess the shape of $\delta v_\phi(\phi,t)$. We find that the experimentally measured $\Gamma(\psi)$ exhibits correlation at short distance, crosses zero at $\psi \approx 0.5\pi$ and shows anti-correlation at $\psi \approx \pi$ which corresponds to the opposite side of a spheroid (Fig. S2b). This behavior is qualitatively in agreement with $\Gamma(\psi)$ for a sinusoidal profile $A(t)\sin(\phi-\phi_0)$. This shows that on average, the wave profile $\delta v_\phi(\phi,t)$ can be considered to be sinusoidal to a good approximation. The equatorial region of the cell spheroids thus exhibits a robust single-wavelength velocity wave profile with a wavelength approximately equal to the spheroid perimeter $\lambda_{\text{wave}} \approx 2\pi R$.

Throughout, we assess the statistical significance of a sinusoidal wave profile using the following F-test [2]: we test if a sinusoidal profile $\delta v_2(\phi) = A\sin(\phi-\phi_0)$ ("model 2") fits the experimentally measured wave profile $\delta v_\phi(\phi,t)$ significantly better than a uniform profile $\delta v_1(\phi) = 0$ ("model 1"). Here, model 1 is a constrained version of model 2 when setting $A = 0$. In particular, we find the F-statistics defined as:

$$F = \frac{(RSS_1 - RSS_2)/(f_1 - f_2)}{RSS_2/f_2} \tag{S12}$$

Here, $RSS_{1/2}$ refers to the residual sum of squares of the fit with model $\delta v_{1/2}$: $RSS_{1/2} = \sum_i(\delta v_{1/2}(\phi_i) - \delta v_\phi(\phi_i,t))^2$,where ϕ_i refers to the i-th bin in our discrete approximation of $\delta v_\phi(\phi_i,t)$. Furthermore, $f_{1/2}$ is the degree of freedom of the fit ($f_{1/2} = n - p_{1/2}$, where $n = 10$ is the sample size and $p_{1/2}$ is the number of parameters for model 1/model 2 ($p_1 = 0$, $p_2 = 2$)). Importantly, F is usually positive as δv_2 is a more complicated model than δv_1 and thus should better fit the data. F is directly interpretable because RSS_1 is proportional to the variance of the data (the mean of $\delta v_\phi(\phi_i)$ is 0): values of F around 0 indicate small improvement of fitting the data with model 2 relative to the variance of the data, while large values indicate a bigger improvement of fitting the data with model 2. In Fig. S2e we show the distribution of F obtained from fitting all time points throughout all spheroids with model 1 and model

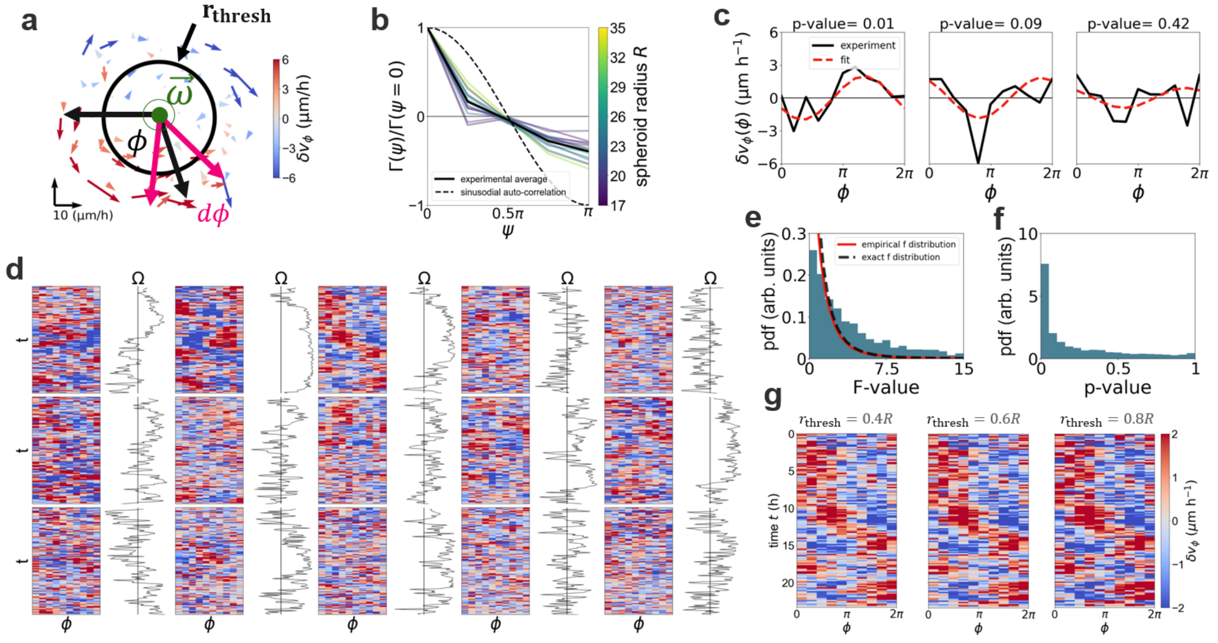

Figure S2: **Kympograph of velocity waves.** **a)** Schematic of the planar projections of the equatorial velocity fluctuation field. Pink vectors show margins of a bin with width $d\phi$. **b)** Auto correlation function $\Gamma(\psi)$ as defined in equation 1.4. Colored curves show the result for individual spheroids, black curve the experimental average and the dotted curve a sinusoidal autocorrelation. **c)** Three snapshots of $\delta v_\phi(\phi,t)$ with three different p-values. Black lines show the experimentally measured profiles, red dashed line shows the fit. **d)** Kymographs of equatorial azimuthal velocity fluctuations $\delta v_\phi(\phi,t)$ for all cell spheroids. We use $d\phi = \pi/5$ as bin size. Always to the right we show rotational order in these spheroids. The black vertical line indicates a rotational order parameter $\Omega = 0.5$. Right of the black vertical lines indicates $\Omega > 0.5$. **e)** Distribution of F-values found throughout all spheroids at all time points. Red curve shows distribution of empirical F-values, while black curve shows the exact F-distribution. The experimental distribution deviates from the F-distribution indicating that a sinusoidal profile provides a statistically significant better fit than a uniform profile. **f)** Distribution of p-values that we found from the F-values in (e) **g)** Kymograph of one spheroid for different values of r_{thresh}.

2. We find that fitting the velocity wave $\delta v_\phi(\phi,t)$ with a sinusoidal profile yields typically large (> 1) values of F (Fig. S2e), indicating that the velocity wave is typically better fitted by a sinusoidal profile than a uniform profile.

Next, we ask whether δv_2 provides a statistically significant better fit to the data than δv_1. To answer this question, we make use of the fact that F follows in theory an "F-distribution" under the Null hypothesis that model 2 does not significantly better fit the data than model 1. We test if our definition of F indeed follows this distribution by considering a scenario where we know that the Null hypothesis is true: We assume a uniform profile with added Gaussian noise with zero mean and a standard deviation similar to that of the experimental data and perform the fits as outlined above. We can show that indeed the empirical distribution of our definition of F follows approximately the exact F-distribution under the Null hypothesis (Fig. S2e). Thus, it is reasonable to extract p-values from our definition of F for the experimental data. We do this for fits at individual velocity fluctuation profiles at individual time points (Fig. S2c), giving us a distribution of p-values over time (Fig. S2f). We find that the velocity wave exhibits a distribution of p-values with a peak at small p-values and 55% of the time points exhibit a p-value smaller than 0.1. From this we conclude that our data mostly exhibits a statistically significant velocity wave. Finally, the kymographs do not depend sensitively on decreasing r_{thresh} (Fig. S2g), indicating that the velocity wave is most dominant in the outer most surface layer of the spheroids.

1.5 Wave propagation

In the main text, we state that the speed of wave propagation is approximately equal to the speed of the global rotation. Here, we show this explicitly. The speed of global rotation is quantified by the angular speed $\omega(t)$, which we infer from the data. The speed of wave propagation is characterized by $\omega_{\text{wave}}(t)$, which we here define as the angular speed of the velocity wave maximum in the 3D COM

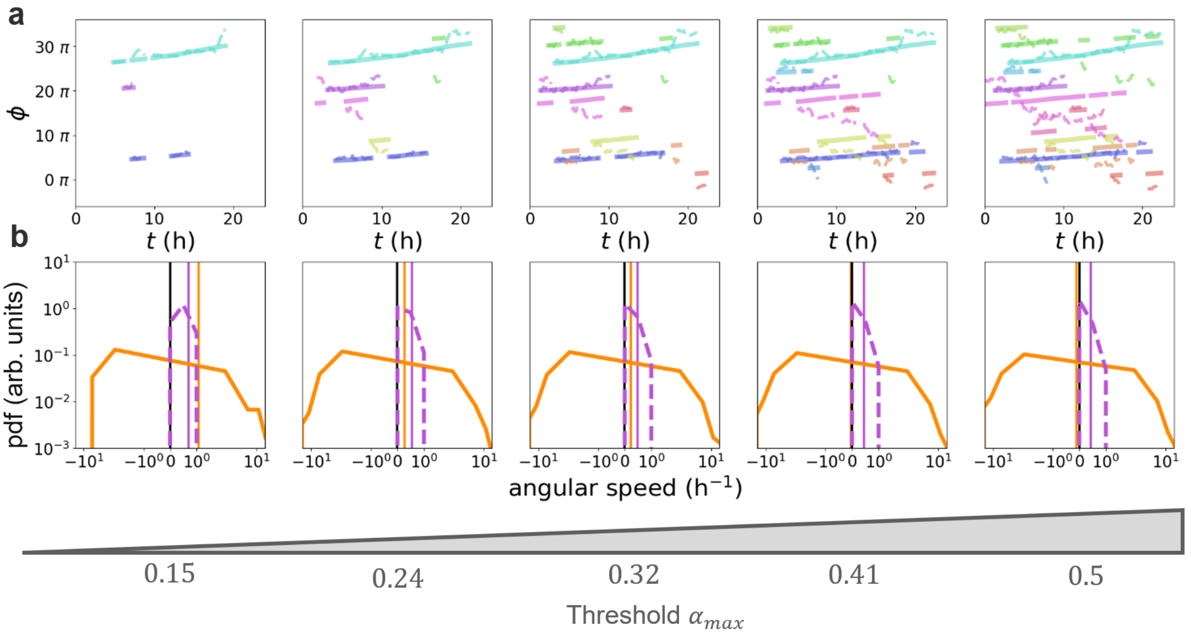

Figure S3: **Propagation of velocity wave.** **a)** For different values of α_{max}, we compare the trajectory of the global rotation (solid) and the propagating velocity wave (dashed) in all different cell spheroids. **b)** Quantitative comparison of the average angular speed of the spheroids ω (violet) and of the speed of wave propagation ω_{wave} (orange). Vertical colored lines show the average values. Black vertical line indicates zero.

frame. As $\omega(t)$ also quantifies the global rotation of the spheroids in the COM frame, $\omega_{\text{wave}}(t)$ is the appropriate quantity to compare to $\omega(t)$. The angular speed of the spheroids is related to the trajectory $\phi_{\text{rot}}(t) = \int_0^t \omega(t')dt'$. The wave speed $\omega_{\text{wave}}(t)$ is related to the trajectory of the wave maximum $\phi_{\text{max}}(t)$, which we obtain from the fit of a sine wave to the kymograph of equatorial velocity fluctuation profiles (section 1.4). Intuitively one might expect that $\omega_{\text{wave}} = \frac{d\phi_{\text{max}}}{dt}$. However, this relation is only true if the instantaneous axis of rotation $\hat{\omega}(t) = \vec{\omega}(t)/|\vec{\omega}(t)|$ is fixed in space: $\hat{\omega}(t) = \hat{\omega}(t+\Delta t)$. The reason for this is that the position of the velocity wave maximum $\phi_{\text{max}}(t)$ is defined with respect to the instantaneous axis of rotation (see section 1.3.3), not with respect to the COM of the spheroids. This means that $\phi_{\text{max}}(t)$ only parameterizes the 3D COM trajectory of the velocity wave (and thus the 3D COM wave speed) if the axis of rotation is fixed in space. To determine the time points where $\hat{\omega}(t) = \hat{\omega}(t+\Delta t)$ is approximately fulfilled, we quantify the movement of the axis of rotation. Specifically, we consider the angle between two subsequent instantaneous axes of rotation $\alpha(t) = \cos^{-1}(\hat{\omega}(t)\cdot\hat{\omega}(t+\Delta t))$. We define rotations as being approximately stable at time t if $\alpha(t) < \alpha_{\text{max}}$ for n subsequent time points where we vary α_{max}. During these time points, we then assume $\hat{\omega}(t) \approx \hat{\omega}(t+\Delta t)$ and thus $\omega_{\text{wave}} \approx \frac{d\phi_{\text{max}}}{dt}$. Furthermore, to consider persistent ordered global rotations, we consider time points t where the rotational order parameter $\Omega(t) > 0.5$. To compare $\omega_{\text{wave}}(t)$ and $\omega(t)$, we compare the trajectories $\phi_{\text{rot}}(t)$ to $\phi_{\text{max}}(t)$ for different values of α_{max} (Fig. S3a). In the limit of small α_{max}, these trajectories reveal that wave propagation follows approximately the same trend as the global rotation. Furthermore, a quantitative comparison between ω and ω_{wave} reveals that for small α_{max}, meaning that the axis of

rotation is relatively fixed, the averages $\langle \omega \rangle$ and $\langle \omega_{\mathrm{wave}} \rangle$ are close (Fig. S3b). However, both quantities exhibit very different standard deviations, which is why we here conclude that on longer time scales, $\langle \omega \rangle \approx \langle \omega_{\mathrm{wave}} \rangle$ for stable rotations.

1.6 Dynamics in the surface layer of spheroids

In the following subsections, we show that (i) most cells reside in the surface layer, which exhibits the largest velocity fluctuations, and (ii) that cell migration is mostly tangential to the spheroid surface in this surface layer. Thus, in the main text we simplify the 3D dynamics of cell spheroids by only considering

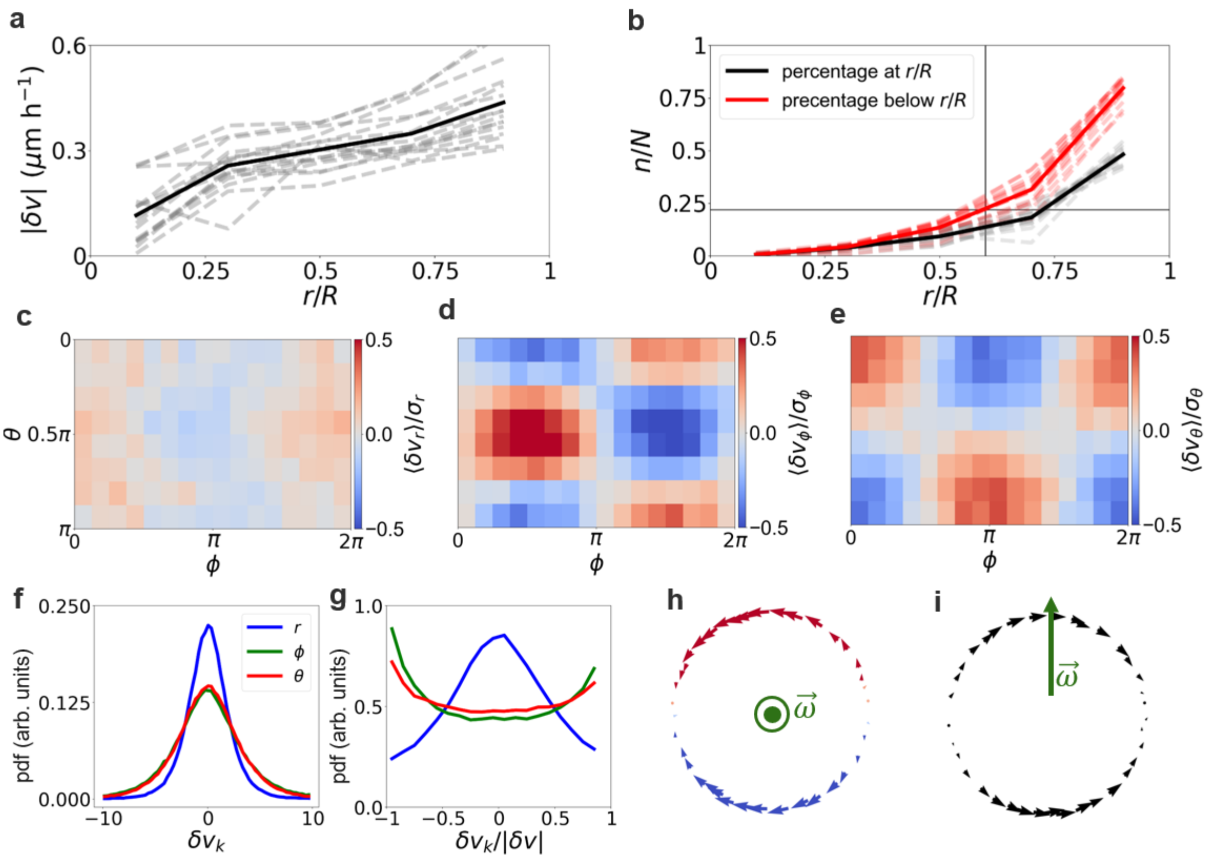

Figure S4: **Tangential surface dynamics. a)** Magnitude of velocity fluctuations as a function of the distance r/R to the COM of the spheroids. Individual gray dashed curves are averages over time of individual spheroids. Black solid line indicates the average over different spheroids. **b)** The ratio of the number of cells within a shell of certain thickness at radius r/R to the total number of cells in the spheroid. Again dashed lines indicate time averages of individual spheroids, while the solid line indicates the average over different spheroids. Red curves show the percentage of cells below a certain distance to the COM of the spheroids. **c)-e)** Signal-to-noise ratio of (c) the radial component δv_r, (d) the azimuthal component δv_ϕ, and (e) the polar component δv_θ in the frame of reference of the propagating velocity wave. **f)** Distributions of δv_ϕ, δv_θ, and δv_r. **g)** Distributions of $\delta v_\phi / |\delta \vec{v}|$, $\delta v_\theta / |\delta \vec{v}|$, and $\delta v_r / |\delta \vec{v}|$. **h)** Planar projection of the average velocity fluctuation field of a slab centered around the equatorial plane. **i)** Planar projection of the average velocity fluctuation field of a slice centered around the y-plane which is perpendicular to the equatorial plane. Green vectors in h and i indicate the axis of rotation of the spheroids.

the tangential velocity fluctuations of the surface layer. This procedure then allows us to visualize the collective dynamics of the cells in the surface layer in spherical coordinates (main text Fig. 2i,k,n, Fig.

3l,m,o). In this subsection we elaborate on this approach.

1.6.1 Importance of the surface layer for the collective dynamics

Regarding point (i), we find that the magnitude of velocity fluctuations increases with the distance to the center of mass of the spheroids (Fig. S4a). Furthermore, the spherical geometry implies that on average, more than 75% of the cells reside beyond of $r_{\text{thresh}} = 0.6R$ (Fig. S4b). Therefore, by focusing on the surface layer, we consider the majority of cells in the spheroids. In addition, most nearest neighbors of cells in the surface layer are also in the surface layers. In other words, cells in the surface layer are expected to interact more often among themselves than with cells in the core. Altogether, by focusing on the surface layer, we capture a majority of cells engaging in the collective dynamics of the spheroids.

1.6.2 Radial components of the dynamics

Regarding point (ii), we find that radial motion in the surface layer into or out of the spheroids is less pronounced than tangential motion in the instantaneous velocity fluctuation field. To show this, we analyze the three components of the velocity fluctuation field $\delta v_\phi = \hat{e}_\phi \delta\vec{v}$, $\delta v_\theta = \hat{e}_\theta \delta\vec{v}$, and $\delta v_r = \hat{e}_r \delta\vec{v}$ in the surface layer of cell spheroids. Not only do the two tangential components exhibit larger values than the radial component (Fig. S4 f), but they also dominate the magnitude velocity fluctuations, which we show by computing the ratio between these components and the total length of the velocity fluctuations $|\delta\vec{v}|$ (Fig. S4 g). Note that if this is the case for the velocity fluctuation field, this is also true for the velocity field, which contains the additional tangential components of the global rotation.

The small radial components in the fluctuation field do not give rise to significant average flows. To show this, we consider δv_ϕ, δv_θ, and δv_r of the surface velocity fluctuation field in the frame of reference co-moving with the velocity wave. By coarse-graining all components through local averaging, we search for collective patterns in the directions of the surface velocity fluctuations (See section 1.7.1 for more details). We consider the signal-to-noise ratio defined by $s/\sigma = |\langle \delta v_k \rangle|/\sigma_k$, where σ_k is the standard deviation of δv_k and k indicates the spatial components in spherical coordinates, i.e. either r, ϕ, or θ. As expected, $\delta v_\phi(\phi, \theta)$ and $\delta v_\theta(\phi, \theta)$ exhibit a significant pattern with four vortices (Fig. S4 d,e). However, δv_r exhibits a signal to noise ratio which is an order of magnitude smaller than the one for the tangential components (Fig. S4c). We thus conclude that the radial component of $\delta\vec{v}$ is not only smaller than the tangential components, but also does not exhibit significant average flows. This is in agreement with the average velocity fluctuation field, which does not show considerable radial motion (Fig. S4h,i).

1.7 Averaging velocity fields in the frame of reference of the velocity wave

To characterize the tangential migratory behavior of cells in the surface layer, we find the average velocity and velocity fluctuation field in a frame of reference that is co-moving with the velocity wave. In the following section we elaborate on this averaging procedure.

1.7.1 General procedure

The average velocity and velocity fluctuation field in the frame of reference of the propagating velocity wave are found by rotating the velocity field $\vec{v}(t)$ and the velocity fluctuation field $\delta\vec{v}(t)$ to align the instantaneous axes of rotation to a new z-axis as described in section 1.3.3. In this frame, we compute

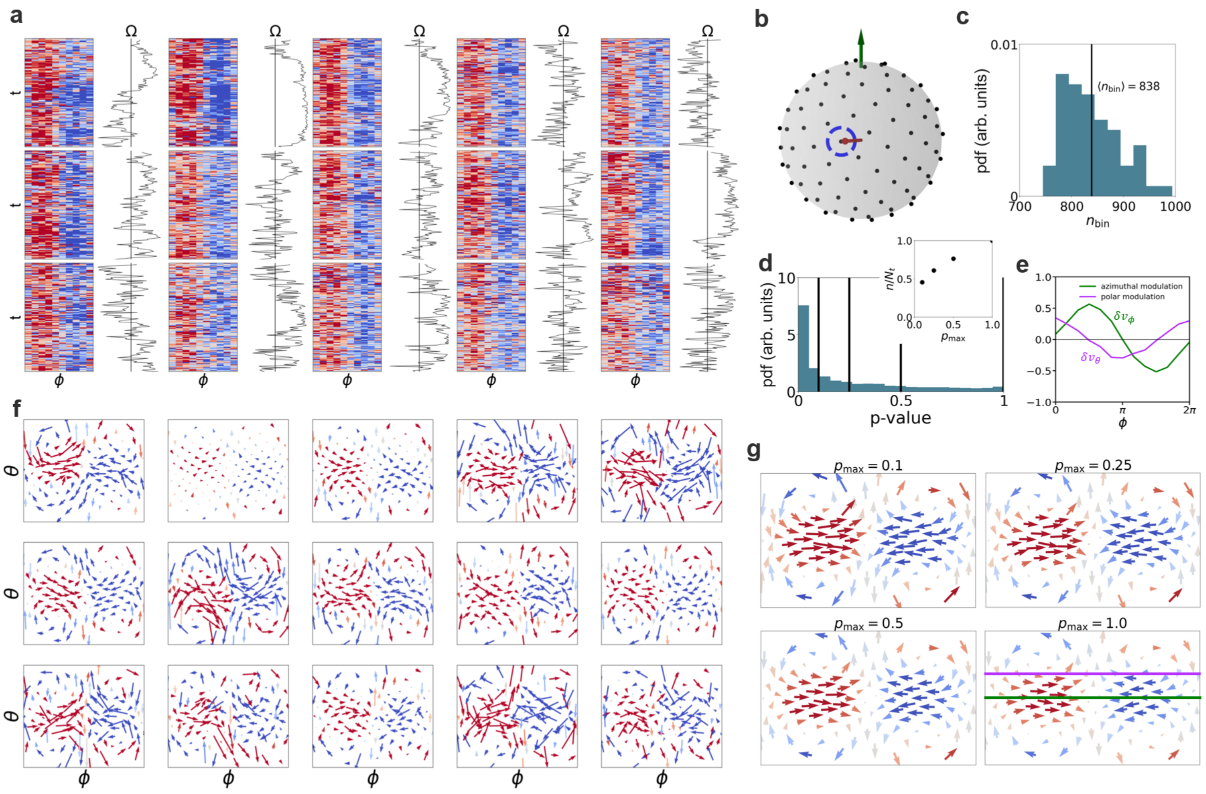

Figure S5: **Averaging procedure. a)** Kymographs of equatorial azimuthal fluctuations for many cell spheroids in the frame of reference, which is co-moving with the equatorial velocity wave. Always to the right we show rotational order in these cancer organoids. The black vertical line indicates a rotational order parameter $\Omega = 0.5$. Right of the black vertical lines indicates $\Omega > 0.5$. The collection of kymographs is ordered according to the radius of the organoids. **b)** Schematic of the binning of our averaging procedure. Green vector indicates the axis of rotation. Red vector indicates the position of a certain bin while the blue circle indicates the margin of this bin. **c)** Distribution of the number of cells n_{bin} that end up in a bin. **d)** Distribution of F-values found from our F-test across all cell spheroids and time points. Vertical lines indicate p_{max}. Inset: Number of time points n where $p < p_{\text{max}}$ divided by the total number of time points N_t in the data. **e)** Profile of the azimuthal component (green) and of the polar component (violet) of the velocity fluctuation field along two distinct latitudes as shown in panel g. **f)** Overview over average tangential velocity fluctuation field averaged only over time points for individual cell spheroids. **g)** Average velocity fluctuation field in the frame of reference co-moving with the velocity wave for four different values for the threshold on the p-value p_{max}.

the kymographs of the equatorial velocity wave. As these kymographs reveal the robust presence of a propagating sinusoidal wave profile, we fit the kymograph $\delta v_\phi(\phi,t)$ by $y_{\text{fit}}(\phi,t) = A(t)\sin(\phi - \phi_0(t))$ which yields the position of the wave maxima as detailed in section 1.4 and 1.5. To transform into the frame co-moving with the wave, we rotate the velocity field and the velocity fluctuation field around the z-axis so that all wave maxima are aligned at $\phi = \frac{\pi}{2}$ (Main text Fig. 2f, Fig. S5a). As a result of this procedure, all instantaneous velocity and velocity fluctuation fields are rotated such that both their axes of rotation and their equatorial wave maxima are aligned.

Then, we non-dimensionalize the velocity and velocity fluctuation fields by scaling down velocities and velocity fluctuations of each cell (indexed by i) by $\omega(t)\, r_i$, where r_i is the distance of the cell to the COM. This yields $\vec{v}_i(t)\,/r_i\omega(t)$ and $\delta\vec{v}_i(t)\,/r_i\omega(t)$. Subsequently, the surface layer of the spheroids is covered in $N_{\text{bins}} = 120$ uniformly distributed circular bins. The size of each bin is defined by an angle $d\Omega = 0.2$ between the position vector of each bin and the position vector of a cell (Fig. S5b). This leads to a coverage of $c = \frac{N_{\text{bins}}\pi d\Omega^2}{4\pi} = 1.2$ of the spheroids by slightly overlapping bins. All rescaled velocities and velocity fluctuations inside one bin are averaged over time and/or different experimental realizations. Averaging over all spheroids results in sufficient statistics (approx. 840 vectors per bin) (Fig. S5c). This procedure yields an average velocity field $\langle\vec{v}/r\omega\rangle_{t,s}$ and velocity fluctuation field $\langle\delta\vec{v}/r\omega\rangle_{t,s}$. We find the tangential components of the result of this averaging procedure and represent them in spherical coordinates as in main text Fig. 2k,n and Fig. 3l,m. We show the tangential component of the average velocity fluctuation field for individual cell spheroids in Fig. S5 f.

This procedure depends on the ability to find and align sinusoidal wave profiles through fitting. During some time points mostly in smaller spheroids, we could not obtain a good fit. To assess if including these time points has an effect on the averages, we make use of the F-test that we outlined in section 1.4, where we find a p-value to assess the statistical significance of the velocity wave profile. To avoid including data points dominated by random fluctuations rather than a statistically significant profile, we exclude data points where the p-value of the fit with a sinusoidal wave profile is large by setting a maximum threshold p_{max} in the range of observed p-values (Fig. S5d). We vary this threshold, which reveals that the large-scale pattern with four vortices in the average velocity fluctuation field is robust to changes in the threshold p_{max} (Fig. S5g). In particular, including only data where the p-value is small (< 0.1), we observe the same characteristics of the velocity and velocity fluctuation fields as for the whole data set. Based on this insight, we thus conclude that our observations are not biased by including data points with large noise. While the large-scale features of the pattern in the velocity fluctuation field do not change with different thresholds, the amount of data which is included is of course affected by the choice of p_{max} (Inset of Fig. S5d). In the main text, we choose $p_{\text{max}} = 0.25$ which includes more than a half of the snapshots of the velocity and velocity fluctuation fields.

1.7.2 Robustness of the average velocity fluctuation field

In this subsection, we demonstrate that the migratory pattern in the average velocity fluctuation showing four vortices is robustly appearing in instantaneous velocity fluctuations throughout the data. To this end, we identify vortices in the tangential velocity fluctuation field $\delta\vec{v}^t$, which we represent in spherical coordinates in main text Fig. 2i for the experimental data.

Approximating the vorticity field

To locate the vortices in the velocity fluctuation field, we compute a proxy for the vorticity field of the tangential velocity fluctuation field $\vec{\nabla}\times\delta\vec{v}^t$ by the use of Stoke's theorem. Specifically, we relate the flow tangential to a circle $\mathscr{C}(\vec{r}, R_{\mathscr{C}})$ centered around a certain position $\vec{r}$ with a radius $R_{\mathscr{C}}$ to the vorticity of the tangential velocity fluctuation field inside the disk $\mathscr{D}(\vec{r}, R_{\mathscr{C}})$ which the circle encloses:

$$\int_{\mathscr{D}(\vec{r},R_{\mathscr{C}})} (\vec{\nabla}\times\delta\vec{v}^t)\,dV = \oint_{\mathscr{C}(\vec{r},R_{\mathscr{C}})} \delta\vec{v}^t\cdot d\hat{l} = \Upsilon(\vec{r}) \tag{S13}$$

Here, the vector $d\hat{l}$ is a unit vector that is tangential to the circle as well as to the spheroid surface. Integrating along the circle yields the line integral of $\delta\vec{v}^t$ along the circle. We aim to find the value of

this integral, which we call $\Upsilon(\vec{r})$. This quantity acts as a proxy to the vorticity and can be interpreted as a local "angular momentum" of the velocity fluctuation field around a certain position $\vec{r}$ parameterized by (ϕ,θ):

$$\vec{r} = R\left(\sin(\theta)\cos(\phi), \sin(\theta)\sin(\phi), \cos(\theta)\right)^T \tag{S14}$$

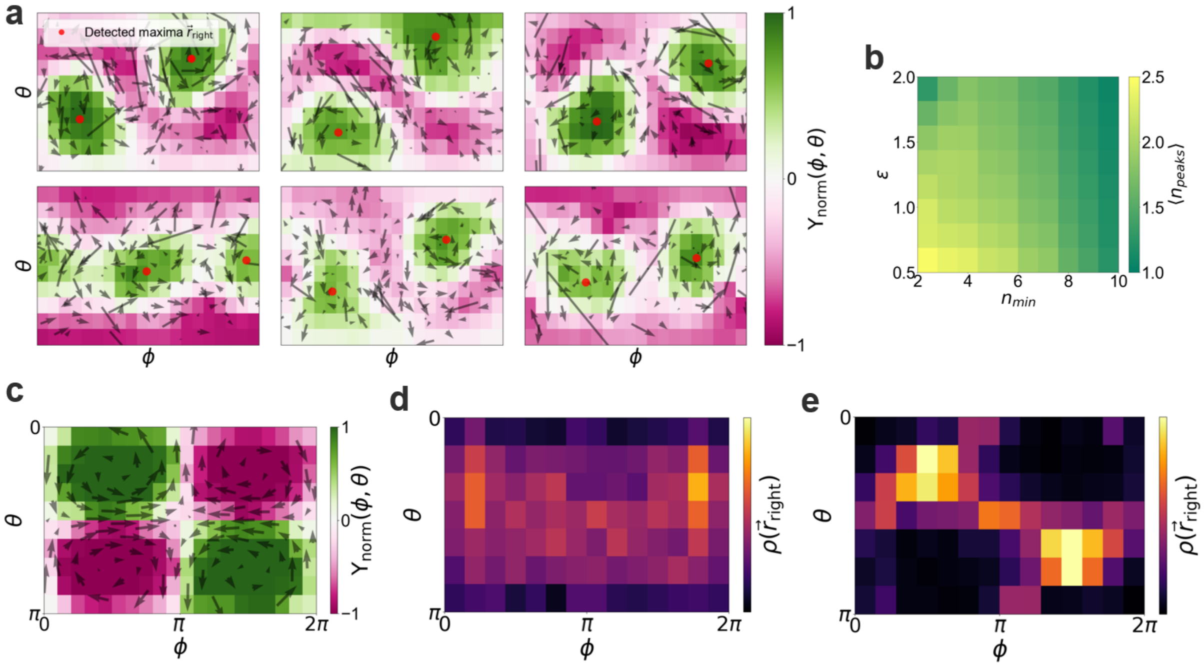

Figure S6: **Robustness of the supracellular pattern.** **a)** Snapshots of the tangential velocity fluctuation field and the normalized vorticity measure $\Upsilon(\phi,\theta)$. **b)** Average number of right handed vortices in the velocity fluctuation field. We vary the two hyper parameters of our clustering algorithm within a reasonable regime dictated by the typical distance of the data points. **c)** $\Upsilon(\phi,\theta)$ of the average velocity fluctuation field. **d),e)** Distribution of the positions of the right-handed swirls $\vec{r}_{\text{right}}$ in the COM frame (d) and in the frame co-moving with the velocity wave (e).

To compute $\Upsilon(\phi,\theta)$ from discrete data, we express the circle radius as $R_{\mathscr{C}} = R\Theta$ where R is the spheroid radius and Θ is an angle between $\vec{r}$ and the positions of the cells $\{\vec{r}_i\}$. We approximate the circle by constraining the angle between $\vec{r}$ and the positions of the cells $\{\vec{r}_i\}$ in the bin $[\Theta - d\Theta, \Theta + d\Theta]$, where we choose $\Theta = 0.8$ and $d\Theta = 0.4$. Furthermore, we focus on the surface layer so we constrain $|\vec{r}_i| > 0.6R$. Then all projections $\delta\vec{v}_i^t \hat{l}_i$ along the circle are summed up to approximate the integral:

$$\Upsilon(\phi,\theta) \approx \sum_{i=1}^{M} \delta\vec{v}_i^t \cdot \hat{l}_i \tag{S15}$$

Here $\hat{l}_i$ is computed at the position $\vec{r}_i$ of the i-th cell in the bin with M cells total being close to the circle. In the following, we consider the 'normalized angular momentum' defined by

$$\Upsilon_{\text{norm}}(\phi,\theta) = \frac{\sum_{i=1}^{M} \delta\vec{v}_i^t \cdot \hat{l}_i}{\sum_{i=1}^{M} |\delta\vec{v}_i^t \cdot \hat{l}_i|} \tag{S16}$$

This normalized quantity takes values of $\Upsilon_{\text{norm}}(\phi,\theta) \in [-1,+1]$, where $+1$ indicates perfect counter clock-wise rotation, and -1 indicates perfect clock-wise rotation as seen from along $\vec{r}$ towards the center of the spheroids.

Patterns in the vorticity field

Snapshots of $\Upsilon_{\mathrm{norm}}(\phi,\theta)$ reveal that the pattern with four vortices found in the average (Fig. S6d) is prominent throughout the data (Fig. S6a). To quantify this robustness, we locate the maxima in $\Upsilon_{\mathrm{norm}}(\phi,\theta)$, which yields the positions of the right handed vortices $\vec{r}_{\mathrm{right}}$. We do so by finding the positions (ϕ,θ) where $\Upsilon_{\mathrm{norm}}(\phi,\theta) > 0.5$. These positions are then clustered by a clustering algorithm called *DBSCAN*. This algorithm identifies clusters dependent on the local maxima in density of data points using two hyper parameters ε and n_{min} [3]. We average the positions within one cluster to find the average position of the local maxima. Within a range of hyper parameters, we find that the velocity fluctuation field exhibits around two right handed vortices (Fig. S6b), which is in agreement with the average velocity fluctuation field (Fig. S6d). In the following, we choose $\varepsilon = 1.0$ and $n_{\mathrm{min}} = 6$ to determine the positions of the vortices. Plotting the positions of these vortices in the COM frame, leads to a flat probability distribution of their positions (Fig. S6d), indicating that vortices propagate. However, the transformation into the frame of reference co-moving with the velocity wave, also aligns the positions of the vortices (Fig. S6e). This shows that transforming into a frame of reference co-moving with the velocity wave can be regarded as equivalent to transforming into a frame of reference co-moving with the four vortices. Thus, the four vortices in the average velocity fluctuation field shown in main text Fig. 3f accurately represent the migratory dynamics at individual time points.

1.8 Analysis of density modulations

In this subsection, we elaborate on how we investigate the cell density along the equator of the cell spheroids in our experiments. To this end, we build a kymograph for the cell density following the procedure outlined for the velocity fluctuation field in section 1.4, but apply it to the cell density: We count the number of cells inside the bins constrained to the equatorial region by $\theta \in [\frac{\pi}{2} - d\theta, \frac{\pi}{2} + d\theta]$, to the surface layer by $r_{\mathrm{thresh}} = 0.6R$, and within the bin of width $d\phi$ in which we found the average azimuthal component of the velocity fluctuation field (Fig. S7a). To approximate the surface density of cells we divide the number of cells in these bins by the surface area of the bins $V = d\phi R^2\left(-\cos(\pi/2 + d\theta) - \cos(\pi/2 - d\theta)\right)$ on the spheroid surface. Complementary to the kymograph describing the velocity wave $\delta v_\phi(\phi,t)$, we thus obtain for all spheroids the kymograph of cell surface density $\rho_{\mathrm{s}}(\phi,t)$. We consider the density fluctuations:

$$\delta\rho_{\mathrm{s}}(\phi,t) = \rho_{\mathrm{s}}(\phi,t) - \langle\rho_{\mathrm{s}}(\phi,t)\rangle_\phi \tag{S17}$$

where we use the average surface density in the equator $\langle\rho_{\mathrm{s}}(\phi,t)\rangle_\phi$. Furthermore, we consider the normalized density fluctuations:

$$\delta\rho_{\mathrm{norm}}(\phi,t) = \frac{\rho_{\mathrm{s}}(\phi,t) - \langle\rho_{\mathrm{s}}(\phi,t)\rangle_\phi}{\langle\rho_{\mathrm{s}}(\phi,t)\rangle_\phi} \tag{S18}$$

1.8.1 Density fluctuations at instantaneous time points

We first investigate possible density fluctuations at instantaneous time points. To this end, we consider the rescaled kymograph of the azimuthal velocity fluctuations $\delta v(\phi,t)/\omega R$ (Fig. S7b) and the kymograph of the normalized density fluctuations $\delta\rho_{\mathrm{norm}}(\phi,t)$ (Fig. S7c) in our model. We quantify the structure of the density fluctuations at instantaneous time points as we did with the velocity wave using the F-test outlined in section 1.4. For the density fluctuations $\delta\rho_{\mathrm{norm}}$, the distribution of the p-values is skewed to large p-values (S7e), indicating that we can't detect a robust sinusoidal wave profile in the density fluctuation

field. This stands in contrast to the rescaled velocity wave $\delta v_\phi(\phi,t)/\omega R$, where the distribution of p-values from our F-test exhibit a peak at small p-values (< 0.1). In conclusion, this shows a qualitative difference between the velocity wave and the density wave. While, we can find a statistically significant sinusoidal profile in the velocity fluctuation field at individual time points, we cannot in the density fluctuation field.

1.8.2 Average density modulations in the co-moving frame

Next, we investigate the average of the velocity wave profile $\langle \delta v_\phi(\phi,t)/\omega R\rangle_{t,s}$ and of the normalized density fluctuation profile $\langle \delta\rho_{\mathrm{norm}}(\phi,t)\rangle_{t,s}$ in the frame of reference of the propagating velocity wave. The average $\langle\rangle_{t,s}$ indicates an average over time and different spheroids. Specifically, to reveal the variance of our average velocity wave and average density fluctuation profile, we perform a bootstrapping analysis. We take the sample size $N = RT$, where R is the number of spheroids and T is the number of recorded time steps. We choose with replacement N randomly chosen samples of the density profile and average them. Some of these bootstrapped averages are shown in Fig. S7f and are analyzed below.

We note that average density fluctuations are small (around 1% of the average density). At the same time, the standard deviation of $\delta\rho_{\mathrm{norm}}(\phi,t)$ across all spheroids, time points, and ϕ-positions is $\sigma_{\mathrm{exp}} = 0.31$ (Fig. S7d). Note that the mean of $\delta\rho_{\mathrm{norm}}(\phi,t)$ across all ϕ-positions is numerically indistinguishable from 0 by definition. Thus, the signal-to-noise ratio for the largest density fluctuations is around $0.01-0.05$. Furthermore, the average density fluctuations are of the order of the error bars (which represent the bootstrapped error of the mean), making it questionable that these average density fluctuations are significant. To assess the statistical significance of the average density fluctuations in an unbiased way, we perform two statistical tests:

First, we determine a detection limit above which the average density fluctuations become statistically significant. To this end, we consider a density fluctuation with an amplitude A, but assume that we can determine A only with standard deviation $\sigma_{\mathrm{exp}} = 0.31$. We define a detection limit for A by performing a two-sample t-test assessing if the means of two normal distributions with the same standard deviation $\sigma_{\mathrm{exp}} = 0.31$, same sample size, but one with a mean of 0 and one with a mean of A are significantly different. We find that A has to be larger than $0.5-0.9\%$ in order to be significant given a confidence interval of 0.95. We give a range, because this threshold depends on the standard deviation σ_{exp}, which in turn depends on the binning parameters that we vary. Average density fluctuations are in general close to this threshold and we do not find a robust profile with statistically significant density fluctuations. We use the threshold of 0.7% as our detection limit for density fluctuations later on in our model.

Secondly, we again perform the F-test outlined above for these bootstrapped averages to find out if these profiles are statistically significant sinusoidal. Again we find a drastic difference between velocity and density: The bootstrapped averages of the density fluctuations exhibit large p-values so they are not significantly better fitted by a sinusoidal wave profile than by a uniform profile. In contrast, the average velocity fluctuation profile exhibits p-values essentially equal to zero, meaning that the average velocity wave profile is significantly sinusoidal. In conclusion, while we cannot strictly rule out finite compressibility in our experimental spheroids, our analysis indicates the absence of significant density fluctuations both in magnitude and in structure.

Finally, we show how the experimental average of the normalized density fluctuations $\langle \delta\rho_{\mathrm{norm}}(\phi,t)\rangle_{t,s}$ and of the average rescaled velocity wave $\langle \delta v_\phi(\phi,t)/\omega R\rangle_{t,s}$ depends on the choice of binning parameters. We vary the threshold $d\theta$ which separates the equatorial region from the polar region as well as

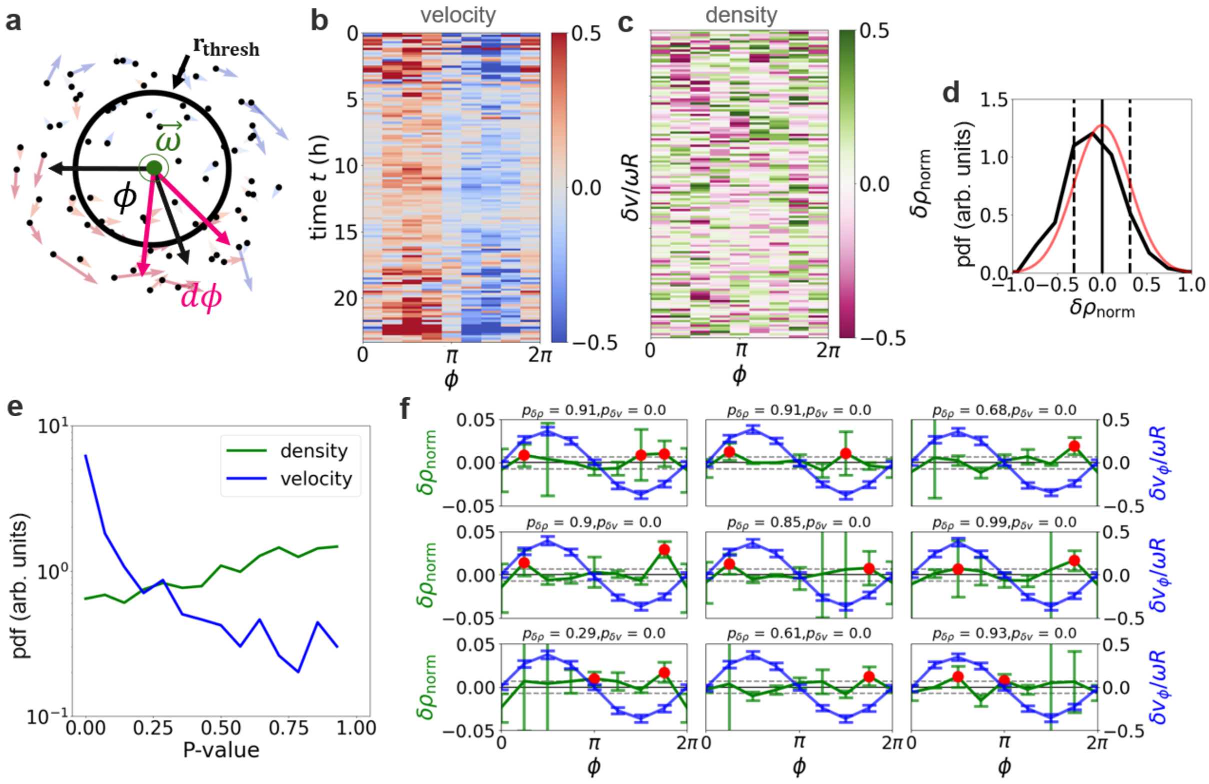

Figure S7: **Quantitative analysis of density modulations in the experiment.** **a)** Schematic of the planar projections of the equatorial velocity fluctuation field. Pink vectors show margins of a bin with width $d\phi$ that we use to bin the position data. **b)** Kymograph of the rescaled velocity wave $\delta v(\phi,t)/\omega R$ along the equator of one spheroid and in the frame co-moving with the wave. **c)** Kymograph of the normalized density fluctuations $\delta\rho_{\text{norm}}(\phi,t)$ along the equator of one spheroid and in the frame co-moving with the wave. **d)** Distribution of normalized density throughout the experimental data (black), which is well approximated by a normal distribution (red). Vertical black dashed lines indicate the standard deviation $\sigma_{\text{exp}} = 0.31$. **e)** Distribution of p-values of the normalized density fluctuations $\delta\rho_{\text{norm}}(\phi,t)$ (green) and for the rescaled velocity wave $\delta v(\phi,t)/\omega R$ (blue). Both distributions show how the p-value is distributed over time and different spheroids. **f)** Nine bootstrapped averages of the experimental velocity wave $\delta v(\phi,t)/\omega R$ (blue) and of the normalized density fluctuations $\delta\rho_{\text{norm}}(\phi,t)$ (green) in the frame co-moving with the velocity wave. Red dots indicate statistically significant density fluctuations above the detection limit of 0.007 (gray dashed horizontal line). The title shows the p-values for the average density and velocity fluctuation profile according to our F-test (1.4).

the threshold r_{thresh} which separates the core of the spheroids from the surface layer. While varying the parameters $d\theta$ and r_{thresh}, we perform the bootstrapping procedure as outlined above, where we choose a subset of the data and average (Fig. S8). Again, we compute p-values quantifying how likely it is that a sinusoidal wave profile provides a better fit to the bootstrapped averages than a uniform profile. We find that the average normalized density fluctuations remains statistically insignificant while varying the binning parameters, which indicates that the choice of binning parameters does not greatly matter.

1.9 Analysis of cell fluxes

We observe that cells at the saddle-point defects behind and ahead of the velocity wave maximum divert towards the poles. To investigate to what degree this motion pattern is compressible, we analyze the cell flux around these defects. In particular, as we show that tangential motion is most dominant in the

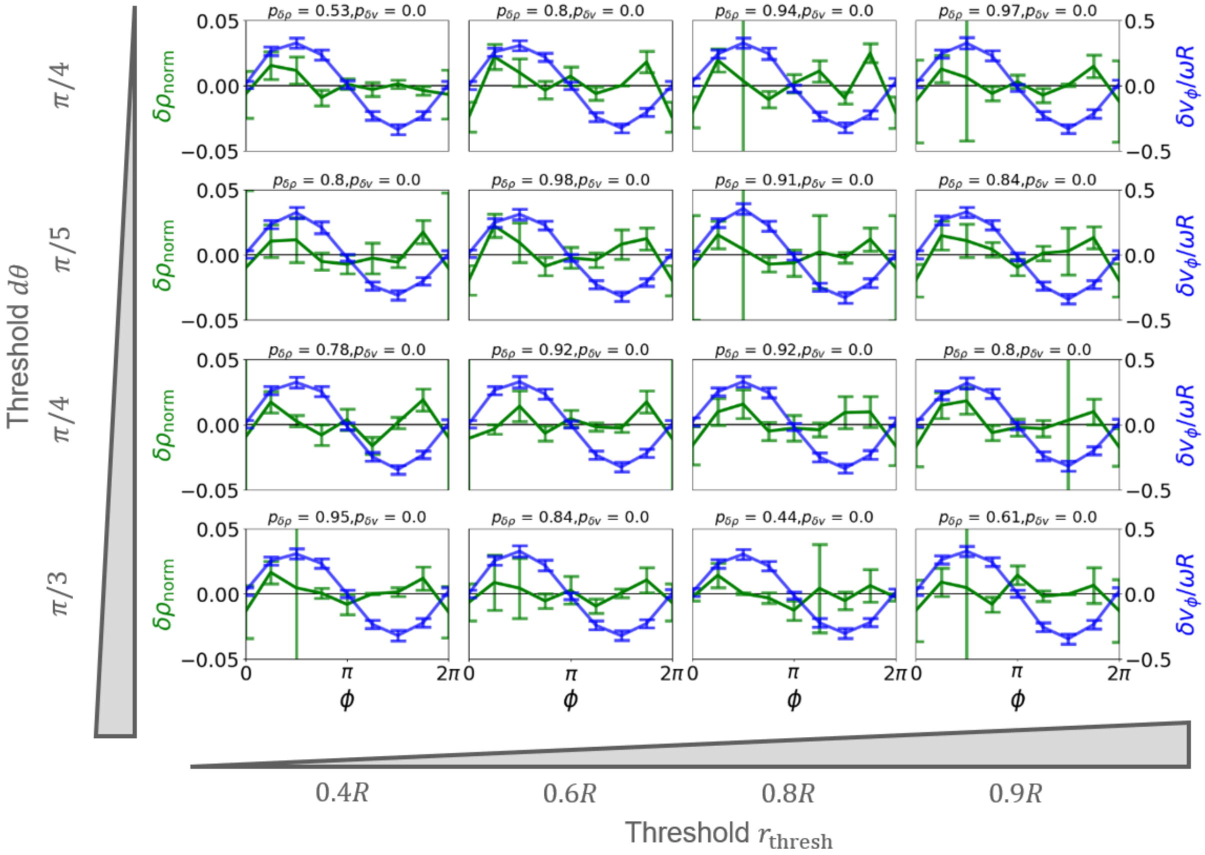

Figure S8: **Binning of density modulations.** Bootstrapped averages of the equatorial azimuthal velocity fluctuations $\delta v(\phi,t)/\omega R$ (blue) and of the density fluctuations $\delta\rho_{\mathrm{norm}}(\phi,t)$ (green) in the frame co-moving with the velocity wave for 16 different parameter combinations of the binning procedure. Horizontally, we vary the threshold between core layers and surface layers. Vertically, we vary the threshold separating the equatorial region from the polar regions.

surface layer of the spheroids (Fig. S4c-i), we consider the instantaneous tangential cell flux along the spheroid surface $\vec{j} = \rho_s \delta\vec{v}^t$, where ρ_s is the surface density of cells in the surface layer of the spheroids and $\delta\vec{v}^t$ is the tangential component of the velocity fluctuation field, which exhibits two saddle-point defects and four vortex defects. Importantly, note that in the following we analyze the fluxes in a frame of reference which is co-moving with the velocity wave and thus also with the vortex defects in the velocity fluctuation field (Fig. S6e). Therefore, the saddle-point defects are on average located at the same positions in this frame. We found no significant density modulation along the equator of the rotating spheroids. Therefore, any net flux through a circle at the surface of the spheroid is related to divergences stemming from cell flux in the radial direction. To assess this possibility, we aim to find the net flux through a circle $\mathscr{C}(\vec{r},R_{\mathscr{C}})$ centered around a certain position $\vec{r}$ with a radius $R_{\mathscr{C}}$:

$$J_{\mathrm{net}}(\vec{r}) = \oint_{\mathscr{C}(\vec{r},R_{\mathscr{C}})} \rho_s \delta\vec{v}^t \cdot d\hat{n} \tag{S19}$$

Here, the vector $d\hat{n}$ is a unit vector that is normal to the circle, pointing away from the center of the circle, and is also tangential to the spheroid surface (Fig. S9a). To compute $J_{\mathrm{net}}(\vec{r})$ from discrete data, we consider a circular cap on the surface of the sphere centered around a certain position $\vec{r}$, which is parameterized by (ϕ,θ):

$$\vec{r} = R\left(\sin(\theta)\cos(\phi), \sin(\theta)\sin(\phi), \cos(\theta)\right)^T \tag{S20}$$

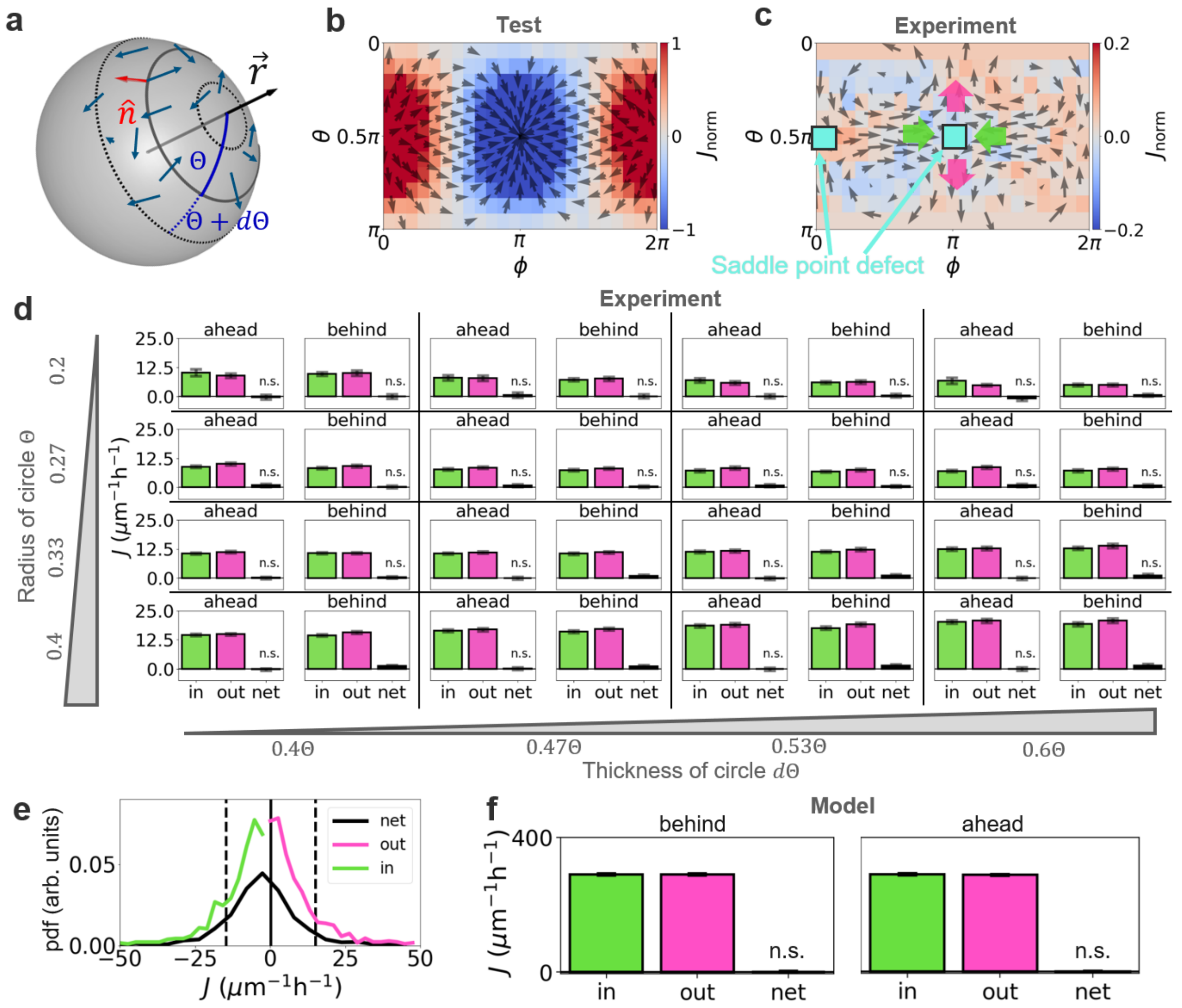

Figure S9: **Analysis of surface cell fluxes.** **a)** Schematic of measuring cell fluxes along the spheroid surface. Blue vectors indicate an example for a tangential vector field. The angle Θ defines the radius of a circle, $d\Theta$ defines the thickness of the circle. **b)** The normalized cell flux $J_{\text{norm}}(\phi,\theta)$ for a test vector field with one source and one sink. **c)** The time and spheroid averaged normalized cell flux $J_{\text{norm}}(\phi,\theta)$ for the experimental data in the frame moving with the velocity wave. **d)** Quantitative analysis of the time- and spheroid averaged net cell flux J_{net} (black) and the time- and spheroid averaged in-(green) and out (pink) flux $J_{\text{in/out}}$ at the positions of the two saddle-point defects ahead and behind the velocity wave (See panel c). Note that for the influx and outflux, we show the absolute value. We vary the parameters Θ and $d\Theta$. Error bars are sometime barely visible but represent the standard error of the mean (s.e.m) computed by bootstrapping. We perform a two-sided t-test to reveal if the average net cell flux is significantly different from 0 (p-values are consistently greater than 0.1). **e)** Distribution of the in- and out flux and of the net flux for $\Theta = 0.33$ and $d\Theta = 0.4\Theta$ as chosen in the main text. Vertical solid line indicates the average net flux and vertical dashed lines indicate the standard deviation of the net flux. **f)** Quantitative analysis of the time- and spheroid averaged net cell flux J_{net} and the time- and spheroid averaged in- and out flux $J_{\text{in/out}}$ in our model in the high repulsion parameter regime ahead and behind the velocity wave maximum.

We express the circle radius as $R_{\mathscr{C}} = R\Theta$ where R is the spheroid radius and Θ is an angle between $\vec{r}$ and the positions of the cells $\{\vec{r}_i\}$ (Fig. S9a). We approximate the circle by constraining the angle between $\vec{r}$ and the positions of the cells $\{\vec{r}_i\}$ in the bin $[\Theta - d\Theta, \Theta + d\Theta]$, where we vary $\Theta \in [0.2, 0.4]$ and $d\Theta \in [0.4\Theta, 0.6\Theta]$. Furthermore, we focus on the surface layer so we constrain $|\vec{r}_i| > 0.6R$. Then all

projections $\rho_s^i \delta\vec{v}_i^t \cdot \hat{n}_i$ inside the circular bin are summed up to approximate the integral:

$$J_{\text{net}}(\phi,\theta) \approx \sum_{i=1}^{M} \rho_s^i \delta\vec{v}_i^t \cdot \hat{n}_i \tag{S21}$$

Here $\hat{n}_i$ is computed at the position $\vec{r}_i$ of the i-th cell in the bin with M cells. Furthermore, ρ_s^i is the local surface density of the spheroid at the position of the i-th cell. We approximate this density by considering the close vicinity of the i-th cell defined by an angle $\Theta_{\rho_s} = 0.8$ between this cell and surrounding cells. We count the number of cells inside this vicinity and divide it by its area, which be approximate as that of a flat disk. This local approximation yields values close to the ones we measure for the global surface density in section 4.3. We then also consider the influx and outflux:

$$J_{\text{in/out}}(\phi,\theta) = \sum_{i=1,\delta\vec{v}_i^t\cdot\hat{n}_i \gtrless 0}^{M} \rho_s^i \delta\vec{v}_i^t \cdot \hat{n}_i \tag{S22}$$

and the normalized net flux through the circle boundary:

$$J_{\text{norm}}(\phi,\theta) = \frac{\sum_{i=1}^{M} \rho_s^i \delta\vec{v}_i^t \cdot \hat{n}_i}{\sum_{i=1}^{M} |\rho_s^i \delta\vec{v}_i^t \cdot \hat{n}_i|} \tag{S23}$$

This normalized quantity takes values of $J_{\text{norm}}(\phi,\theta) \in [-1,+1]$, where $+1$ indicates pure influx while -1 indicates pure outflux. We test our analysis by computing the relative flux for an analytically given tangential vector field that features two fully divergent aster defects (Fig. S9b).

For the experimental data, we find that the average of $J_{\text{norm}}(\phi,\theta)$ over time and different spheroids is in general close to 0 in the frame of reference of the velocity wave. This indicates that on average the majority of cells that tangentially flow through a certain circular region of the surface layer, do this in a way that cell fluxes are apparently balanced (Fig. S9c). We further quantitatively analyze the tangential cell flux around the saddle-point defects ahead and behind the velocity wave. To reveal how sensitive our analysis is, we vary the radius of the circle Θ and thickness of the circle $d\Theta$ used to compute cell fluxes (Fig. S9d). We first determine a detection limit only above which we can measure statistically significant divergences. To this end, we look at the distribution of measured net flux (Fig. S9e). The standard deviation of the net flux is around $18\mu\text{m}^{-1}\ \text{h}^{-1}$. We then ask the following question: Assuming, we found a divergence in the experimental data with an amplitude of D but with a standard deviation as above, how statistically significant are differences between D and 0? This question can be answered by a two-sample t-test assessing if the means of two normal distributions with the same standard deviation $18\mu\text{m}^{-1}\ \text{h}^{-1}$, same sample size, but one with a mean of 0 and one with a mean of D are significantly different. We thus vary D and find a two-sided p-value given a confidence interval of 0.95. We find that D has to be larger than $0.1\mu\text{m}^{-1}\ \text{h}^{-1}$ in order to be significantly different from 0. We find that on average, near the saddle-point regions, the cell influx and the cell out flux are apparently balanced as shown by the average net flux ($0.03\mu\text{m}^{-1}\ \text{h}^{-1}$) being much smaller than the detection limit. Indeed a two-sided t-test to assess if the mean of the net flux is significantly different from 0, reveals that the net flux is statistically insignificant (Fig. S9d). Whether cell fluxes are apparently balanced or not does not sensitively depend on the parameters of our analysis. Finally, we find that also for the model in the high repulsion parameter regime, fluxes ahead and behind of the velocity wave maximum are apparently balanced as again shown by the absence of statistically significant net fluxes (Fig. S9f).

2 Stochastic rigid-body rotation

The correlation function of experimental velocity directions shown in main text Fig. 1c reveals alignment of nearest neighbors and anti-alignment of cells that are on opposite sides of the spheroid. This correlation structure indicates a robust mode of global rotation. Therefore, we consider a stochastic rigid-body rotation as minimal model for the rotational dynamics of the cell spheroids. This model features deterministic rotation with a constant angular velocity everywhere in space and uncorrelated Gaussian white noise. We implement this model via the following Euler-forward scheme:

$$\vec{r}(t+\Delta t) = \mathscr{R}(t)\vec{r}(t) + \sqrt{\Delta t}\vec{\eta}(t) \tag{S24}$$

$$\langle \eta_i(t)\eta_j(t')\rangle = \sigma^2 \delta_{ij}\delta(t-t') \tag{S25}$$

Here, $\mathscr{R}(t)$ is a rotation matrix and $\vec{\eta}(t)$ is uncorrelated Gaussian white noise with amplitude σ. To facilitate the comparison to the experiment, we use the inferred rotation matrix $\mathscr{R}(t)$ from the experimental cell spheroids in our model. Furthermore, assuming that the dynamics beyond the global rotation of the experimental cell spheroids can be described by uncorrelated Gaussian white noise, we determine the noise amplitude at time t from the experimental velocity fluctuations $\delta\vec{v}$ according to

$$3\sigma^2 = \langle \vec{\eta}(t)^2\rangle = \frac{1}{\Delta t}\langle (\vec{r}(t+\Delta t) - \mathscr{R}(t)\vec{r}(t))^2\rangle = \Delta t \langle \delta\vec{v}^2\rangle \tag{S26}$$

Importantly, we observe that $|\delta\vec{v}|$ increases with the distance of the cells to the spheroid's COM (Fig. S4a). To capture this radius dependency in the minimal stochastic rigid-body model, we use a space-

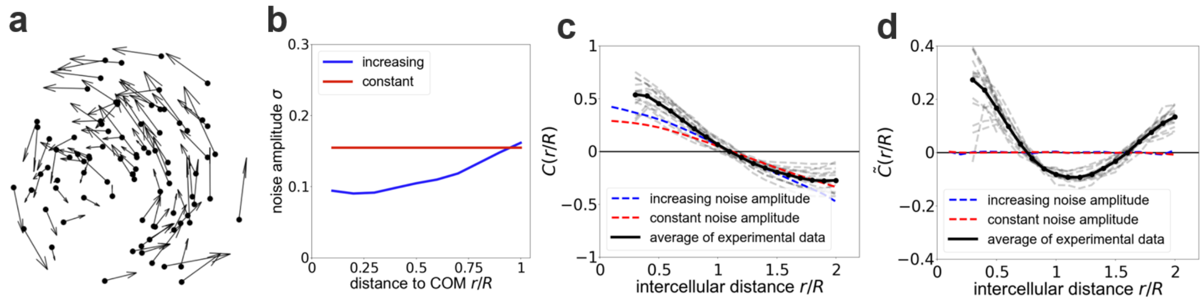

Figure S10: **Stochastic rigid body rotation.** **a)** Snapshot of a velocity field predicted by the rigid body rotation model. **b)** Noise amplitude inferred from the experimental data. **c),d)** Correlation of velocity (c) and velocity fluctuation (d) directions compared between the experiment (black), rigid body rotation with constant noise amplitude (red) and rigid body rotation with increasing noise amplitude (blue).

dependent noise amplitude $\sigma(r)$, where r is the distance of a cell to the COM of the spheroid. We compute this noise amplitude from the experimental data using the following conditional average [4]:

$$\sigma(r) = \sqrt{\frac{\Delta t}{3}\langle \delta\vec{v}^2 || \vec{r}| \approx r\rangle} \tag{S27}$$

We simulate 16 spherical clouds of point particles with the size of the 16 cell spheroids and approximate number of cells in the spheroids (Fig. S10a). We choose the same time scale as in the experiment: $\Delta t = 10$min and 140 frames. From the simulations, we then compute the correlation function of velocity directions and velocity fluctuation directions using equations S9 and S10. The correlation of velocity directions shows alignment at small intercellular distances and anti-alignment at larger intercellular distances for both a constant noise amplitude and a space-dependent noise amplitude. This result shows that

the overall spatial correlations of the velocity field of the global rotational dynamics of cell spheroids can be approximately captured by a stochastic rigid-body rotation. Note that using a constant noise amplitude for the stochastic rigid-body rotation underestimates the correlation at small distances while the space-dependent noise amplitude captures the experimental velocity direction correlation function more quantitatively (Fig. S10b,c).

In contrast, the correlation of velocity fluctuation directions in our stochastic rigid-body model vanishes, which shows no coordination of the velocity fluctuations of this simplified model (Fig. S10d). This is by construction as we impose only uncorrelated noise in addition to the deterministic rotation. This result is in clear disagreement with the experimental data which shows pronounced non-monotonic correlation of velocity fluctuation directions (Main text fig. 2a). This shows that the simplified model of stochastic rigid body rotation is not sufficient to capture the velocity waves that we discover in rotating cell spheroids.

3 Model Implementation

We employ a minimal active particle model commonly used to model collective cell migration [5–10]. Specifically, we model cells as overdamped particles moving on a substrate with effective friction coefficient γ while being self-propelled into the direction of their internal polarization $\hat{p}$ with speed v_0. The polarization itself is subject to rotational noise with amplitude σ and exhibits alignment interactions with strength β. The excluded volume interactions are modeled by the repulsive part of a linear force $\vec{F}^i_{\text{rep}}(r_{ij})$ with amplitude ε parameterizing the stiffness of the soft particles. By this repulsion interaction, the particle has an effective radius λ above which we cut off the attractive force. Following (*34*), we write the equations of motion for these self-propelled particles constrained to a spherical surface as:

$$\vec{v}_i = \mathbf{P}_T\left(v_0\hat{p}_i(t) + \frac{1}{\gamma}\vec{F}^i_{\text{rep}}(r_{ij})\right) \tag{S28}$$

$$\vec{F}^i_{rep}(r_{ij}) = -\varepsilon \sum_{r_{ij}<r_{\text{inter}}} (2\lambda - r_{ij})\hat{r}_{ij} \tag{S29}$$

$$\frac{d\hat{p}_i}{dt} = \dot{\phi}_i(\hat{r}_i(t) \times \hat{p}_i(t)) \tag{S30}$$

$$\dot{\phi}_i = -\beta \sum_{r_{ij}<r_{\text{inter}}} \sin(\phi_i(t) - \phi_j(t)) + \eta_i(t) = -\beta \sum_{r_{ij}<r_{\text{inter}}} (\hat{p}_i(t) \times \hat{p}_j(t)) \cdot \hat{r}_i(t) + \eta_i(t) \tag{S31}$$

$$\langle \eta_i(t)\eta_j(t')\rangle = \sigma^2\delta(t-t')\delta_{ij} \tag{S32}$$

$\mathbf{P}_T$ in equation S28 is a projection operator that projects the forces onto the tangential plane of particle i at position $\vec{r}_i$ [11]. Equation S29 describes the repulsion interaction and contains the inter-particle distance r_{ij} and a vector $\hat{r}_{ij}$ that points from particle i to j. Here, the sum runs over all cells j within a radius of interaction of cell i $r_{ij} < r_{\text{inter}}$. Equation S30 describes the dynamics of a unit vector that can only rotate in the tangential plane around the normal radial vector $\hat{r}_i$ at the position of the i-th particle $\vec{r}_i$. The angle of rotation is determined by equations S31 and S32, which describe alignment and diffusion of the polarity direction.

Free parameters determining the initial conditions of the model are the number of particles N, the radius of the particles λ, the radius of the sphere R, the surface particle density $\rho = N/4\pi R^2$ and the coverage $c = N\pi\lambda^2/4\pi R^2$. We choose $N = 200$, and use the surface density $\rho = 0.01\ \mu\text{m}^{-2}$, which are both close to the experimentally measured surface density. This sets the radius of the sphere $R \approx 40\ \mu$m. Furthermore, we choose $c = 1$ as we do not observe empty regions between cells. This also sets the radius of particles to $\lambda \approx 5.6\ \mu$m. We measure distances in our simulation by $l = 1\ \mu$m. We set the time scale of our simulation to the time scale of self-propulsion $\tau = \frac{l}{v_0}$. Then, to solve equations S28-S32, we employ an Euler-Maruyama scheme in the following algorithm:

1. Initialize the N particles by distributing them equidistantly on the sphere [12].
2. Initialize random polarities and project them onto the surface of the sphere and normalize them afterwards. We find that in the parameter regimes of interest, our results are not sensitive to the initial configuration of the polarities.
3. Propagate the polarities by equation S30 and S31. This equation describes a rotation of the polarities in the tangential plane at the particle positions. As we use finite time steps, this changes the length of the polarities. Therefore, we normalize the polarities after we performed this step.

4. Perform one Euler-step of equation S28 which displaces all particles in a tangential direction which is calculated from the tangential projection of traction force and repulsion interaction. Note that repulsion forces are simply calculated in 3D Cartesian space. Because we use finite time steps, this will result in small particle displacements away from the sphere. We normalize position vectors afterwards and multiply them with the radius of the sphere so that particles stay effectively on the sphere.

5. The displacement of particles also leads to a displacement of polarities. However, a new position defines a new tangential plane which means that old polarities are no longer tangential at the new position. Therefore, we project the polarities again on the sphere and normalize them which allows us to repeat step 3 - step 5 during our simulation.

Selection of parameters, fitting and predicting

Throughout, we use $T = 10^4$ time steps and used a time step $\Delta t = 0.1\tau$. We record the trajectories at a rate of 0.04, yielding 400 time points per trajectory which results in an effective time step in the sampled trajectories of $\Delta t = 2.5\tau$. We do not record any data during the first 100 time steps of the simulation, which ensures that the system has reached steady state. To compare our simulation to the experiment, we rescale time in our simulation by a factor of 0.067. This results in an approximately similar distribution of the average angular velocity, meaning that we match the observation that cell spheroids make between $1-2$ full revolutions within 140 simulation steps as we also observe in the experimental data. We choose the alignment range $r_{\text{inter}} = 2.5\lambda$ to implement nearest neighbor interactions. The choice of this interaction range does not affect the simulations greatly if kept smaller than 3λ. For capturing the experimental data, we first construct the phase diagram shown in main text Fig. 4c and we measure the noise amplitude σ in units of τ^{-1} and vary it logarithmically in $[10^{-3}, 10^{0}]$. We measure the alignment strength β in units of τ^{-1} and vary it logarithmically in $[10^{-2}, 10^{\frac{1}{2}}]$. For the phase diagram shown in main text Fig. 4c, we simulate 30 realizations for each parameter combination. In addition, we vary the strength of the repulsion interaction ε between $\varepsilon = 0.4\ \tau^{-1}$ and $\varepsilon = 3\ \tau^{-1}$. Below $\varepsilon = 0.4\ \tau^{-1}$, we found no sufficient coverage of the sphere. Above $\varepsilon = 3\ \tau^{-1}$ we observed crystallization of particles, which we deemed nonphysical for the cell spheroids that we consider here. To capture the experimental data, we choose a parameter combination to match the distribution of the rotational order parameter to the distribution of the experimental spheroids (main text Fig. 1i). Also, we consider the amplitude of the density wave in the model and choose a repulsion strength such that the amplitude of the density wave falls below our experimental detection limit. This way, we constrain the three key parameters to $\sigma = 0.4\ \tau^{-1}$, $\beta = 0.1\ \tau^{-1}$, and $\varepsilon = 2\ \tau^{-1}$. Note that this parameter combination correctly predicts the relative amplitude of the velocity wave (main text Fig. 4i) as well as the magnitude of the relative velocity fluctuations (main text Fig. 3f-i). Furthermore, we correctly predict the correlation function of the velocity fluctuation directions (main text Fig. 2a). Finally, for a fair comparison of the velocity wave and density fluctuations between experiment and model (Fig. S13i,j), we choose 16 realizations of the simulation, which corresponds to the number of experimental spheroids.

4 Supplementary model results

4.1 Robustness of velocity waves and parameter overview

The velocity wave that we observe in our model is a robust feature in a wide range of parameters within the rotating regime. To show this, we consider the kymograph of the azimuthal equatorial velocity fluc-

tuation profile $\delta v_\phi(\phi,t)$ for each parameter combination. This kymograph reveals a robust sinusoidal wave profile within the collectively rotating regime (Fig. S11a,c). We quantify the robustness of the velocity wave by the F-test as outlined in section 1.4. Specifically, we compute a p-value from fitting the kymographs shown in (Fig. S11a), yielding a phase diagram for p-value of the velocity wave (Fig. S11d). This phase diagram revels the significant presence of the velocity wave within the rotation regime. Within this regime, we compute the average tangential velocity and velocity fluctuation fields following the procedure outlined in section 1.7.1. We show the result of this procedure in Fig. S11b which reveals the robust presence of the pattern with four vortices within the rotating regime. Similar to the experiment, snapshots of the tangential velocity fluctuation field in spherical coordinates reveal that the average velocity fluctuation field are representative for individual time points (Fig. S11e).

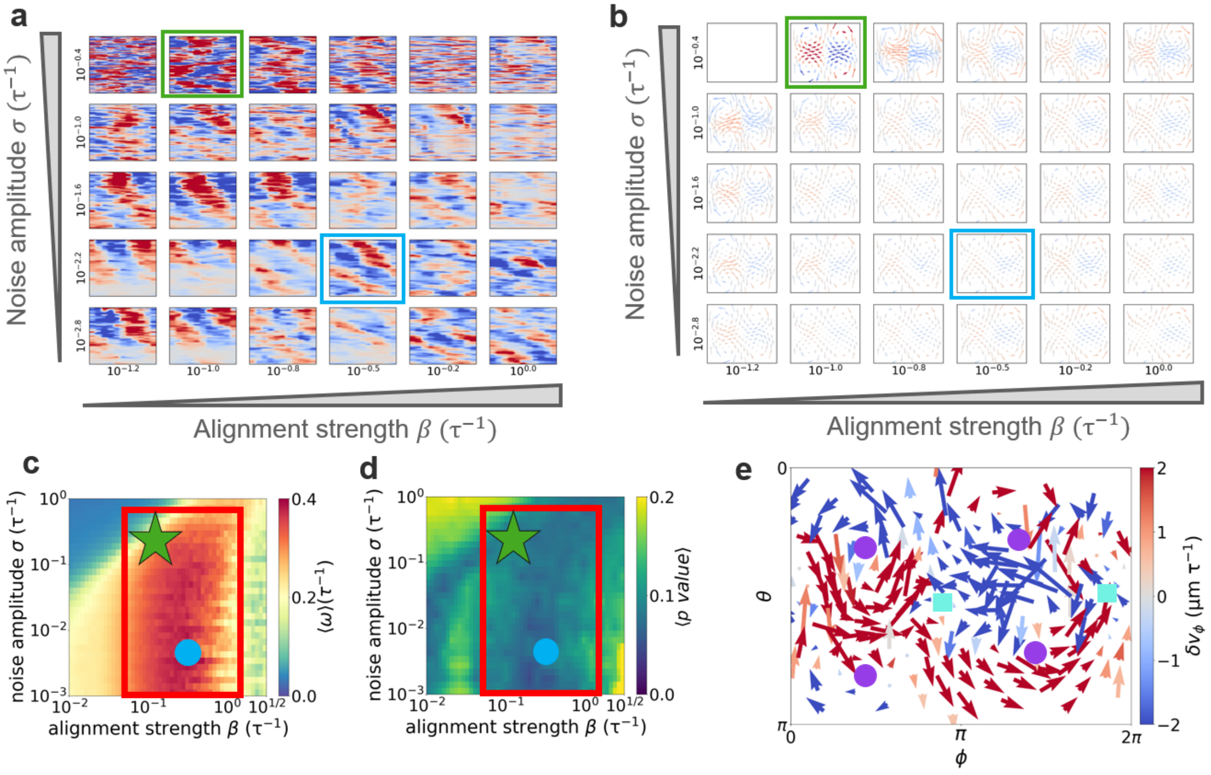

Figure S11: **Robustness of velocity wave.** **a)** Kymographs of the azimuthal equatorial velocity fluctuation profile $\delta v_\phi(\phi,t)$ for the parameter regime where we observe collective rotations in our model. The green box indicates the experimental parameter regime that quantitatively captures the experimental data. The blue rectangle indicates a parameter combination within the low-noise parameter regime of our model. **b)** Tangential components of the average velocity fluctuation field in the frame co-moving with the velocity wave for different parameter combinations. We restrict our averaging of velocity fields to sufficiently large average rotational order parameters: $\langle\Omega\rangle > 0.5$. **c)** Phase diagram constructed from the average angular speed as discussed in the main text. The red rectangle indicates the region for which we show the velocity wave in (a) and the average velocity fluctuation field in (b). The green star indicates the point in the parameter space where the model captures the experimental data. The blue point indicates the low-noise regime of our model. **d)** Average p-value found from the F-test for significant velocity waves. **e)** Snapshot of the tangential velocity fluctuation field represented in spherical coordinates for the experimental parameter regime (green star in (c)). The snapshot reveals the presence of two distinct regions in the equator where one region fluctuates in positive ϕ-direction and the other in negative ϕ-direction with saddle-point defects in between (turquoise squares). Above an below these regions, we observe vortices (violet dots) featuring polar components.

4.2 Size dependence of collective dynamics

In this subsection, we elaborate on how we investigate the effect of the spheroid size on the collective dynamics of the spheroids. Specifically, we investigate if the correlation length of patterns in the velocity fluctuation field scales with the spheroid radius as we hypothesize based on the experimental data. Thus, we consider the experimental parameter regime as we did throughout the paper, but vary the spheroid radius by one order of magnitude between 14μm and 134μm (Fig. S12e). Keeping the particle density constant, this results in varying the number of particles between 25 and 2280. For these different spheroid radii, we compute the correlation function of velocity fluctuation directions $\tilde{C}(r)$ as we do for the experimental data (Fig. S12a,b). If we rescale the intercellular distance by the spheroid radius, we achieve an approximate collapse (Fig. S12c,d). We find the correlation length l_{corr} as the first zero of the correlation function $\tilde{C}(r)$. This correlation length of patterns in the velocity fluctuation field scales with the spheroid radius also for our model (Fig. S12e, Inset of main text fig. 2). Indeed, we find that all considered spheroid sizes in our model exhibit qualitatively the same pattern in the tangential average velocity fluctuation field (Fig. S12f). All together, these results show that correlation structure as well as the velocity wave scale with the system size.

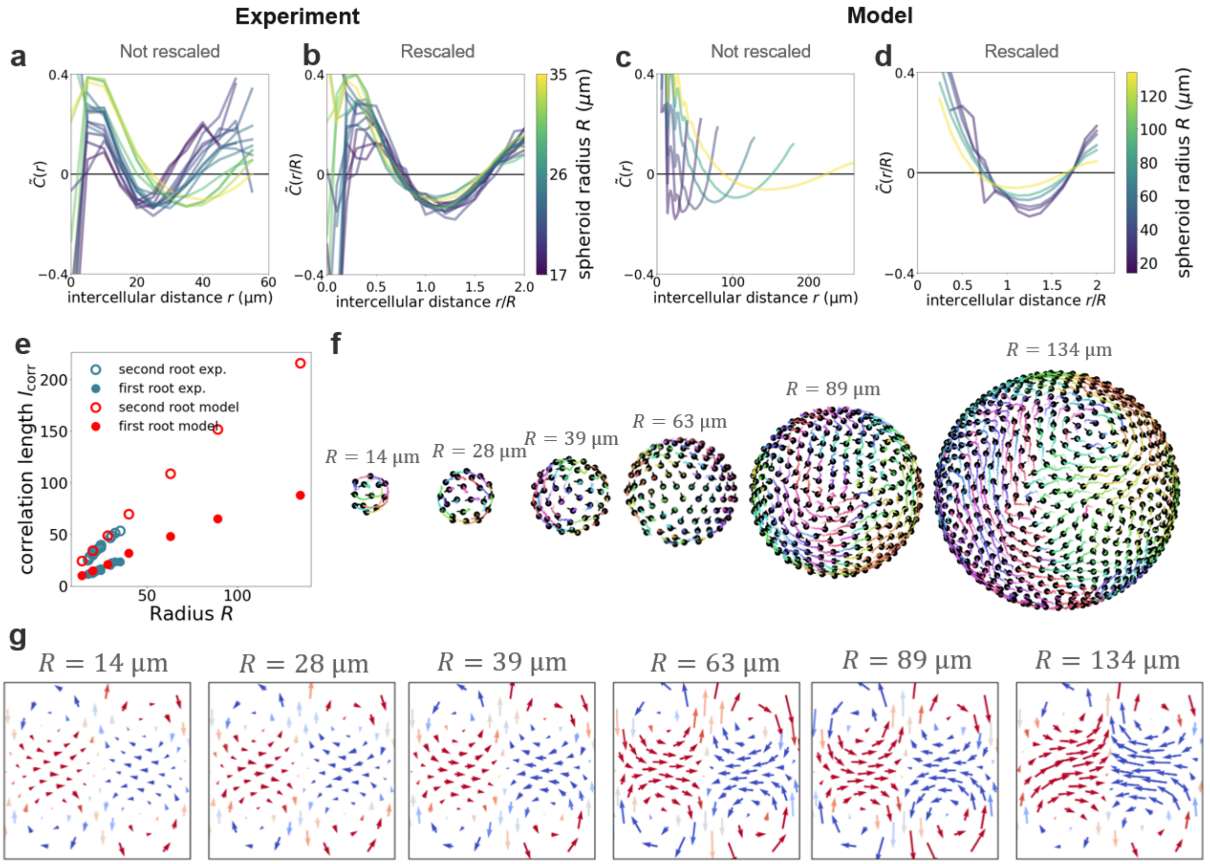

Figure S12: **Size dependence of collective dynamics.** **a),b)** Experimental correlation function of velocity fluctuation directions $\tilde{C}$ as a function of the intercellular distance r (a) and as a function of the rescaled intercellular distance r/R (b). **c),d)** Model correlation function of velocity fluctuation directions $\tilde{C}$ as a function of the intercellular distance r (c) and as a function of the rescaled intercellular distance r/R (d). **e)** Position of the first (solid) and second root (circles) of the u-shaped correlation functions in (a) and (c). Blue dots indicate the experiment, red dots indicate the model. We identify the position of these roots with a correlation length. **f)** Snapshots of the collective dynamics as predicted by our model for six differently sized spheroids. **g)** Tangential components of the average velocity fluctuation field $\langle \delta v(\phi,t)/\omega R \rangle$ in spherical coordinates for the spheroid sizes as shown in (f).

4.3 Analysis of density modulations

In this subsection, we elaborate on our investigation of the cell density along the equator of the cell spheroids in our model. We again consider the rescaled kymograph of the azimuthal velocity fluctuations $\delta v(\phi,t)/\omega R$ (Fig. S13a) and the kymograph of the normalized density fluctuations $\delta\rho_{\text{norm}}(\phi,t)$ (Fig. S13b).

4.3.1 Density fluctuations at instantaneous time points

In a low repulsion parameter regime of our model, the kymograph $\delta\rho_{\text{norm}}(\phi,t)$ shows a density wave that is propagating together with the velocity wave (Fig. S17a,b). We analyze the propagation behavior of this density wave in section 4.5.2. To compare this density wave to our experimental data, we quantify the significance of the density wave at instantaneous time points. For this, we again make use of our F-test outlined in section 1.4. We find that the distribution of the p-values for the density fluctuations is skewed to small p-values at small repulsion strengths ε, but skewed to large p-values for large ε (Fig. S13c). This indicates that while increasing the particle stiffness ε, the amplitude of the sinusoidal density wave profile as observed at low particle stiffness gets not only smaller (main text Fig. 4i,j) with increasing repulsion strength, but also less and less statistically significant. In contrast, the velocity wave remains statistically significant (Fig. S13d) at all values of the repulsion strength. We find that our model is consistent with the experimental data (Fig. S13e,f) in a high-repulsion regime ($\varepsilon = 2\ \tau^{-1}$, $\beta = 0.1\ \tau^{-1}$, $\sigma = 0.4\ \tau^{-1}$).

4.3.2 Average density modulations in the co-moving frame

Next, we investigate the average of the velocity wave profile $\langle \delta v_\phi(\phi,t)/\omega R\rangle_{t,s}$ and of the normalized density fluctuation profile $\langle \delta\rho_{\text{norm}}(\phi,t)\rangle_{t,s}$ in the frame of reference of the propagating velocity wave. The average $\langle\rangle_{t,s}$ indicates an average over time and different spheroids. We find that in the low repulsion regime, our model predicts on average a statistically significant density wave (main text Fig. 4j), consistent with the presence of a statistically significant density wave at individual time points. This density wave is propagating with the same speed of the velocity wave (see Fig. S17a,b,e,f). Thus, it is reasonable to analyze this density wave in the frame of reference of the velocity wave.

While increasing the strength of the repulsion interaction between particles, the amplitude of the density wave decreases, but the density wave keeps accompanying the velocity wave (main text Fig. 4ij, Fig S13g,h). To compare this average density wave to the experimental data, we make use of the detection limit of significant density fluctuations determined in section 1.8. The amplitude of the density wave in our model falls below this detection limit at high repulsion strengths (Fig. S13h), indicating that it is likely that in the experiment, we can't resolve small density amplitudes as predicted by the model in the high repulsion regime. Indeed, we find that if we do a fair comparison between model and experiment by using the same sample size as well as the same number of cells per bin in the model as in the experiment, we find no significant sinusoidal wave profile in our model according to our F-test (Fig. S13 i,j). Altogether, these results suggest that the velocity wave is robust against changes in the compressibility of the spheroids. Furthermore, our results indicate that with the finite amount of experimental data, our model agrees with the experiment in the high repulsion parameter regime.

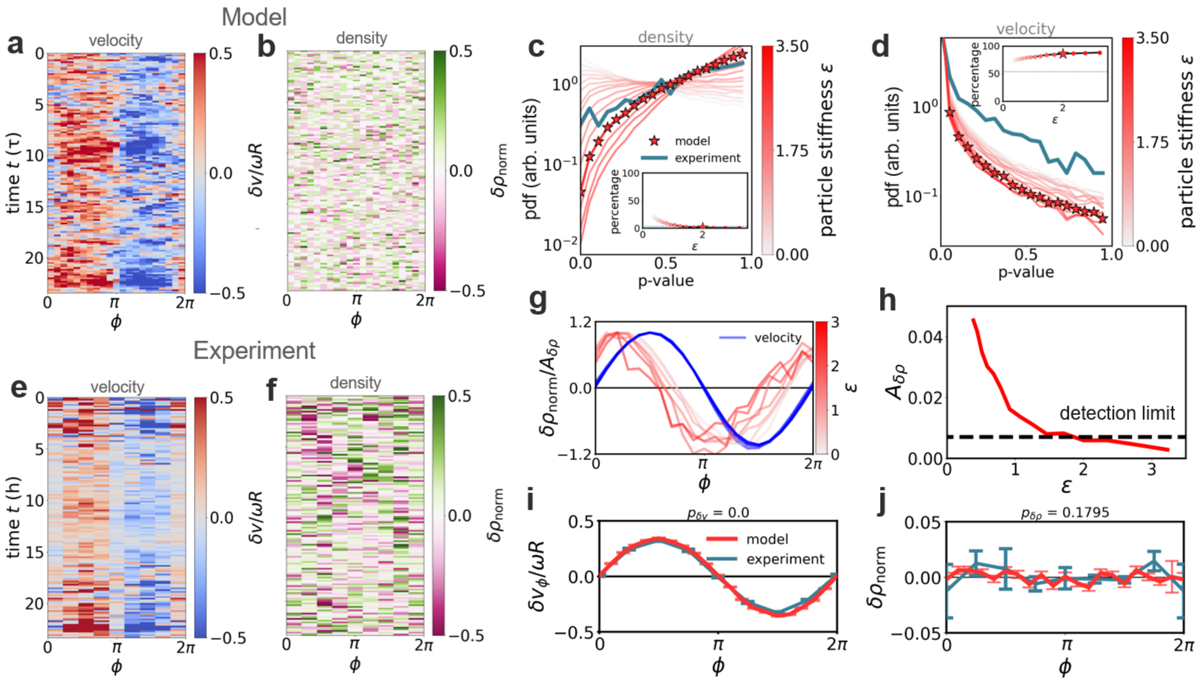

Figure S13: **Quantitative analysis of density modulations in the experiment and model.** **a),e)** Kymograph of the rescaled velocity wave $\delta v(\phi,t)/\omega R$ along the equator of one spheroid and in the frame co-moving with the wave for the model (a) and for experimental data (e). **b),f)** Kymograph of the normalized density fluctuations $\delta\rho_{\text{norm}}(\phi,t)$ along the equator of one spheroid and in the frame co-moving with the velocity wave for the model (b) and for the experimental data (f). **c),d)** Distribution of p-values of the normalized density fluctuations $\delta\rho_{\text{norm}}(\phi,t)$ (c) and for the rescaled velocity wave $\delta v(\phi,t)/\omega R$ (d) for both the experimental data (blue) and for the sweep of different particle stiffness in the model (red). Both distributions show how the p-value is distributed over time and different spheroids. Inset shows the percentage of time points where the p-value is smaller than 0.1. Blue dashed horizontal line in inset shows the experimental percentage. **g)** Density wave from main text Fig. 4j rescaled by its amplitude. Blue curve shows the velocity wave also rescaled by its amplitude. **h)** Amplitude of the density wave in our model for different repulsion strengths ε. Horizontal dashed black line indicates the detection limit (0.7%) of normalized density modulations based on the experimental variability (section 1.8). **i),j)** Rescaled velocity wave $\delta v(\phi,t)/\omega R$ (i) and normalized density fluctuations $\delta\rho_{\text{norm}}(\phi,t)$ (j) averaged over time and different spheroids. Model results are shown in red, experimental results are shown in blue. For the model curve, we averaged the same amount of simulation data as we averaged for the experimental data in (Fig. S7f). The titles shows the p-values for the average velocity fluctuation profile (i) and for the average density fluctuation profile (j) according to our F-test (1.4).

4.4 Testing alternative models

In this subsection, we test two alternative models to reveal how sensitive the emergence of the velocity wave is to (i) the implementation of active particles and (ii) to the dimensionality of the model.

4.4.1 Different alignment interactions

To test if the emergence of velocity waves in our model is specific to the way we implement active aligning motion in our model, we consider a second model for self-propelled particles. The model discussed in the main text and described in section 3 features polarity as an independent degree of freedom next to velocity. To test if the choice of the polarity as an independent degree of freedom is important for

the emergence of velocity waves, we additionally consider a self-propelled particle model where polarity is now enslaved to the velocity as in (*12*). We again model cells as overdamped particles moving on a substrate with effective friction γ. The particles are self-propelled with speed v_0 into the direction of their polarity $\vec{p}_i$, which is now given in terms of the particle velocity. Furthermore, the polarity dynamics include dynamical Gaussian white noise $\vec{\eta}$ with amplitude σ. In this model, the particles interact via repulsion and alignment interactions, which in contrast to the model in the main text, now act on the velocity of the particles. The particles obey the following stochastic equations of motion:

$$\vec{v}_i(t+\Delta t) = \mathbf{P}_T \left(v_0 \frac{\vec{p}_i(t)}{|\vec{p}_i(t)|} + \frac{1}{\gamma} \vec{F}^i_{\text{rep}}(r_{ij}) \right) \tag{S33}$$

$$\vec{F}^i_{rep}(r_{ij}) = -\varepsilon \sum_{r_{ij} < r_{\text{inter}}} (2\lambda - r_{ij}) \hat{r}_{ij} \tag{S34}$$

$$\vec{p}_i(t) = \vec{v}_i(t) + \beta \frac{\vec{V}_i(t)}{|\vec{V}_i(t)|} + \vec{\eta}(t) \tag{S35}$$

$$\langle \eta_i(t) \eta_j(t') \rangle = \sigma^2 \delta(t - t') \delta_{ij} \tag{S36}$$

Again $\mathbf{P}_T$ is an operator that projects vectors onto the tangential plane of the particles. Note that we cut off the repulsion potential beyond λ, which implements pure repulsive interactions.

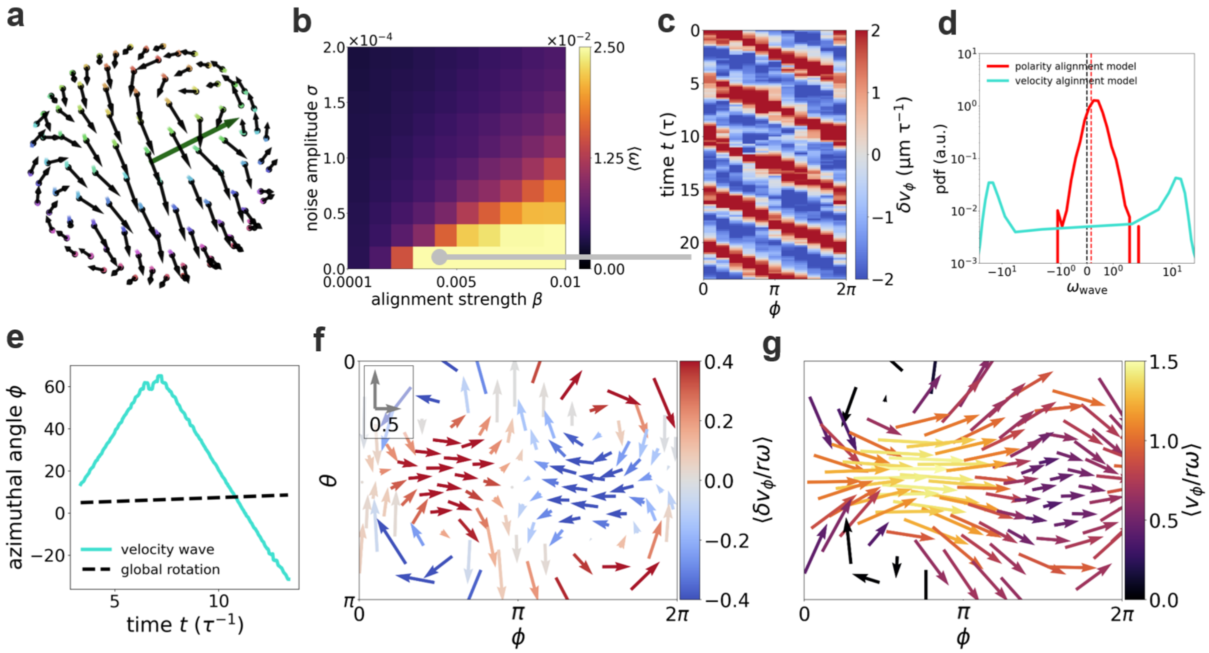

Figure S14: **Velocity waves from velocity alignment.** **a)** Snapshot of the rotational dynamics predicted by a model with alternative alignment interactions. Black vectors indicate the velocity field of the cluster. **b)** Phase diagram of the alternative model constructed from the average angular speed $\langle \omega \rangle$. **c)** Kymograph of the azimuthal component of the velocity fluctuation field in the equator. Grey line shows the parameter combination, that we chose for this kymograph. ($\beta = 0.0045$, $\sigma = 0$) **d)** Distribution of the velocity wave speed in the alternative model (turquoise) and in the model used in the main text (red). **e)** Propagation of the velocity wave in the alternative model (turquoise) compared to the speed of the global rotation (black dashed). **f),g)** Tangential components of the average velocity fluctuation field (d) and the average total velocity field (e) in spherical coordinates as predicted by the model with alternative alignment interactions.

Alignment interactions are implemented by the term $\beta \frac{\vec{V}_i(t)}{|\vec{V}_i(t)|}$ in equation S35, where $\vec{V}_i(t)$ is the average velocity of the M particles in the vicinity of particle i defined by $r_{ij} < r_{\text{inter}}$:

$$\vec{V}_i(t) = \frac{1}{M} \sum_{r_{ij}<r_{\text{inter}}} \vec{v}_j(t) \tag{S37}$$

We initialize the particles and solve their equations of motion using the same scheme as for the main model explained in section 3. We choose $\varepsilon/\gamma = 0.01\ \tau^{-1}$ and again $r_{\text{inter}} = 2.5\lambda$, where λ is again the cut-off of the repulsive force between the particles.

We again reveal a phase diagram for this model by computing the average rotational order and the average angular velocity of the particles (Fig. S14 b). This phase diagram resembles the one we obtain for the model discussed in the main text (Main text Fig. 3c). Importantly, this model also predicts propagating equatorial velocity waves (Fig. S14 c). Unlike in the model in the main text, these waves spontaneously choose between propagating in the direction of the global rotation or against it as shown by the two-peaked distribution of the velocity wave speed (Fig. S14 d). Furthermore, this model predicts significant larger absolute values of the wave speed in relation to the global rotation (Fig. S14 e). However, the alternative model robustly reproduces the global pattern of migration characterized by four vortex defects and two saddle-point defects in the velocity fluctuation field as well as two vortex defects in the total velocity field (Fig. S14 f,g). These results show that the propagation behavior of the velocity wave might depend on model details, but the emergence of velocity wave is not specific to the choice of model.

4.4.2 Varying the dimensionality of our model

In the main text, we describe the observation that most cells reside in the surface layer and that cell migration is predominantly tangential. We therefore simplify the 3D collective dynamics of the rotating spheroids by only considering the outer most cell layers as well as components of the collective dynamics tangential to the spheroid surface. This motivates a 2D model of active particles being constrained to the surface of a sphere. In this subsection, we develop an alternative 3D model, where we do not constrain cells to move on the surface of a sphere, but inside of a spherical confinement. Thus, in this model, we allow for the presence of core cells as well as for radial motion and analyze if these aspects are important for the velocity wave.

We consider a minimal biophysical model of active particles constrained to move within a spherical confinement. This model features the same aspects of collective cell migration as our main 2D model. Specifically, particles self-propel along a polarity vector, which aligns with the polarity vector of neighboring cells with rate β and is subject to dynamical noise with amplitude σ. We implement strong repulsion interactions between particles to reduce overlap and ensure that the particles fill the entire spherical confinement. We use the following equations of motion based on equations (S28-S32):

$$\vec{v}_i = v_0 \hat{p}_i(t) + \frac{1}{\gamma} \vec{F}^i_{\text{rep}}(r_{ij}) + \frac{1}{\gamma} \vec{F}^i_{\text{boundary}}(|\vec{r}_i|) \tag{S38}$$

$$\vec{F}^i_{rep}(r_{ij}) = -\varepsilon \sum_{r_{ij}<r_{\text{inter}}} (2\lambda - r_{ij}) \hat{r}_{ij} \tag{S39}$$

$$\frac{d\hat{p}_i}{dt} = -\sum_{r_{ij}<r_{\text{inter}}} \left[\beta \left(\hat{p}_i(t) \times \hat{p}_j(t)\right) \cdot \hat{n}_{ij}(t) + \eta_i(t)\right] \left(\hat{n}_{ij}(t) \times \hat{p}_i(t)\right) \tag{S40}$$

$$\langle \eta_i(t)\eta_j(t')\rangle = \sigma^2\delta(t-t')\delta_{ij} \tag{S41}$$

The key differences between equations (S38-S41) and equations (S28-S32) are the following: First, we do not project the forces that drive the overdamped motion of particles onto the surface of the sphere (S38), which allows motion in 3D. Secondly, in the 2D version of our model, alignment interactions are implemented by a rotation of the polarity vector in the tangent plane of the particles to minimize angles between the polarities $\hat{p}_i(t)$ and $\hat{p}_j(t)$. In 3D, we minimize the angle between the two polarities, by rotating $\hat{p}_i(t)$ in a plane spanned by the two polarities. We determine this plane by the normal vector

$$\hat{n}_{ij}(t) = \frac{\hat{p}_i(t)\times\hat{p}_j(t)}{|\hat{p}_i(t)\times\hat{p}_j(t)|} \tag{S42}$$

In addition, to constrain motion within a spherical domain, we consider a spherical soft boundary with a size that is comparable to experimental spheroids. This boundary is implemented by the repulsive part of the force defined by

$$\vec{F}^i_{\text{boundary}}(|\vec{r}_i|) = \kappa((R-\lambda)-|\vec{r}_i|)\frac{\vec{r}_i}{|\vec{r}_i|} \tag{S43}$$

where $\vec{r}_i$ is the position of a particle, λ its radius, and R is the spheroid radius. To only consider repulsion between the particle and the boundary, we set $\vec{F}^i_{\text{boundary}}(|\vec{r}_i|) = 0$ if $(R-\lambda) > |\vec{r}_i|$. Furthermore, we chose a sufficiently high boundary stiffness κ to make this boundary fairly stiff and prohibit motion outside of the constraint. We initialize the particles and solve their equations of motion using the same scheme as for the main model explained in section 3. However, in contrast to the 2D model, we initialize our simulation by positioning particles randomly within the spherical confinement and perform a pre-run of the simulation without self-propulsion and alignment interactions to achieve mechanical equilibrium between particles. After the pre-run, particles completely fill the spherical confinement.

When performing numerical simulations with this model, we observed very stable states of non-rotating particles with polarities being completely aligned towards a randomly chosen direction into the confinement (Fig. S15a). In our experiments, cells in the surface layer can form focal adhesions with the spherical basement membrane separating the spheroids from the ECM [13]. Thus, it is likely that cells in the surface layer perform tangential migration on the basement membrane with cell polarization aligned tangentially to this membrane. To test if this aspect leads to global rotation, in our 3D model, we align polarities tangential to the spheroid surface when particles touch the surface. This indeed allows for global rotations (Fig. S15b, Supplementary Movie 7), indicating that tangential polarity is an important aspect of our system. Furthermore, we observed that the repulsion strength ε sets how much the bulk fills the spherical confinement (Fig. S15c). At small ε, only one layer of particles performs global rotations along the confinement. At large ε, we observe particles at the center of the spheroids, as we also observe in our experimental spheroids. Therefore, to have a bulk of cells within our spheroids, we set the repulsion strength to a sufficiently high value. This value ($\varepsilon = 2$) is equal to the repulsion strength in our 2D model, where we capture the absence of significant divergences in the cell flow as well as significant density fluctuations in our experimental spheroids.

With this 3D model we can now assess how sensitive the emergence of the velocity wave is with regards to the presence of a 3D bulk that interacts through alignment and repulsion interactions with the surface layer. First, we vary the alignment strength and the noise amplitude in our model to reveal a

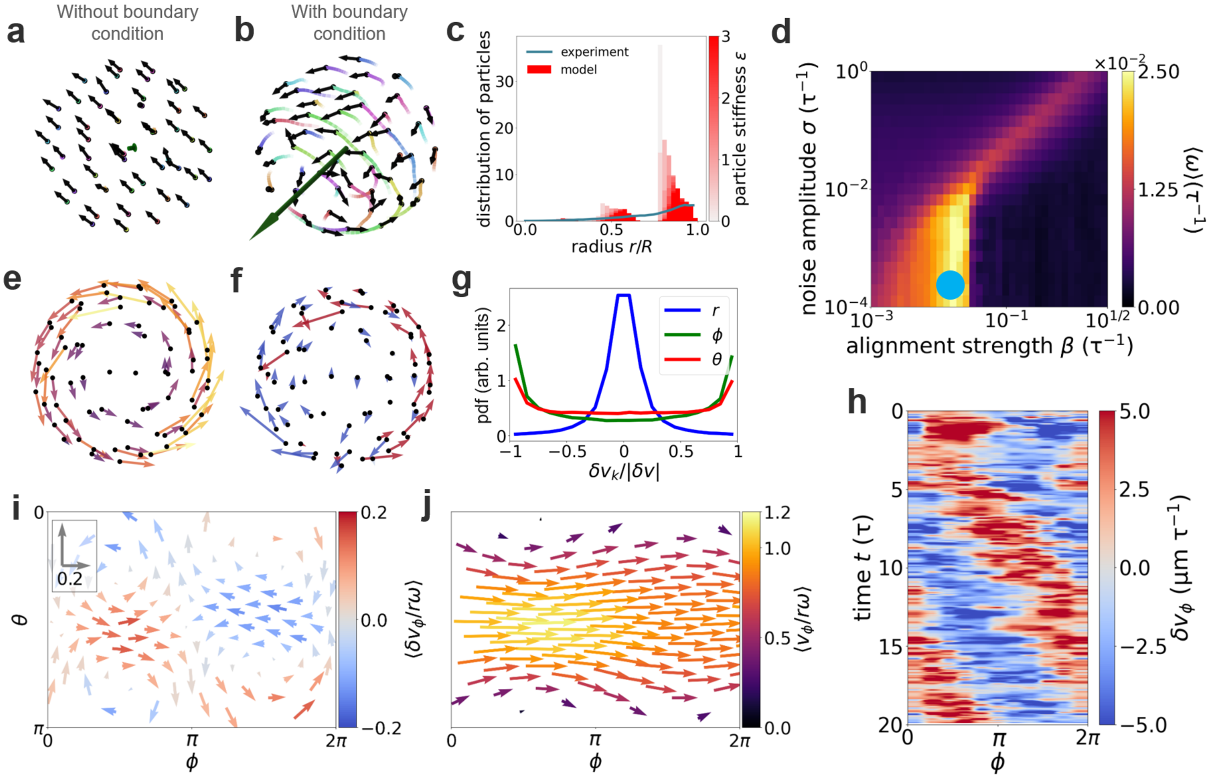

Figure S15: **Velocity waves in a 3D active particle model. a),b)** Snapshot of the rotational dynamics predicted by our 3D model without polarity boundary condition (a) and with polarity boundary condition (b). **c)** Distribution of particle positions as a function of the distance to the COM of the spheroids for varying particle stiffness ε. **d)** Phase diagram of active aligning particles constrained to a sphere constructed from the average angular speed ω. The blue dot indicates the parameter combination, where we investigate the 3D model. **e),f)** Planar projections of the velocity field $\vec{v}$ (e) and of the velocity fluctuation field $\delta\vec{v}$ (f) in a slab centered around the equatorial plane of the spheroid. **g)** Distributions of $\delta v_\phi/|\delta\vec{v}|$, $\delta v_\theta/|\delta\vec{v}|$, and $\delta v_r/|\delta\vec{v}|$ in the 3D model. **h)** Kymograph of equatorial azimuthal velocity fluctuations $\delta v_\phi(\phi,t)$. **i),j)** Tangential components of the average velocity fluctuation field (i) and the average total velocity field (j) in spherical coordinates as predicted by the 3D model in the surface layer of a spheroid.

phase diagram similar to the phase diagram of the 2D model. This phase diagram of 3D active rotations exhibits qualitatively the same features as the 2D diagram (Fig. S15d). Within the parameter regime of low-noise global rotations, we conduct the same analysis of the predicted collective dynamics as we did with our experimental data and for the 2D model. Specifically, we analyze the velocity field (Fig. S15e) and the velocity fluctuation field (Fig. S15f) within the equatorial plane. As in the experimental data, particles move predominantly tangential to the spheroid surface (Fig. S15g) and exhibit little interchange between layers. We again observe a velocity wave that is propagating along the equator of the spheroid (Fig. S15h) with a wavelength equal to the spheroid perimeter. Furthermore, we observe similar supracellular patterns of collective motion on the surface of the spheroids (Fig. S15i,j). Taken together, these results reveal that the emergence of the velocity wave does not sensitively depend on the simplifying assumption that the 3D collective dynamics of the cell spheroids can be approximated by a model describing only active motion on the 2D surface of a sphere.

4.5 Analysis of rotational dynamics of the model and wave propagation

In this subsection, we elaborate on our analysis of the rotational dynamics and on wave propagation in our model.

4.5.1 Wave propagation in the experimental regime

First, we analyze the rotational dynamics of our model in the experimental parameter regime, where our model predicts noisy velocity waves (main text Fig. 4h). In this parameter regime, our model predicts phases of relatively high rotational order and stable axes of rotation sometimes interrupted by reorientations of the axis of rotation (Supplementary Movie 3), as well as break down of rotational order. To investigate the effects of noise on the kymograph of the velocity wave, we compare our kymograph (Fig. S16a) to the rotational order as well as to the dynamics of the axis of rotation, which we quantify by the angle $\alpha(t) = \cos^{-1}(\hat{\omega}(t) \cdot \hat{\omega}(t+\Delta t))$ between the axis of rotation at two consecutive time steps (see Section 1.5). We find that during periods in time, where rotational order is relatively high and reorientations of the axis of rotation are small, the velocity wave shows propagation with a speed approximately equal to the global rotation speed $\omega_{\text{wave}} \approx \omega$ (Fig. S16a-c).

When the axis of rotation undergoes reorientation, results from our analysis of wave propagation in the kymograph have to be interpreted carefully. Specifically, our coordinate transformation, used to align axes of rotation for finding the kymograph (Section 1.3.3), depends on time through the moving axis of rotation. This time dependence can introduce additional dynamics in the kymograph, which can mask actual wave propagation. In addition, when rotational order breaks down, also collective patterns in the velocity fluctuation field commonly break down. As randomly and sometimes backwards moving patterns appear typically during periods where either the axis of rotation undergoes reorientation or rotational order breaks down (Fig. S16a-c), these patterns do not represent a propagating velocity wave. Thus, we ignore these time points when quantifying the propagation of the velocity wave. Note that our model qualitatively captures the exponential decay of the auto correlation function of the axis of rotation (Fig. S16d) and quantitatively the distribution of rotational order (maintext Fig. 1i). Altogether, these results show that even in the presence of high noise, waves robustly appear in our model and propagate with a speed approximately equal to the speed of the global rotation like in the experimental data.

4.5.2 Co-movement of wave, global rotation and polarity defects

Here, we analyze the propagation behavior and the rotational dynamics in our low-noise regime. In order to achieve a better space- and time resolution, we include 1000 particles and a much longer simulation time as we do in the main text. We here consider the low noise regime of our model. First, we observe that the velocity wave in the low repulsion parameter regime of our model and the density wave in our model (Fig. S17a,b) propagate with approximately the same speed (Fig. S17e,f). Furthermore, our analysis reveals long periods in time with only small differences between the speed of the velocity wave, density wave, and global rotation in the low-noise regime of our model, which is consistent with the experimental results (Fig S3). Note that in contrast to the experimental data, in the low-noise regime of our simulation does not show significant fluctuations of the axis of rotation. Therefore, we can assume that the axis of rotation is approximately fixed in space $\hat{\omega}(t) \approx \hat{\omega}(t+\Delta t)$ and we can use the inferred trajectories of the vortices and the velocity wave with respect to the axis of rotation to characterize the 3D COM trajectory of the vortices and velocity wave.

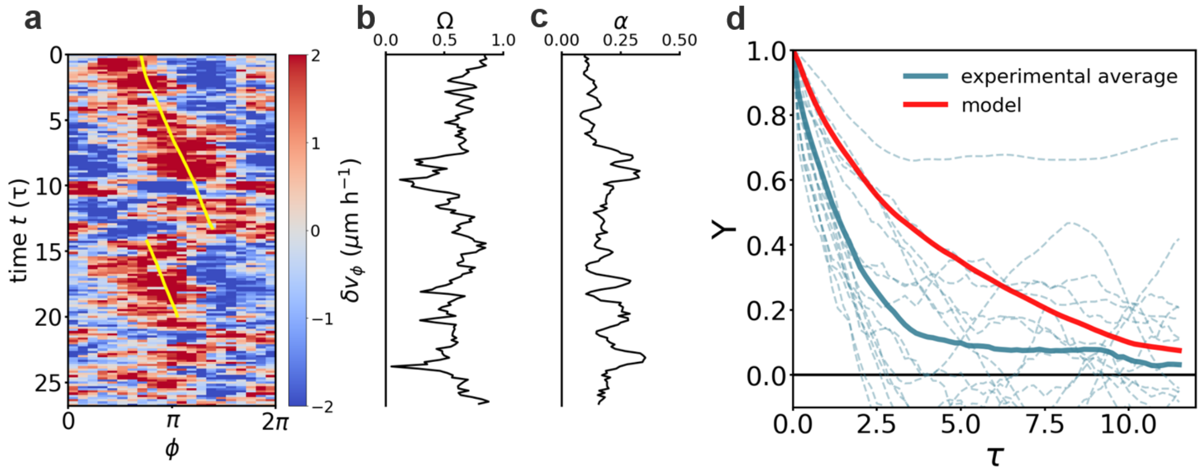

Figure S16: **Wave propagation in the experimental parameter regime.** **a)** Kymograph of the azimuthal component of velocity fluctuations $\delta v(\phi,t)$ along the equator of one spheroid in our model as predicted in the experimental high noise parameter regime. Yellow line shows the trajectory of the global rotation. **b),c)** Rotational order Ω (b) and displacement of the axis of rotation defined as $\alpha(t) = \cos^{-1}(\hat{\omega}(t)\cdot\hat{\omega}(t+\Delta t))$ (c). Both quantities are plotted over time for the same spheroid whose kymograph of azimuthal velocity fluctuations we show in (a). **d)** Auto-correlation function of the axis of rotation $\Upsilon(\tau) = \langle\hat{\omega}(t)\cdot\hat{\omega}(t-\tau)\rangle_t$ for both model (red) and experiment (blue) (section 1.3.2). Blue dashed lines show the experimental auto-correlation for different spheroids.

To further characterize the rotational dynamics of our model, we investigate the vortex defects in the polarity field of our simulation at a low-noise parameter combination of our model. For example, for a rigid-body rotation, we expect the vortex defects to be located exactly at the poles of the global rotation. We find the position of the vortex defects in our model by computing the vorticity measure $\Upsilon(\phi,\theta)$ of the polarity field in the way we defined for the tangential velocity fluctuation field (section 1.7.2). We infer the maxima of $|\Upsilon(\phi,\theta)|$, which correspond to the positions of vortices on the northern and southern hemisphere in spherical coordinates (Fig. S17c). We find that these polarity vortices are displaced away from the poles of the average rotation. The vortex displacements are given in spherical coordinates by

$$\theta_{\text{northern vortex}} \approx 0.15\pi,\ 1-\theta_{\text{southern vortex}} \approx 0.15\pi \qquad \text{(S44)}$$

Here, $\theta_{\text{northern vortex}}$ is the position of the vortex defect on the northern hemisphere in spherical coordinates. Furthermore, we find that these polarity defects propagate around the axis of rotation (Fig. S17e). Interestingly, the azimuthal angle of the displaced vortices coincides with the azimuthal angle of the velocity wave. Furthermore, the speed with which the polarity vortices are propagating is remarkably similar to the propagation speed of the velocity wave that also propagates around the axis of rotation (Fig. S17e,f). These results suggest that the velocity wave including the presence of polar flows in the supracellular pattern of motion is not a random large scale velocity fluctuation in the model, but correlated with active self propulsion towards and over the poles.

4.6 Disentangling the role of topology and curvature

To disentangle the roles of the topology and geometric curvature of spherical surfaces in the emergence of velocity waves, we perturb the spherical geometry of our model.

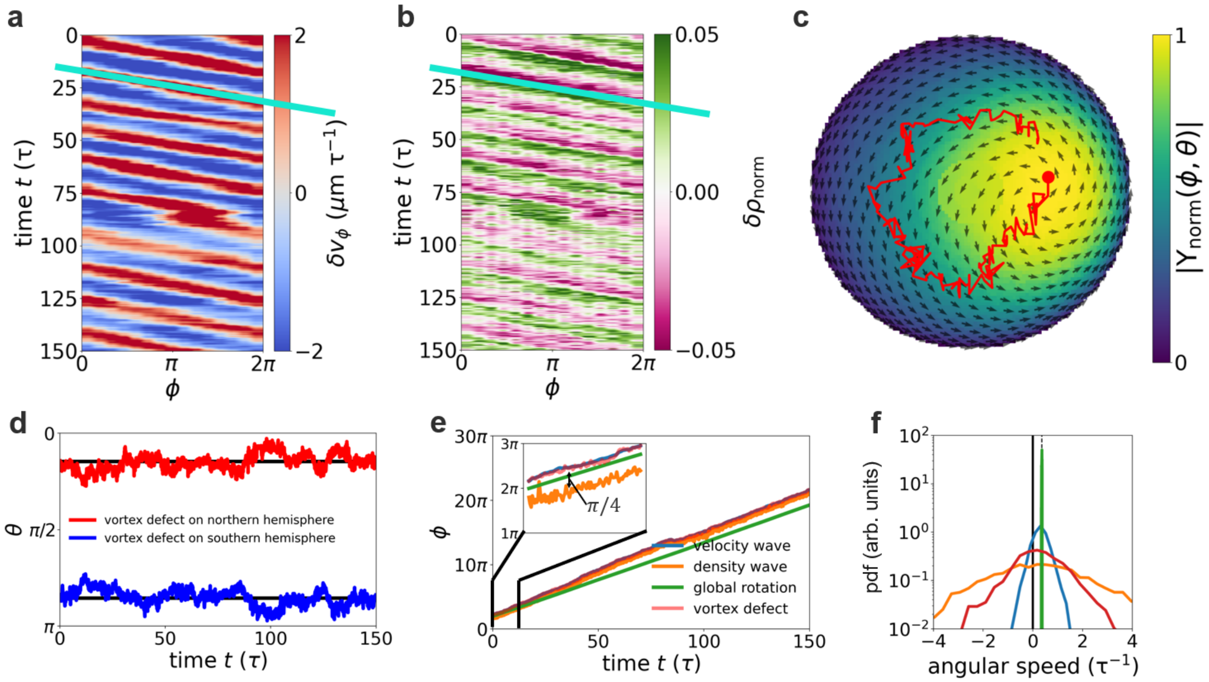

Figure S17: **Rotational dynamics of active particles. a**) Kymograph of the azimuthal component of velocity fluctuations $\delta v(\phi,t)$ along the equator of one spheroid in our model. **b)** Kymograph of the normalized density fluctuations $\delta\rho_{\text{norm}}(\phi,t)$ along the equator of one spheroid in our model. Turquoise line shows the trajectory of velocity wave propagation and has the same slope both in (a) and (b). **c)** Snapshot of a planar projection of the northern hemisphere of the polarity field in our model in front of its vorticity measure $|\mathrm{Y}(\phi,\theta)|$. Red dot indicates the position of the vortex defect and red line shows the trajectory of the vortex defect. **d)** Trajectory of the polar angles $\theta_{\text{northern vortex}}$ and $\theta_{\text{southern vortex}}$ of the two polarity vortex defects. Black vertical lines show the average polar angle of the vortices. **e)** Trajectory of the azimuthal angles of the polarity vortex defect on the northern hemisphere (red), the global rotation (green), the equatorial velocity wave (blue), and the equatorial density wave (orange). The offset of the trajectory of the global rotation is chosen to make comparison easy. Inset shows a shorter time period of the simulation. **f)** Distributions of the speed of the velocity wave (blue), the density wave (orange), the global rotation (green), and the vortex defect (red). Vertical black dashed line indicates the average angular speed of the spheroid.

4.6.1 Active particles on a truncated sphere

First, we consider a truncated sphere: we exclude two diametrically-opposed spherical caps defined by a polar angle of $\Theta = \pi/8$ (Fig. S18b). We implement these regions by defining soft circular boundaries consisting in a linear repulsive force. The two boundaries confine the particles on a ring with positive curvature which is a surface with boundaries and thus topologically distinct from the closed spherical surface. We adjust the number of particles to keep the density, the size of the particles, and the radius of the sphere constant. With a radius of the sphere $R = 40\mu$m, the excluded region defines a circle that spans approximately six times the radius of the particles. Therefore, two particles on two diametrically-opposed points of one excluded region do not interact with each other (radius of interaction $r_{\text{inter}} = 2.5\lambda$).

We consider the parameter combination that predicts low noise rotations and persistently propagating velocity waves on the sphere ($\beta = 0.317\ \tau^{-1}$, $\varepsilon = 2\ \tau^{-1}$, $\sigma = 0.006\ \tau^{-1}$) (Fig. S18a, Supplementary Movie 5). Using this combination on the truncated sphere, we test whether the change of geometry leads to a loss of the velocity wave. Performing numerical simulations reveals that particles perform global rotations along the boundaries around the two excluded regions (Fig. S18b). We find that the velocity

wave still emerges (main text Fig. 5e), together with a global pattern of velocity fluctuations (Fig. S18f). We quantify the robustness of this wave by (i) the F-test used throughout this study to assess if there are significant sinusoidal wave profiles in the kymograph of azimuthal velocity fluctuations $\delta v_\phi(\phi,t)$ and (ii) by looking at the power spectrum of $\delta v_\phi(\phi,t)$. Regarding point (i) we find that $\delta v_\phi(\phi,t)$ on the sphere exhibits in 90% of the time points a p-value of 0.1 or smaller. The truncated sphere exhibits in around 50% of the time points a p-value of 0.1 or smaller. This indicates a less but still robust presence of a sinusoidal wave profile. Regarding point (ii), we consider $\delta v_\phi(\phi,t)$ at time t and do a Fourier transformation in ϕ. Specifically, we find the power spectrum $S(k)$, where k is the wave number of the Fourier modes in ϕ. We normalize this power spectrum by $\sum_{k_{\min}}^{k_{\max}} S(k)$. Finally, we average the normalized power spectrum over time and over different spheroid realizations. The result of this procedure is shown in Fig. S18d. As for the closed sphere, we find that the average normalized power spectrum of the velocity wave on the truncated sphere exhibits a sharp peak at $k = 1$ (Fig. S18d), which represents is the velocity wave with its wave length equal to the spheroid perimeter. Together, these results suggest a robust presence of a sinusoidal velocity wave on the truncated sphere that has a wave length equal to the perimeter of the sphere.

Finally, we consider a parameter combination ($\beta = 0.317\ \tau^{-1}$, $\varepsilon = 2.0\ \tau^{-1}$, $\sigma = 0.25\ \tau^{-1}$) that is close to the experimental parameter regime of the sphere, which we used to reproduce the experimental data (Section 3). Here, we also observe a velocity wave (Fig. S18h) accompanied by a supracellular flow pattern with topological defects (main text Fig.5h). In conclusion, our results suggest that the velocity wave still emerges on truncated spheres that are topologically distinct from spheres.

4.6.2 Active particles on cylinders

To further disentangle the role of topology and curvature, we consider a cylinder, i.e. a geometry with the same topology as the truncated sphere but with zero Gaussian curvature. We implement this model by constraining the dynamics of particles on the surface of a cylinder. Specifically, we use equations S28 - S32, but implement the cylindrical geometry by using a different normal vector $\hat{n}$ of the underlying surface. For the sphere, we used the normalized position vector of a cell $\hat{r}_i$ as normal vector. Projection of the forces on the tangential plane of a particle was done using this vector (equation S28). Also rotation in the tangential plane of polarity vectors due to noise and alignment interactions was done using this vector (equation S30 and S31). For the cylinder, we replace this vector by the normal vector of the cylindrical surface $\hat{n}_i = (\cos(\phi_i), \sin(\phi_i), 0)$ at the position of the i-th cell. Finally, we introduce two soft boundaries perpendicular to the z-axis of the cylinder and separated by a height h. Here, we choose the softness of these boundaries to be very low. Furthermore, we choose $N = 250$ particles, and use the same experimental surface density of particles as we did with the sphere. This means that the radius of particles is set to 5.6 μm at a coverage $c \approx 1$. We furthermore set the radius of the cylinder to $R \approx 40\ \mu$m, which is the same size of the spheres that we studied before. Together with the number of particles and the coverage this yields a cylinder height of $h \approx 56\ \mu$m (Fig. S18c).

Changing the geometry from a sphere to a cylinder requires an additional adjustment of the alignment interactions of the model. Note that we implement alignment interactions on the sphere by finding the angle between two polarity vectors and then rotating the polarity of a cell in its tangential plane to decrease this angle. Importantly, the angle is computed as the projection of the cross product of two 3D polarity vectors on the surface normal (equation S30 and S31). Applying the same alignment interaction on the cylinder leads to very stable states where all polarities are aligned along the z-axis of the cylinder, which prohibits collective rotations. For a sphere, such a special state where all 3D polarities can be completely aligned while being tangential does not exist. Therefore, we adjust the alignment interaction

for the cylinder: instead of finding the angle between the 3D polarity vectors, we parallel-transport the polarity vectors onto each other to find an angle between them in one local tangent plane of the specific particle whose polarity dynamics, we compute. This implementation thus considers vectors to be parallel when they have the same components in cylindrical coordinates, which no longer leads to a favored direction, and thus allows persistent rotations around the z-axis. We also performed tests to show that this alternative alignment interaction does not affect the collective dynamics of the particles on a sphere. In these tests, we found no differences in the rotational behavior as well as in the properties of the velocity wave.

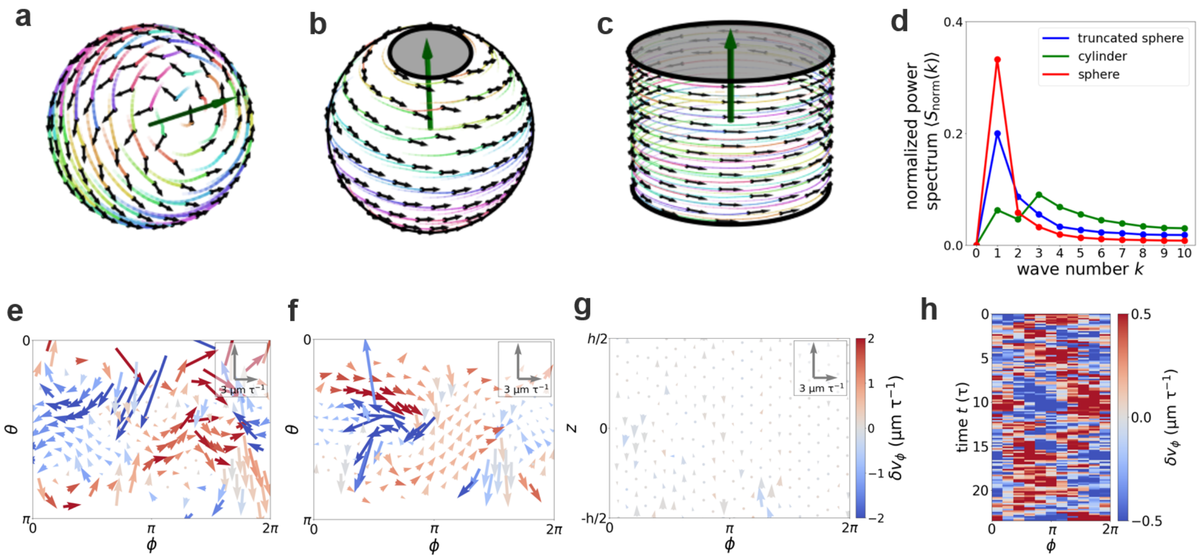

Figure S18: **Active particles on varying geometries.** **a)-c)** Snapshots of the dynamics of active particles on a sphere (a), on a truncated sphere with deterministic rotations (b), and on a cylinder (c). Throughout, we use the same parameter combination ($\beta = 0.317$, $\varepsilon = 2.0$, $\sigma = 0.006$). **d)** Normalized power spectrum of the kymographs of the three different considered geometries shown in main text Fig. 5d-f. **e)-g)** Snapshot of the tangential velocity fluctuation field in spherical/cylindrical coordinates for the sphere (e), for the truncated sphere (f), and for the cylinder (g). **h)** Kymograph of the azimuthal velocity fluctuations $\delta v_\phi(\phi,t)$ of the truncated sphere in an experimentally relevant parameter regime.

We initialize the particles on the cylinder with purely azimuthal polarities and solve the model numerically with $T = 10^4$ and a time step $\Delta t = 0.1\tau$. We record the trajectories at a rate of 0.04, yielding 400 time points per trajectory which results in an effective time step in the sampled trajectories of $\Delta t = 2.5\tau$. We do not record any data during the first 100 time steps of the simulation, which ensures that the system has reached steady state. To compare our simulation to the experiment, we rescale time in our simulation by a factor of 0.067 as in our simulation of the sphere and truncated sphere. We choose $\sigma = 0.006\ \tau^{-1}$, $\varepsilon = 2\ \tau^{-1}$ and $\beta = 0.317\ \tau^{-1}$ and repeat the simulation 10 times. Importantly, this is the same parameter combination that we used for both the sphere (Fig. S18a) and the truncated sphere (Fig. S18b). We analyze the emerging global rotations on the cylinder (Fig. S18c, Supplementary Movie 6) and look for velocity waves in the azimuthal component of the velocity fluctuation fields $\delta v_\phi(\phi,t)$ as we did for the sphere. First, azimuthal velocity fluctuations are an order of magnitude smaller compared to the complete and the truncated spheres (main text Fig. 5d-f, Fig. S18e,f,g), indicating that the global rotations are barely modulated on a cylinder. We quantify $\delta v_\phi(\phi,t)$ on the cylinder in the same way as we did on the sphere and on the truncated sphere. Specifically, (i) we again consider the F-test used throughout this study to assess if there are significant sinusoidal wave profiles in $\delta v_\phi(\phi,t)$, (ii) and analyze the power spectrum of $\delta v_\phi(\phi,t)$. Regarding point (i), we are not able to detect a robust statistically significant large-scale velocity wave profile modulating the global rotation while persistently propagating in the az-

imuthal direction. Specifically, we find on the cylinder that $\delta v_\phi(\phi, t)$ in only a fraction of 18% of time points exhibits a p-value of 0.1 or smaller. In contrast, $\delta v_\phi(\phi, t)$ on the sphere exhibits in 90% of the time points a p-value of 0.1 or smaller. Regarding point (ii), we do not find a sharp peak at $k = 1$ in the normalized power spectrum of $\delta v_\phi(\phi, t)$, but instead observe a broad and flat peak at higher wave numbers (Fig. S18d). This suggests that $\delta v_\phi(\phi, t)$ on the cylinder does not exhibit a clear large-scale pattern reminiscent of the velocity wave appearing on spheres and truncated spheres.

Supplementary References

[1] Attanasi A, Cavagna A, Del Castello L, Giardina I, Melillo S, Parisi L, Pohl O, Rossaro B, Shen E, Silvestri E, *et al.* 2014 Collective Behaviour without Collective Order in Wild Swarms of Midges. *PLoS Computational Biology* **10**, 7, 1–10. (doi:10.1371/journal.pcbi.1003697).

[2] Bardsley WG, McGinlay PB, Wright AJ. 1986 The F Test for Model Discrimination with Exponential Functions. *Oxford University Press* **73**, 2, 501–508.

[3] Ester M, Kriegel HP, Sander J, Xiaowei X A density-based algorithm for discovering clusters in large spatial databases with noise. *Proceedings of the Second International Conference on Knowledge Discovery and Data Mining (KDD-96)* pp. 226–231.

[4] Brückner DB, Fink A, Schreiber C, Röttgermann PJ, Rädler JO, Broedersz CP. 2019 Stochastic nonlinear dynamics of confined cell migration in two-state systems. *Nature Physics* **15**, 6, 595–601. (doi:10.1038/s41567-019-0445-4).

[5] Smeets B, Alert R, Pešek J, Pagonabarraga I, Ramon H, Vincent R. 2016 Emergent structures and dynamics of cell colonies by contact inhibition of locomotion. *Proceedings of the National Academy of Sciences of the United States of America* **113**, 51, 14621–14626. (doi:10.1073/pnas.1521151113).

[6] Szabó A, Mayor R. 2016 Modelling collective cell migration of neural crest. *Current Opinion in Cell Biology* **42**, 22–28. (doi:10.1016/j.ceb.2016.03.023).

[7] Copenhagen K, Malet-Engra G, Yu W, Scita G, Gov N, Gopinathan A. 2017 Frustration induced phases in migrating cell clusters. *arXiv* , September, 1–10.

[8] Sepúlveda N, Petitjean L, Cochet O, Grasland-Mongrain E, Silberzan P, Hakim V. 2013 Collective Cell Motion in an Epithelial Sheet Can Be Quantitatively Described by a Stochastic Interacting Particle Model. *PLoS Computational Biology* **9**, 3. (doi:10.1371/journal.pcbi.1002944).

[9] D'alessandro J, Solon AP, Hayakawa Y, Anjard C, Detcheverry F, Rieu JP, Rivière C. 2017 Contact enhancement of locomotion in spreading cell colonies. *Nature Physics* **13**, 10, 999–1005. (doi:10.1038/nphys4180).

[10] Alert R, Trepat X. 2020 Physical Models of Collective Cell Migration. *Annual Review of Condensed Matter Physics* **11**, 77–101. (doi:10.1146/annurev-conmatphys-031218-013516).

[11] Sknepnek R, Henkes S. 2015 Active swarms on a sphere. *Physical Review E - Statistical, Nonlinear, and Soft Matter Physics* **91**, 2, 1–13. (doi:10.1103/PhysRevE.91.022306).

[12] Evenly distributing n points on a sphere. https://stackoverflow.com/questions/9600801/evenly-distributing-n-points-on-a-sphere. Last accessed: 2023-02-06.

[13] Han YL, Pegoraro AF, Li H, Li K, Yuan Y, Xu G, Gu Z, Sun J, Hao Y, Gupta SK, *et al.* 2020 Cell swelling, softening and invasion in a three-dimensional breast cancer model. *Nature Physics* **16**, 1, 101–108. (doi:10.1038/s41567-019-0680-8).
