# Supplementary material for: Curvature induces active velocity waves in rotating spherical tissues
Source: Nat Commun. 2023 Mar 24;14:1643. doi: 10.1038/s41467-023-37054-2 (PMC10039078; doi:10.1038/s41467-023-37054-2)
Supplement: Supplementary file 2 — Description of Additional Supplementary Files [file 41467_2023_37054_MOESM2_ESM.pdf]

Description of Additional Supplementary Files

File Name: Supplementary Movie 1
Description: Time lapse of fluorescence microscopy images of a cell spheroid. Cell nuclei are fluorescently labelled and appear in white in the video. Scale bar: 50 µm.

File Name: Supplementary Movie 2
Description: Collective dynamics of all cell spheroids. Trajectories are shown as gray lines. Cell spheroids are ordered according to their radius.

File Name: Supplementary Movie 3
Description: Collective dynamics of our model as predicted in the experimental parameter regime. Green vector shows the instantaneous average angular velocity of the cluster. Colored lines show the trajectories of particles.

File Name: Supplementary Movie 4
Description: Collective dynamics of our model as predicted in the low-noise parameter regime. Green vector shows the instantaneous average angular velocity of the cluster. Colored lines show the trajectories of particles.

File Name: Supplementary Movie 5
Description: Collective dynamics of our model as predicted on a sphere where we removed two opposing caps. Green vector shows the instantaneous average angular velocity of the cluster. Colored lines show the trajectories of particles.

File Name: Supplementary Movie 6
Description: Collective dynamics of our model as predicted on a cylinder. Green vector shows the instantaneous average angular velocity of the cluster. Colored lines show the trajectories of particles.

File Name: Supplementary Movie 7
Description: Collective dynamics predicted by an active particle model for a spheroid including bulk particles. Green vector shows the instantaneous average angular velocity of the cluster. Colored lines show the trajectories of particles.
